# Elastomeric PU-Pluronic F‑127 Thin Films: Catalyst and Solvent Effects on Microstructure, Mechanical Properties, and Biocompatibility

**DOI:** 10.1021/acsomega.5c11467

**Published:** 2025-12-08

**Authors:** Oraphan King, Jiraprapa Nirapun, Alongkot Treetong, Chutikorn Phungbun, Panusorn Hunsub, Sagaw Prateepchinda

**Affiliations:** 61191 National Nanotechnology Center (NANOTEC), National Science and Technology Development Agency (NSTDA), Khlong Luang, Pathum Thani 12120, Thailand

## Abstract

While Pluronic F-127 is widely used to create hydrogels, this study investigates its novel application as a semicrystalline soft-segment within elastomeric polyurethane thin films. A series of elastomeric polyurethane-Pluronic F-127 (PU–PF) films was synthesized to investigate the influence of catalyst and solvent selection on the polymer thin film properties. A facile, low-hazard synthesis route using lactic acid as a catalyst and butyl acetate as a solvent (LA_BA) was identified as the optimal synthesis condition. Comprehensive characterization (X-ray diffraction (XRD), atomic force microscopy (AFM), Raman, and differential scanning calorimetry (DSC)) revealed the LA_BA film is a complex, microphase-separated copolymer with 29.29% quasi-crystallinity. The nanostructure consists of a soft, ordered PrePU hard-segment network (modulus ∼20–30 kPa) embedded with stiffer and dendritic Pluronic F-127 domains (∼150–350 kPa). This model, supported by broad XRD peaks and the absence of a melting endotherm (*T*_m_), coexists with a highly mobile, plasticized amorphous phase (*T*_g_ = −45.04 °C). This unique microstructure was found to govern the film’s high performance, affording both superior flexibility (990% elongation) and an oxygen transmission rate (OTR) that exceeded a commercial standard. Furthermore, *in vitro* assays confirmed the LA_BA film is noncytotoxic (ISO 10993–5 standard) and a nonirritant in a three-dimensional (3D) reconstructed human epidermis (RhE) model (OECD 439 standard). The combination of a low-hazard synthesis, a well-defined structure, robust performance, and demonstrated biocompatibility positions the LA_BA film as a strong candidate for advanced medical or cosmeceutical applications, such as wound dressings or transdermal patches.

## Graphical abstract

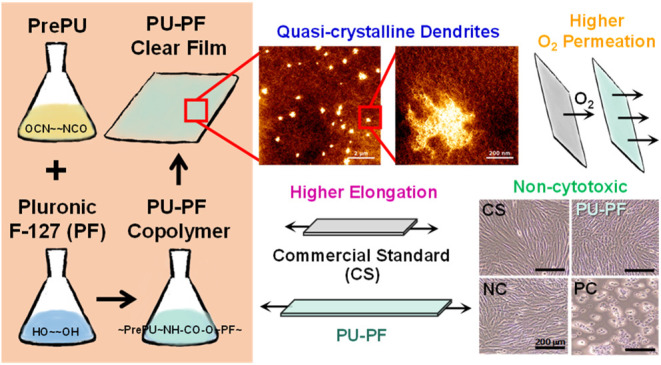

## 1 Introduction

Advancements in synthetic materials science have led to the development of polymers capable of closely mimicking the properties of human skin due to their versatility and broad application potential across medical, cosmetic, and technological fields. Their tunable mechanical, chemical, and biological characteristics make them particularly valuable in applications requiring biocompatibility, flexibility, and durability. In medical contexts, synthetic polymers are employed in skin tissue engineering, where they serve as scaffolds, hydrogels, and nanofibrous matrices to support cell adhesion, proliferation, and tissue regeneration.^1–5^ They also play critical roles in the fabrication of prosthetics, implants, and controlled drug delivery systems, offering customizable performance and targeted therapeutic delivery.^6–9^ In the cosmetic industry, synthetic polymers enhance product stability, control the release of active ingredients, and improve biocompatibility.^10–12^ Additionally, these polymers are central to advancements in robotics and human-machine interfaces, enabling the development of artificial skin with properties such as tactile sensitivity, self-healing capabilities, and electrical conductivity.^13^

Polyurethane (PU) films are versatile materials used in a wide range of industries such as electronics, industrial, automotive, construction, food industry, cosmetic and medical application due to their good mechanical properties, low-temperature flexibility, transparency, high stability, and biocompatibility.^14,15^ PU films are commonly used as an occlusive wound dressing to protect wounds and keep moisture around the wound. Oxygen is essential for a variety of wound healing processes, such as resistance to infection, angiogenesis, collagen deposition, epithelialization, and fibroplasia. Sufficient oxygenation can positively stimulate a healing response of wound tissue.^16^ Therefore, a PU wound dressing with higher oxygen permeability is more desirable. In addition, PU films can be used as transdermal drug delivery for sustained drug release through the skin for clinical and cosmetic purposes due to their ability to control release by adjusting film degradability and swelling behavior.^17–19^

PU film synthesis normally involves the reaction between polyols and isocyanates compounds which the isocyanate groups react with the hydroxyl groups of the polyol, forming urethane linkages that connect the polymer chain. The synthesis steps include mixing the polyols, isocyanate and additive including catalysts or chain extenders with or without solvents at a specific temperature and time to allow complete reaction and a strong cross-linked network formation. Solvent-based processes provide better control over product film properties but require good solvent removal steps. However, solventless processes require more control over reaction conditions but they are more eco-friendly. Many polyurethanes were difficult to be soluble in common organic solvents due to their rigid backbones. A.V. Raghu and co-workers studied polymer structure modification by introducing asymmetric or bulky groups on the pendant polymer backbone or incorporating nonco-planar structural units on the main polymer chain in synthesis condition of dry dimethylformamide at 80 °C.^20^ This synthesis condition requires waterless and high temperature during the process. For film formation, the reaction mixture can be cast onto a mold, and the solvent is evaporated to obtain the final PU film.^21^

In this study, we synthesized polyurethane film using poly­(propylene glycol), tolylene 2,4-diisocyanate terminated (*M_n_* ∼2300) and Pluronic F-127 with different catalysts and solvents as a skin patch for wound dressing or transdermal drug delivery. Pluronic F-127 is a nontoxic, nonionic, and amphiphilic copolymer containing poly­(ethylene oxide) (PEO)-poly­(propylene oxide) (PPO) arranged in a triblock structure to form PEO–PPO–PEO symmetric. It is a unique gel that responds to temperature changes. This special property, along with its safe and biodegradable nature, makes it a valuable tool across various industries. Notably, Pluronic F-127 shines as a drug carrier, enhancing the effectiveness of medications in two ways: it improves how well drugs are absorbed by the body and helps them withstand temperature fluctuations. Importantly, it can also encourage cells to stick together and build collagen, which is crucial for the formation of new blood vessels and tissue repair. This makes Pluronic F-127 particularly useful for researchers working on regenerating tissues with poor blood flow, such as skin, fat, cartilage, tendons, and bones. In fact, scientists have successfully used Pluronic F-127 to encapsulate stem cells, promoting their ability to regenerate these various tissues.^22^

A diverse array of catalysts is employed to drive polymerization of polyurethane film. These catalysts typically include amines, metal complexes, acids, and peroxides, each offering unique advantages in reaction rate and control over polymer structure. The organocatalyst 1,4-diazabicyclo [2.2.2] octane (DABCO) is a bicyclic tertiary amine that operates via nucleophilic and general base activation.^23^ Similarly, 1,5,7-triazabicyclo [4.4.0] dec-5-ene (TBD), a bicyclic guanidine with strong basicity, operates via a bifunctional catalytic mechanism, simultaneously activating both the initiator and the monomer. Dicumyl peroxide (DCP) serves as a radical initiator, decomposing into free radicals that attack monomer molecules and initiate a polymerization chain reaction, resulting in the formation of long polymer chains. In contrast, acetic acid acts as a proton donor catalyst, accelerating reactions by protonating reactant molecules. Lactic acid functions in a similar manner but is milder and less irritating compared to acetic acid.

Thus, this study will evaluate the effects of cross-linking agents including DCP, TBD, DABCO, acetic acid, and lactic acid and polar solvents including tetrahydrofuran, ethyl acetate, and butyl acetate on physical and mechanical properties, cytotoxicity, and skin irritation of the film to find a suitable PU film for potential skin patch and wound dressing uses.

## 2 Materials and Methods

### 2.1 Materials

Poly­(propylene glycol), tolylene 2,4-diisocyanate terminated (*M_n_* ∼2300) (PrePU), Pluronic F-127 (PF127), 1,4-diazabicyclo [2.2.2] octane (DABCO), 1,5,7-triazabicyclo [4.4.0] dec-5-ene (TBD), Celite 545 AW and tetrahydrofuran (THF) were purchased from Sigma-Aldrich. Dicumyl peroxide (DCP) was purchased from Acros Organics, Belgium. Acetic acid (AA), lactic acid (LA), ethanol (EtOH), dichloromethane (DCM), and hexane were purchased from CARLO ERBA Reagents, Italy. Ethyl acetate (EA) was purchased from Fisher Chemical. Butyl acetate (BA) was purchased from Loba Chemie, India. Three M Nexcare Tegaderm polyurethane thin films were used as a commercial wound dressing polyurethane film standard (CS). All chemicals and solvents were used as received. All solvents were analytical grade. In addition, all cell culture materials were purchased from Gibco.

### 2.2 Polymer Synthesis and Film Preparation

An elastomeric PU copolymer thin film was prepared using different cross-linking agents (DCP, TBD, DABCO, AA, and LA) and polar solvents (THF, EA, and BA). This synthesis method was modified from a research work by Lee et al.^24^ Briefly, poly­(propylene glycol), tolylene 2,4-diisocyanate terminated (PrePU) was added into butyl acetate and stirred at room temperature to form a 15 wt % solution. Pluronic F-127 (5 wt % based on PrePU) and lactic acid (5 wt % based on Pluronic F-127) were then added into the solution. The reaction was carried out at room temperature for 48 h. Synthesis methods for other solvents and catalysts were used the same method as for butyl acetate and lactic acid.

The obtained product solution was then purified by filtration and extraction with water and DCM four to five times, followed by the removal of DCM under reduced pressure. When DCP was used as a catalyst, the product was purified by membrane dialysis for 5 h, then filtered, and both the solid and solution fractions were collected.

A specific amount of sample solution was poured into the glass Petri dish (15 × 100 mm^2^) and dried at room temperature. Each thin film was peeled from the glass Petri dish after soaking in water for 1 h and then left to dry on a polyethylene sheet at room temperature until a dried thin film was obtained.

### 2.3 Characterization

The chemical structures of the samples including reactants and obtained thin films were characterized using a Fourier transform infrared spectrometer (FTIR) with an ATR probe (Nicolet 6700 model, Thermo Electron Corporation, WI). Raman spectra of the samples were collected using a confocal Raman microscope (LabRAM HR Evolution, HORIBA, France). The excitation wavelength of 532 nm was focused on the film surface through a 50× long working distance objective lens, with a pinhole size of 100 μm. A diffraction grating of 300 grooves/mm was employed to disperse the Raman signals. Raman shifts in the range of 200–4000 cm^–1^ were recorded with an acquisition time of 10 s and an accumulation of 10 scans per spectrum.

Thermal properties were studied by thermogravimetric analysis (TGA) using a thermal analyzer (STA, NETZSCH/STA 449 F5 Jupiter model, Germany) and differential scanning calorimetry (DSC) using a Shimadzu DSC-60A Plus (Japan). TGA and DSC studies were conducted at a heating rate of 10 °C/min and under a nitrogen gas flow rate of 20 mL/min.

The molecular weight of the synthesized polymers was determined by gel permeation chromatography (GPC) using a Waters e2695 separations module (Waters Corporation) and detected with a Model 3580 Refractive Index (RI) detector (Viscotek). Polystyrene standards (Molecular weight range: 1,220–1,214,000 g/mol) were used for calibration.

### 2.4 Structural and Morphological Analysis

The surface morphology and bulk clarity of the thin films were visualized using an Olympus DSX510 digital microscope (Evident Corporation, Tokyo, Japan). Imaging was performed using bright field, dark field, and polarized light modes to examine the surface texture and the presence of crystalline regions. The crystal structure and degree of crystallinity of the thin films were elucidated using an X-ray diffractometer (XRD, Bruker/D8 Advance, Germany) operated at 40 kV and 40 mA based on Trovati et al.^25^ A Bruker AXS sample holder was used for the measurements. The diffractograms were scanned from 2θ range of 5–60° with a step size of 0.02° and a dwell time of 0.5 s. The resulting peak clusters were deconvoluted using the Peak Fit module in OriginPro 2015 (OriginLab Corporation, Northampton), applying a Gaussian function for symmetrical peaks. The degree of crystallinity was determined by separating the crystalline and amorphous areas of the diffractogram and calculated as a percentage based on the peak area ratio.

The surface morphology of the films was examined using an atomic force microscope (AFM, NanoWizard 3, JPK Instruments, Germany) operated in Quantitative Imaging (QI) mode. A silicon cantilever tip (Micromash model HQ:XSC11/Al BS) with a nominal tip radius of 8 nm, a resonance frequency of 350 kHz, a sensitivity of 12.87 nm/V, and a spring constant of 62.28 N/m was used. Measurements were performed with a contact force of 500 nN, a *z*-range of 1,000 μm, and an image resolution of 256 × 256 pixels. Scan areas of 10 and 1 μm were investigated. The topography and nanomechanical parameters, including slope and adhesion maps, were simultaneously acquired during the scans. To ensure detailed resolution of the underlying phase structure, the samples underwent a surface preparation step involving oxygen plasma etching (Harrick Plasma PDC-002 cleaner) at 30 W power and 0.2 mbar O_2_ pressure. This etching process, consisting of two 20 s steps, was employed to effectively reveal the fine details of phase separation, based on modifications to the method established by Rejek et al.^26^

### 2.5 Swelling Ratio

The thin films (8 × 8 mm^2^) were swollen in absolute ethanol for durations of 1, 2, 4, and 48 h at room temperature. Swelling ratios at each time point were determined using separated and identical samples. These samples were weighed and then desiccated in a vacuum oven at 55 °C for 24 h, after which the thin film swelling ratio (*n* = 3, wet weight/dry weight) was calculated. In addition, a polyurethane film synthesized using lactic acid as a catalyst in butyl acetate was tested for its swelling ratio in various solvent concentrations, including distilled water, 70% ethanol, and absolute ethanol, over time periods of 1, 2, 6, and 24 h at room temperature.

### 2.6 Mechanical Properties of Thin Film

The tensile properties of thin films were measured based on the ASTM D 882–12 standard^27^ using a mechanical tester (Universal testing machine, Instron 5943, US). In brief, six specimens with an approximate thickness of 0.06 mm were cut to 10 × 80 mm^2^. Testing was performed using a 50 N load cell with an initial grip separation of 50 mm at a grip separation rate of 500 mm/min. The Young’s modulus, breaking factor, percent elongation at break, and tensile energy to break were determined from the recorded stress–strain curve in air at room temperature. A commercial wound dressing polyurethane film standard (CS) served as a control.

### 2.7 Oxygen Gas Transmission Rate through Thin Film

The oxygen transmission rate (OTR) of the thin film was measured using a MOCON OX-TRAN model 2/21, following the ASTM D3985 standard.^28^ Briefly, aluminum foil stickers, 12.5 cm in diameter with a 10 cm^2^ hole, were prepared. Three specimens, approximately 0.06–0.07 mm thick, were attached to the stickers. Each sample was mounted as a sealed, semipermeable barrier between two chambers. A nitrogen flow rate of 20 mL/min was applied to one chamber, while the other chamber contained oxygen at 25 °C, 0% relative humidity, and 1 atm pressure. The oxygen transmission rate was determined by measuring the amount of oxygen gas permeating through the film into the nitrogen carrier gas.

### 2.8 Cell Culture Preparation

Human dermal fibroblasts (HDFs), specifically normal human neonatal dermal fibroblasts (ATCC, Cat#PCS-201–010, Manassas, VA), were plated at a seeding density of 30% confluence and cultured in fully supplemented Dulbecco’s modified Eagle medium (FS DMEM) supplemented with 10%v/v fetal bovine serum, 100 units/mL penicillin, and 100 μg/mL streptomycin under humidified conditions at 37 °C and 5% CO_2_. Cells were routinely passaged by enzymatic digestion (trypsin/EDTA) at a 1:3 subcultivation ratio when confluent, up to passage 6, and subsequently cryopreserved in liquid nitrogen.

### 2.9 *In Vitro* Cell Viability Assay

The cytotoxicity of thin film samples was tested according to ISO10993–5 standard.^29^ Briefly, six thin film samples were extracted at a ratio of 0.2 g of sample per 1 mL of FS DMEM at 37 °C and 5% CO_2_ for 24 h. HDFs were then incubated in each extract of the test samples at concentrations of 100%, 50%, and 25% v/v for 24 h in 96-well plates under humidified conditions at 37 °C and 5% CO_2_. A 10% dimethyl sulfoxide (DMSO) solution in FS DMEM served as a negative control. HDF viability was assessed using the MTT assay, where HDFs were stained with 3-(4,5-dimethyl-2-thiazolyl)-2,5-diphenyl-2*H*-tetrazolium bromide (MTT), and DMSO was subsequently added to dissolve the formazan crystals. The absorbance of formazan was measured using a microplate reader at a wavelength of 570 nm, and the percentage of viable cells was calculated using the formula: Cell viability (%) = (Absorbance of sample/Absorbance of HDFs cultured in plain FS DMEM) × 100. HDF morphology was observed using a microscope at 10× magnification.

### 2.10 *In Vitro* Skin Irritation Test

The test was modified from the OECD Test Guideline No. 439,^30^ which is based on the utilization of reconstructed human epidermis (RhE) that mimics the biochemical and physiological properties of human epidermis. The RhE tissues used in this study were LabCyte EPI-MODEL 24 (Japan Tissue Engineering Co., Ltd., Batch: LEC24–220307-A). Briefly, the thin films were cut to the same size as the tissue using a 6 mm punch and sterilized by UV radiation. The RhE tissue was topically treated with 25 μL of deionized (DI) water before the samples were placed onto the tissue surface. DI water served as a negative control, and 5% sodium dodecyl sulfate was used as a positive control. After a 15 min exposure period, the samples were removed, and the tissues were incubated for 24 h. Tissue viability was then measured using the MTT assay.

### 2.11 Statistical Analysis

Results are presented as mean ± standard deviation, with n representing the number of samples analyzed. A two-way analysis of variance (ANOVA) was performed to determine the effects of solvent types and catalysts on the swelling ratio of the synthesized films, followed by the Tukey–Kramer posthoc test for pairwise comparisons. To compare the tensile properties and oxygen permeability of the final LA_BA film and the commercial standard, an independent two-sample *t* test was used. Statistical significance was defined as *p* < 0.05. All statistical analyses were conducted using JMP IN software (4.0.4, SAS Institute, Cary, NC).

## 3 Results and Discussion

### 3.1 Synthesis, Comparative Screening, and Selection of the Copolymer

Polyurethane-Pluronic F-127 (PU–PF) copolymer films were synthesized under mild conditions from a prepolyurethane diisocyanate (PrePU) and the triblock PEO–PPO–PEO copolymer Pluronic F-127. To optimize the film properties, key synthesis parameters including catalyst type (AA, LA, DABCO, DCP, TBD) and solvent (THF, EA, BA) were investigated.

#### 3.1.1 Structural and Thermal Screening

The structures of the copolymers were characterized by ATR-FTIR spectroscopy (Figure 1). The poly­(propylene glycol) tolylene 2,4-diisocyanate (PrePU) starting material spectrum confirmed its polyurethane prepolymer nature, exhibiting characteristic absorption bands for C–H stretching in −CH_3_ (∼2930 cm^–1^), C=O stretching (∼1730 cm^–1^), amide I (∼1596 cm^–1^), amide II (∼1537 cm^–1^), and C–O stretching (∼1090 cm^–1^). Critically, it also showed the strong, characteristic isocyanate (–NCO) functional group at 2270 cm^–1^ at its chain ends.^31^

**Figure 1:**
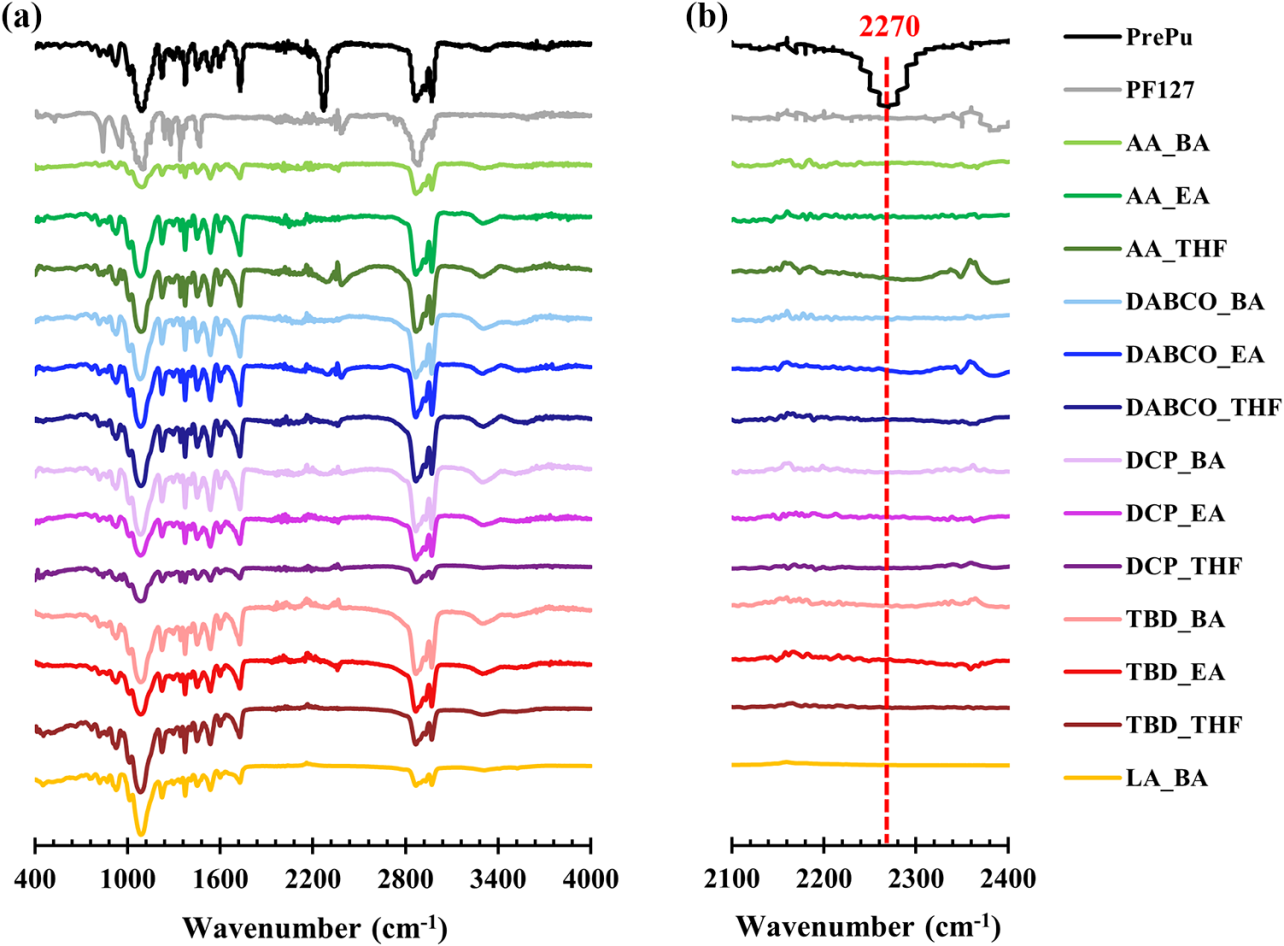
ATR-FTIR spectra of PU–PF films synthesized with various catalysts and solvents compared to precursors (PrePU and Pluronic F-127); (a) Full spectral range (400–4000 cm^–1^) and (b) Expanded region (2100–2400 cm^–1^) highlighting the characteristic isocyanate (–NCO) peak at 2270 cm^–1^ in PrePU and its complete absence in all synthesized PU–PF films, confirming successful polymerization. (Catalysts_Solvents): AA (acetic acid), DABCO (1,4-diazabicyclo [2.2.2] octane), DCP (dicumyl peroxide), TBD (1,5,7-triazabicyclo [4.4.0] dec-5-ene), LA (lactic acid), BA (butyl acetate), EA (ethyl acetate), THF (tetrahydrofuran).

The FTIR spectra of the synthesized PU–PF films confirmed the successful completion of the polymerization. As shown in Figure 1b, the characteristic –NCO peak at 2270 cm^–1^ was completely absent in all samples.^32,33^ This confirms the successful reaction between the PrePU’s isocyanate end-groups and the hydroxyl groups of Pluronic F-127 to form the desired urethane linkages (−NH–C­(=O)–O−), resulting in the final, high-molecular-weight copolymer (Scheme 1).

**Scheme 1:**
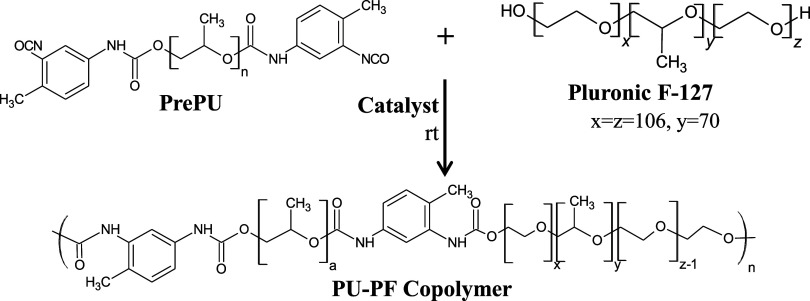
Facile Synthesis of the PU–PF Copolymer from Pre-Polyurethane (PrePU) and Pluronic F-127

Gel permeation chromatography (GPC) was used to determine the molecular weight properties of the copolymers (Table 1). The weight-average molecular weight (*M*_*w*_) of the starting PrePU and Pluronic F-127 were 5,787 g/mol and 20,364 g/mol, respectively, giving a combined starting *M*_*w*_ of approximately 26,000 g/mol. Most formulations produced high-molecular-weight polymers, with *M*_*w*_ values ranging from 47,000 to 230,000 g/mol, confirming highly successful polymerization. However, the TBD_EA and DABCO_EA conditions yielded copolymers with *M*_*w*_ values of 29,748 and 35,624 g/mol, respectively. These molecular weights are significantly lower than those from other formulations, indicating low polymerization efficiency. This inefficiency is attributed to distinct catalyst-solvent incompatibilities.

**Table 1:** GPC-Derived Molecular Weight Data for the Precursors and Synthesized PU–PF Copolymers^a^

sample	*M_n_*	*M_w_*	*M_z_*	PDI
prepolyurethane (precursor)	4,085	5,787	8,556	1.42
Pluronic F-127 (precursor)	19,575	20,364	21,134	1.04
AA_BA	59,093	95,195	149,056	1.61
AA_EA	24,710	160,801	506,687	6.51
AA_THF	52,882	72,313	96,604	1.37
DABCO_BA	21,783	131,607	522,000	6.04
DABCO_EA	13,471	35,624	68,440	2.64
DABCO_THF	8,151	53,108	153,420	6.52
DCP_BA	N/A	N/A	N/A	N/A
DCP_EA	N/A	N/A	N/A	N/A
DCP_THF	15,657	46,978	86,528	3.00
TBD_BA	12,904	51,945	99,611	4.03
TBD_EA	11,706	29,748	55,242	2.54
TBD_THF	41,119	115,449	277,795	2.81
LA_BA	48,915	90,781	153,635	1.86

a Data includes number-average (*M_n_*), weight-average (*M_w_*), Z-average (*M_z_*) molecular weights, and polydispersity index (PDI = *M_w_*/*M_n_*)

In the TBD_EA system, the low *M*_*w*_ is ascribed to a competing side reaction; TBD is a potent organocatalyst for transesterification,^34^ which likely promoted a chain-terminating reaction between the polyol and the ethyl acetate solvent. For the DABCO_EA system, the low efficiency is attributed to solvent polarity. Polyurethane kinetics are known to be highly sensitive to the reaction medium polarity.^35^ The higher polarity of ethyl acetate (compared to butyl acetate) likely suppressed the polymerization rate by interfering with the active catalyst-alcohol complex, whereas the less polar butyl acetate environment facilitated an efficient polymerization.

Thermal stability was assessed by TGA (Figure 2). The precursor materials, PrePU and Pluronic F-127, showed maximum degradation temperatures (*T*_DTG_) at 377.0 and 389.6 °C, respectively. Most PU–PF films exhibited *T*_DTG_ values within this range. The DABCO_BA sample, however, showed the lowest onset of decomposition (*T*_onset_ at 220.2 °C) and *T*_DTG_ (374.6 °C). This reduced thermal stability is attributed to its very broad molecular weight distribution (PDI = 6.04), which suggests a significant fraction of low-molecular-weight oligomers that degrade at lower temperatures.^36^

**Figure 2:**
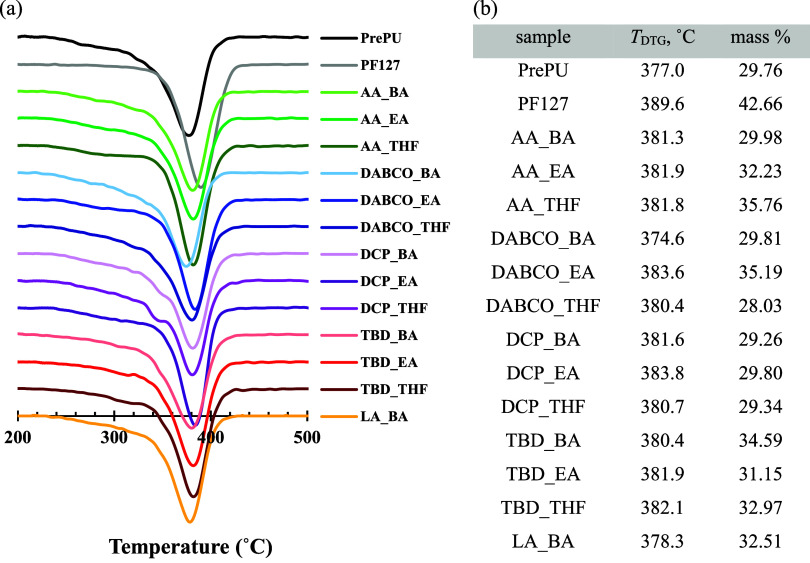
Thermal stability of PU–PF films synthesized with various catalysts and solvents, compared to the Pluronic F-127 (PF127) and prepolyurethane (PrePU) precursors. (a) TG/DTA curves under N_2_ gas atmosphere. (b) Corresponding plot of the temperature of maximum mass loss (*T*_DTG_). (Catalysts_Solvents): AA (acetic acid), DABCO (1,4-diazabicyclo [2.2.2] octane), DCP (dicumyl peroxide), TBD (1,5,7-triazabicyclo [4.4.0] dec-5-ene), LA (lactic acid), BA (butyl acetate), EA (ethyl acetate), THF (tetrahydrofuran).

#### 3.1.2 Swelling Behavior and Dissolution

The swelling ratio of the films in ethanol was assessed over 48 h (Figure 3). This is a critical property, as it relates to the film’s structural integrity and its potential for incorporating active ingredients. The films’ behavior was highly dependent on the catalyst and solvent. The DCP_THF film underwent complete dissolution in ethanol after 1 h, rendering it unsuitable for applications requiring solvent contact. Films synthesized with the DABCO catalyst showed high and unstable swelling, with DABCO_THF increasing its swelling ratio dramatically over time. This instability is likely due to its very high PDI (6.52), which implies a lower effective cross-link density, allowing for excessive solvent penetration. In contrast, films synthesized using acetic acid (AA) and lactic acid (LA) as catalysts (e.g., AA_EA, AA_BA, LA_BA) all exhibited stable, moderate, and controlled swelling over the 48 h period.

**Figure 3:**
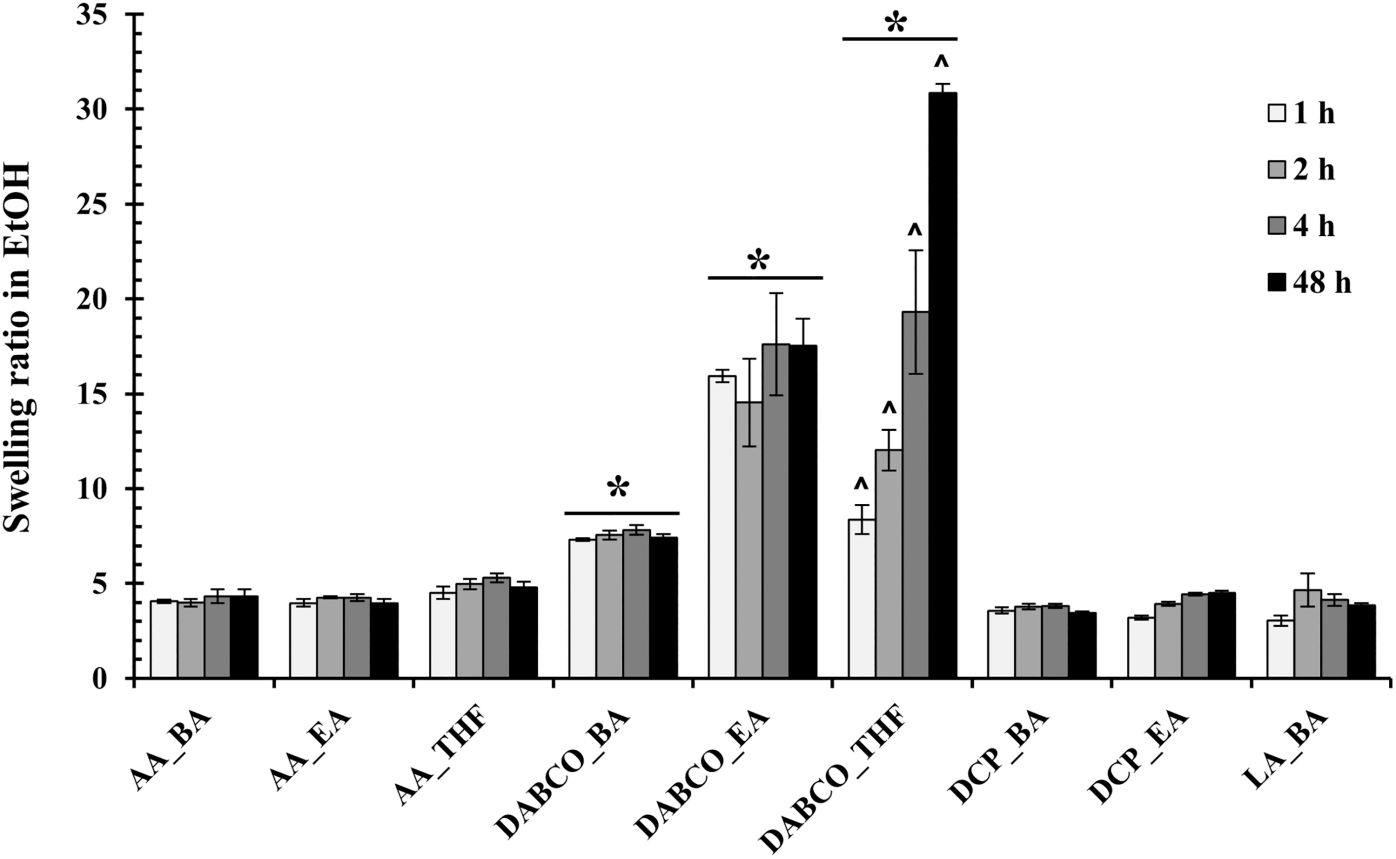
Swelling ratios of all synthesized PU–PF films in absolute ethanol over 48 h. Data are mean ± SD (*n* = 5). *p < 0.05 (comparison between groups); ^∧^ *p* < 0.05 (comparison over time).

#### 3.1.3 Selection of the PU–PF Film

Based on the preceding screening data, the final selection was driven by physical characteristics and the safety profile of the synthesis. While many formulations produced viable polymers, not all yielded usable films. The LA_BA formulation, however, produced a desirable transparent, thin, and homogeneous film (Figure 4). Furthermore, the facile and low-hazard synthesis route was a critical factor. Catalysts such as DCP, DABCO, and AA, along with solvents like THF, are associated with significant health or environmental hazards.^37–43^ In contrast, LA and TBD is a nontoxic catalyst^44,45^ and butyl acetate (BA) is a lower-hazard solvent.^46,47^ Therefore, the LA_BA film was selected for in-depth characterization as it exhibited robust physicochemical properties while also meeting the key objectives for physical appearance and a low-hazard profile.

**Figure 4:**
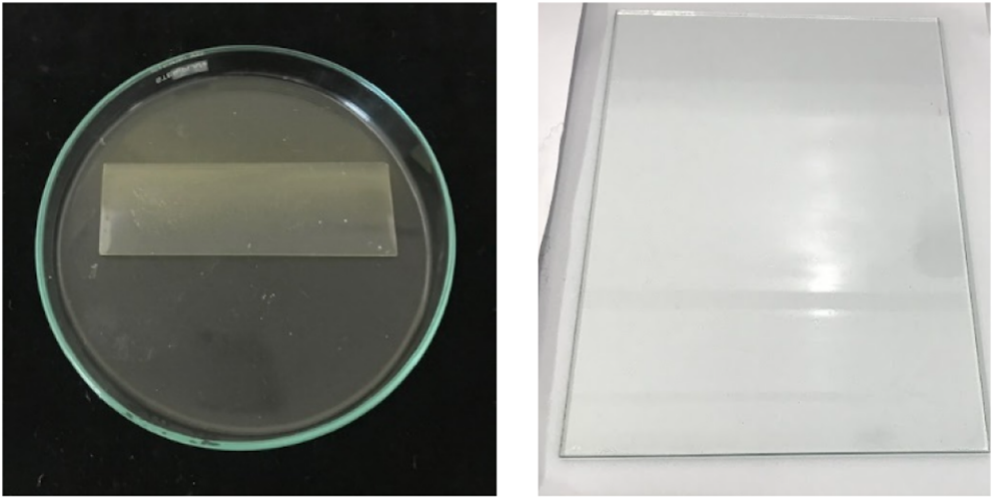
Physical appearance of obtained polyurethane thin film, LA_BA with clear thin film.

### 3.2 Physicochemical and Microstructural Characterization of the LA_BA Film

Optical microscopy confirmed the high macroscopic quality of the film, with bright-field and dark-field images showing a clear and smooth surface (Figure 5a,b). Crucially, the polarized light modes revealed the presence of small, birefringent domains scattered throughout the film, some with a distinct triangular morphology (Figure 5d), providing the first visual evidence of a semicrystalline, phase-separated morphology. This structure was then investigated in detail using multiple analytical techniques.

**Figure 5:**
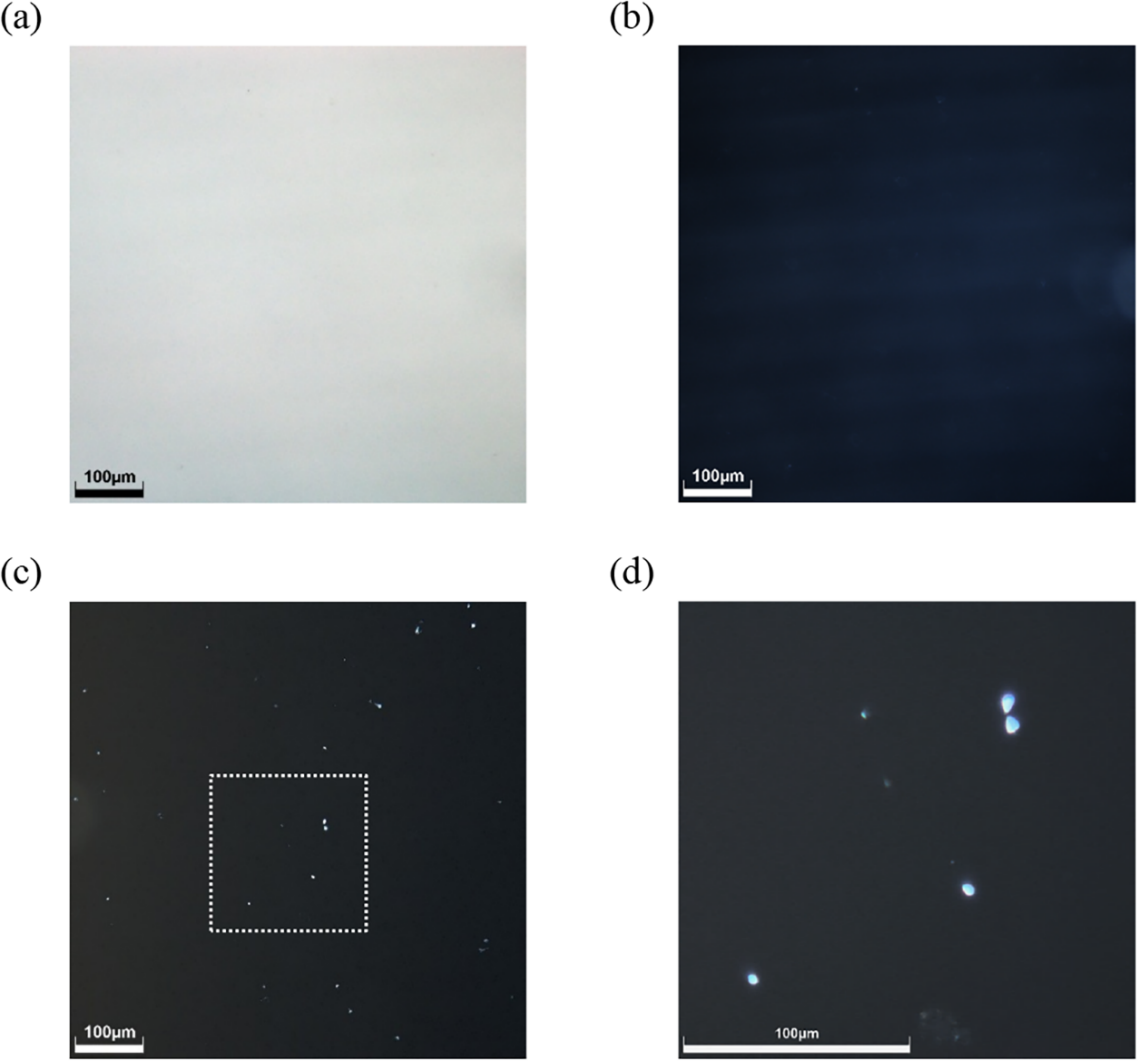
Optical micrographs of the LA_BA thin film. (a) Bright-field illumination. (b) Dark-field illumination. (c, d) Polarized light illumination, revealing small crystalline domains. All images were captured using 20× objective lens with digitally enhanced magnification. Scale bar = 100 μm.

X-ray diffraction (XRD) was used to investigate the bulk crystalline structure of the final LA_BA film compared to the precursors (Figure 6). The diffractogram of the pure Pluronic F-127 exhibited two strong, sharp crystalline peaks at 2θ = 19.095° (*hkl* = 120) and 2θ = 23.229° (*hkl* = 200), characteristic of its semicrystalline PEO chains (Figure 6b).^48,49^ In contrast, the PrePU exhibited broad amorphous halos at 2θ angles around 14.500°, 20.550°, and 43.000° (Figure 6a),^25^ indicating its predominantly amorphous nature. In the final LA_BA film, these sharp peaks were replaced by a single, broad, and complex peak envelope (2θ = 14°–25°). Peak deconvolution confirmed these peaks originated from the PEO blocks (Figure 6c), but their significant broadening and merging indicated that the bulk crystallization was physically restricted, leading to smaller, less-ordered, or strained crystalline domains. This interpretation is supported by the quantitative analysis, which determined the final bulk degree of crystallinity to be 29.29%.

**Figure 6:**
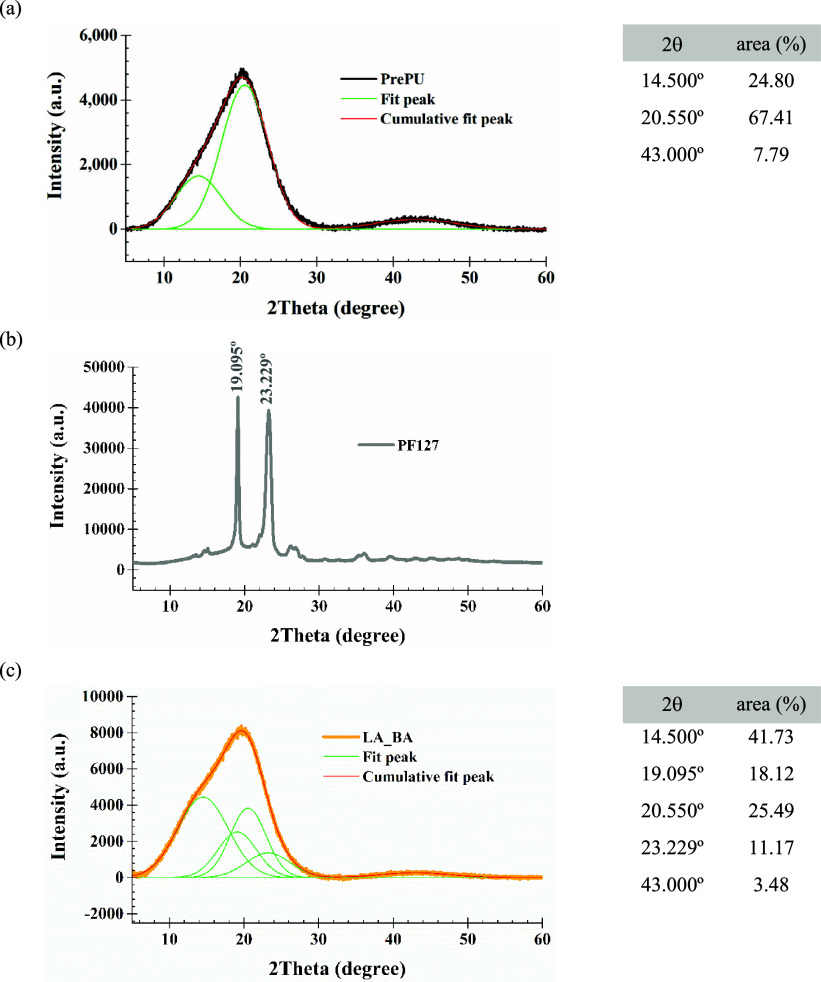
X-ray diffraction (XRD) patterns of (a) PrePU, (b) Pluronic F-127 (PF127), and (c) the LA_BA copolymer over the 2θ range of 5–60°. The solid lines in (a, c) represent the deconvoluted peaks used for crystallinity calculations.

Atomic force microscopy (AFM) (Figure 7) was employed to resolve this complex, hierarchical morphology. The as-prepared (unetched) film exhibited a relatively smooth surface, but its height and phase images confirmed the presence of the triangular, plate-like domains observed by OM, consistent with crystal growth at the film–air interface (Figure 7d). The underlying bulk nanostructure was revealed using controlled O_2_ plasma etching for depth profiling.^26^ The postetching morphology (Figure 7e,f) revealed a different structure: a fibrous network, identified as the ordered PrePU hard segments, which contained distinct, embedded domains of Pluronic F-127.

**Figure 7:**
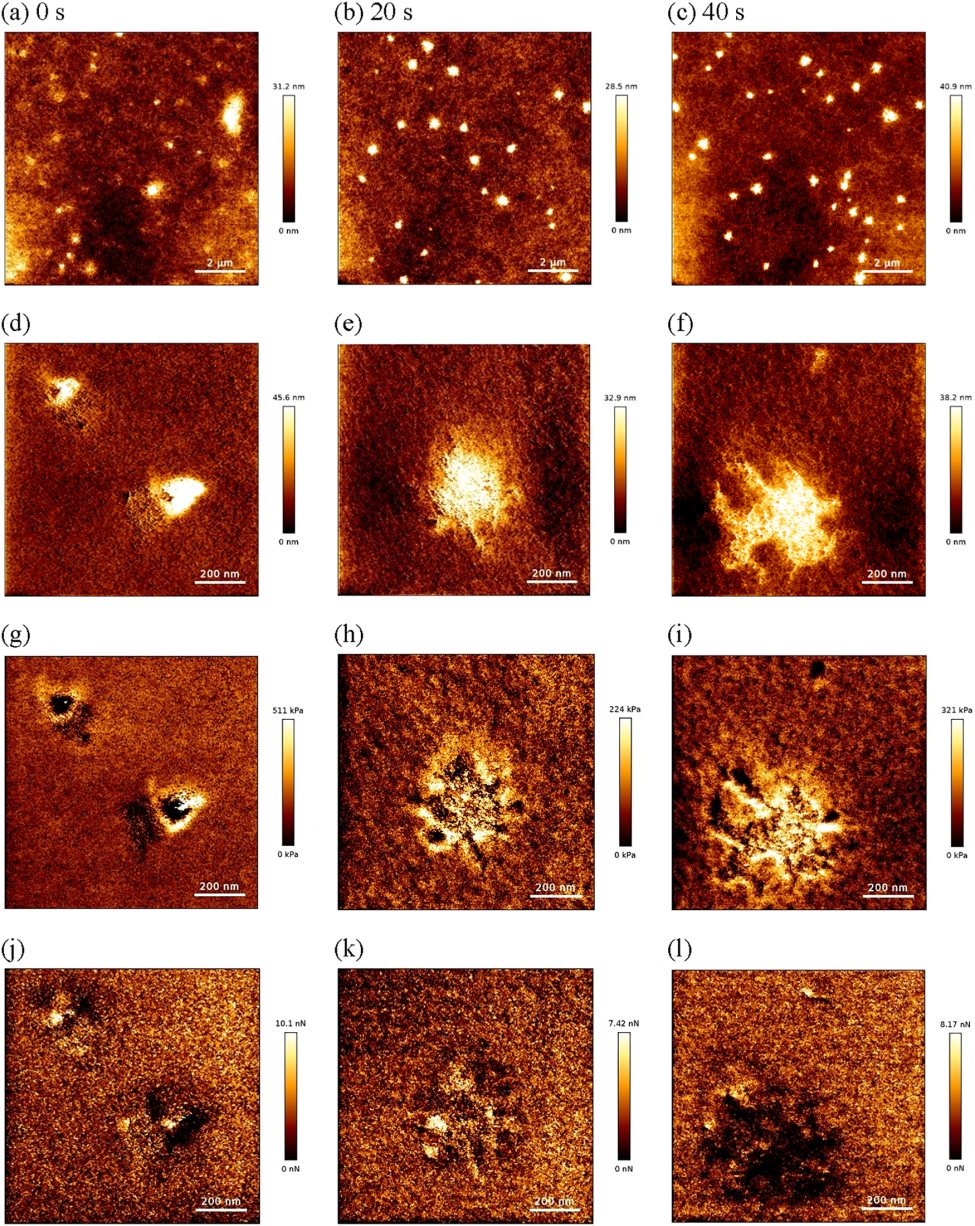
AFM analysis of the LA_BA film, revealing the nanostructure and nanomechanical properties as a function of O_2_ plasma etching time. Columns (left to right): 0 s (as prepared), 20 s, and 40 s of O_2_ plasma etching. Large-area (10 × 10 μm^2^) height topography (a–c). High-magnification (1 × 1 μm^2^) height topography (d–f). Young’s Modulus maps (g–i). Adhesion maps (j–l). Scale bars = 2 μm for (a–c) and 200 nm for (d–l). The modulus and adhesion maps (g–l) correspond to the 1 × 1 μm^2^ scan area.

Nanomechanical and physicochemical mapping of the etched surface provided quantitative corroboration for this model. This analysis was necessary because the as-prepared film (modulus ∼1.8–2.5 MPa) exhibited a smooth, phase-mixed surface skin that masked the underlying components. The maps of the exposed nanostructure revealed a clear hierarchy of properties. The Young’s Modulus map confirmed that the Pluronic domains formed branched, dendritic structures with a complex internal modulus distribution (Figure 7h,i), similar to those observed in other Pluronic-polymer systems.^50^ These dendrites exhibited a very low modulus (soft) core, consistent with trapped amorphous PEO, but showed the highest elastic modulus (∼150–350 kPa) at the corners and tips of their branches. This signature is characteristic of dendritic crystallization, where the stiff tips represent the active, ordered growth fronts. In contrast, the interconnected PrePU fibrous network was confirmed to be an intermediate component, exhibiting a modulus of ∼20–30 kPa. Complementing this, the adhesion maps showed that these same high-modulus dendritic domains exhibited significantly low surface adhesion (Figure 7l). This finding is consistent with their highly ordered, quasi-crystalline nature, as these rigid, constrained chains are less adhesive than the surrounding, more mobile amorphous matrix.

Raman microspectroscopy (Figure 8) provided the definitive chemical basis for this microheterogeneity, supporting the AFM findings. Following the methodology for analyzing multiphase polyurethanes,^51^ chemical phase separation was confirmed by identifying distinct fingerprint peaks for each component. The hard-segment domains (PrePU) were identified by the strong, characteristic aromatic C=C ring vibration at 1621.31 cm^–1^. The amorphous, phase-mixed matrix was identified by the free C=O stretching vibration at 1717.79 cm^–1^. The soft-segment domains (Pluronic) were identified by their strong C–H stretching signals (2800–3000 cm^–1^).

**Figure 8:**
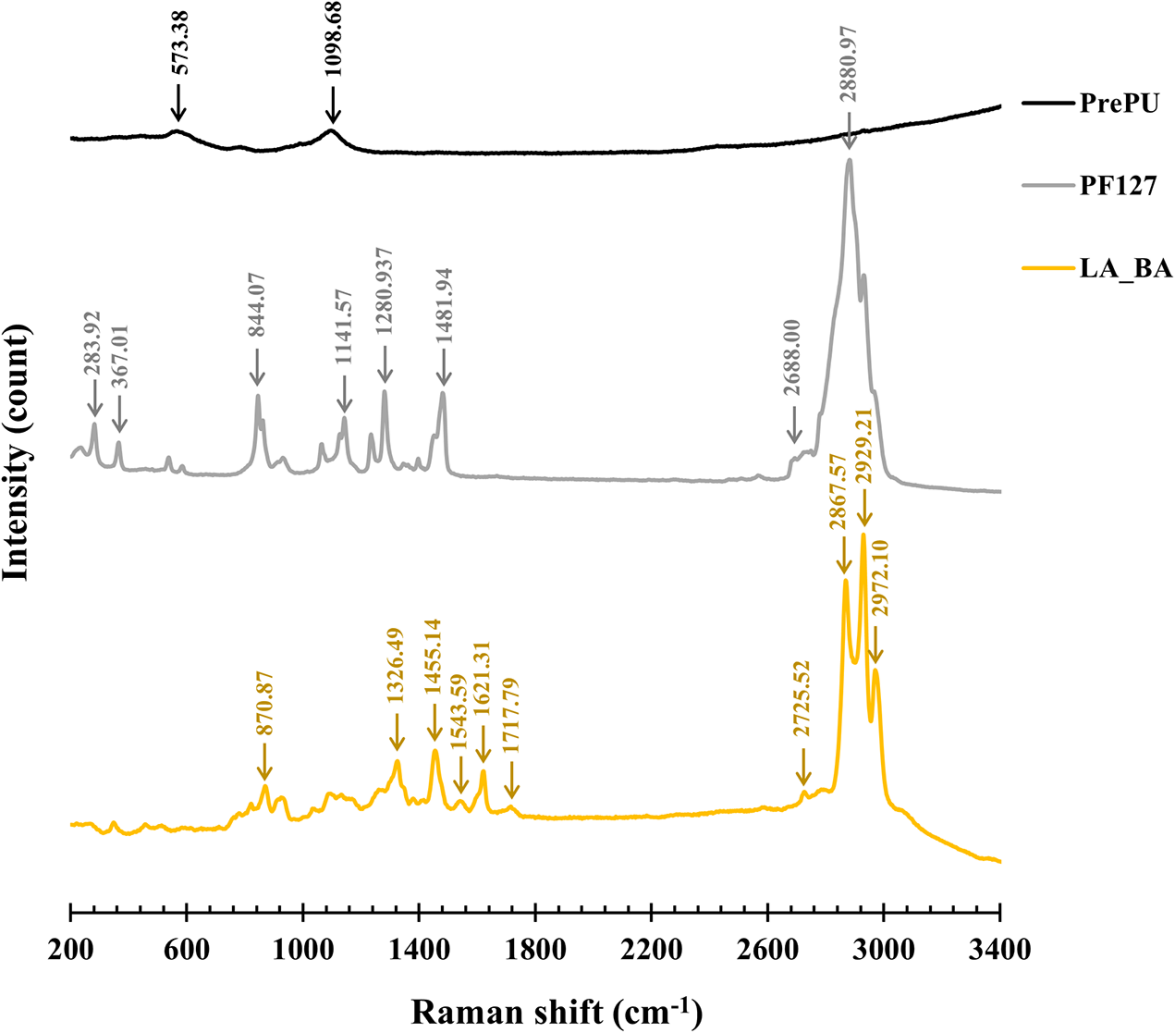
Raman spectra of the LA_BA copolymer and its precursors, PrePU and Pluronic F-127 (PF127).

A significant finding was observed in this C–H region. The pure Pluronic F-127 (which contains bulk crystals) exhibited a single, high-intensity peak at 2880.97 cm^–1^. In the final LA_BA film, this peak was replaced by three distinct, sharp, but lower-intensity peaks at 2867.57, 2929.21, and 2972.10 cm^–1^. This peak splitting indicates an enhancement in conformational order as the PEO chains are locked into the rigid dendritic structure. However, the concurrent loss of signal intensity is characteristic of nanoscale domains. As demonstrated by Patel et al.,^52^ as crystal size decreases, the volume fraction of the disordered surface layer increases, which consequently, weakens the Raman intensity. This combined finding strongly supports the nanoscale, quasi-crystalline, dendritic nature of the Pluronic F-127 domains.

The bulk thermal behavior of the LA_BA film was analyzed by DSC (Figure 9). The thermogram exhibited a single glass transition temperature (*T*_g_) at −45.04 °C, confirming that the amorphous components of the PrePU and the Pluronic F-127 are highly phase-mixed. Critically, this *T*_g_ is significantly lower than that of either precursor (PrePU *T*_g_ = −0.51 °C; Pluronic *T*_g_ = −16.14 °C). This depression in *T*_g_ indicates that the mixing of the amorphous segments has increased the overall chain mobility, effectively plasticizing the amorphous phase.^53^

**Figure 9:**
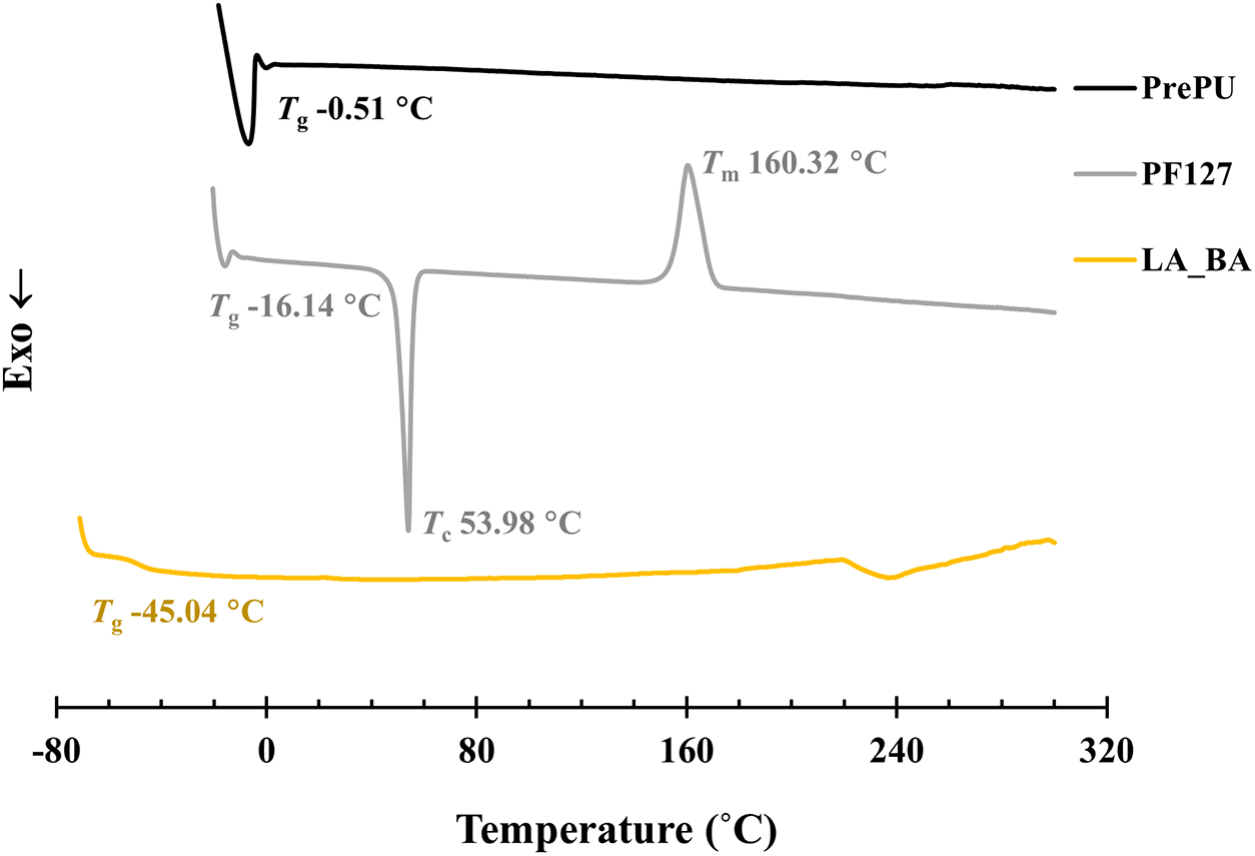
Differential Scanning Calorimetry (DSC) thermograms of PrePU, Pluronic F-127 (PF127), and the LA_BA copolymer, measured under a N_2_ atmosphere.

Furthermore, no distinct melting (*T*_m_) endotherm was observed. This lack of a detectable *T*_m_ peak does not contradict the 29.29% crystallinity calculated from XRD. Instead, it indicates that the crystalline domains are small, imperfect, or physically constrained by the surrounding, highly mobile amorphous matrix, causing their melting transition to be too broad or weak to be detected by conventional DSC.

Taken together, these data confirm a complex, hierarchical, and semicrystalline structure. The film consists of a smooth surface skin, but its bulk is microphase-separated. The Raman and AFM data confirmed the presence of two distinct ordered phases: an interconnected PrePU hard-segment network and embedded, dendritic Pluronic F-127 domains. This dendritic, quasi-crystalline morphology is strongly supported by the broad, merged XRD peaks, the nanoscale-induced intensity loss in the Raman C–H peaks, and the absence of a *T*_m_ in the DSC. The DSC results complete this bulk model, showing that while these quasi-crystalline domains have separated, the remaining amorphous domains have phase-mixed to form a single, plasticized phase with a low *T*_g_ of −45.04 °C.

### 3.3 Comparative Performance and Functional Properties

Following the in-depth microstructural characterization, the functional performance of the LA_BA film was evaluated to determine its suitability for the intended application. A material’s viability is not only dependent on its nanoscale structure but also on its macroscopic performance, particularly in relation to established benchmarks. Therefore, the LA_BA film was benchmarked against a commercial standard (CS) polyurethane wound dressing. This comparative analysis focused on three critical performance metrics: mechanical integrity (tensile properties), physiological gas exchange (oxygen permeability), and environmental stability (swelling behavior).

#### 3.3.1 Mechanical Properties

The tensile properties of the LA_BA thin film and the commercial standard (CS) are illustrated in the stress–strain curves (Figure 10). Both films exhibit a sigmoidal stress–strain relationship characteristic of elastomeric, rubber-like behavior, with fracture occurring at very high strains (>500%).^54^ The key mechanical properties derived from these curves (modulus, tensile strength, percent elongation at break, and tensile energy to break) are summarized in Table 2. The CS film demonstrated a significantly higher modulus, tensile strength, and tensile energy to break, indicating it is a considerably stiffer material. Conversely, the LA_BA film exhibited a significantly greater percent elongation at break (990%) compared to the CS film (640%). This exceptional flexibility and stretchability are consistent with the material’s highly mobile, plasticized amorphous phase, which was previously confirmed by its low glass transition temperature (*T*_g_ = −45.04 °C) (Figure 9).^53,55^

**Figure 10:**
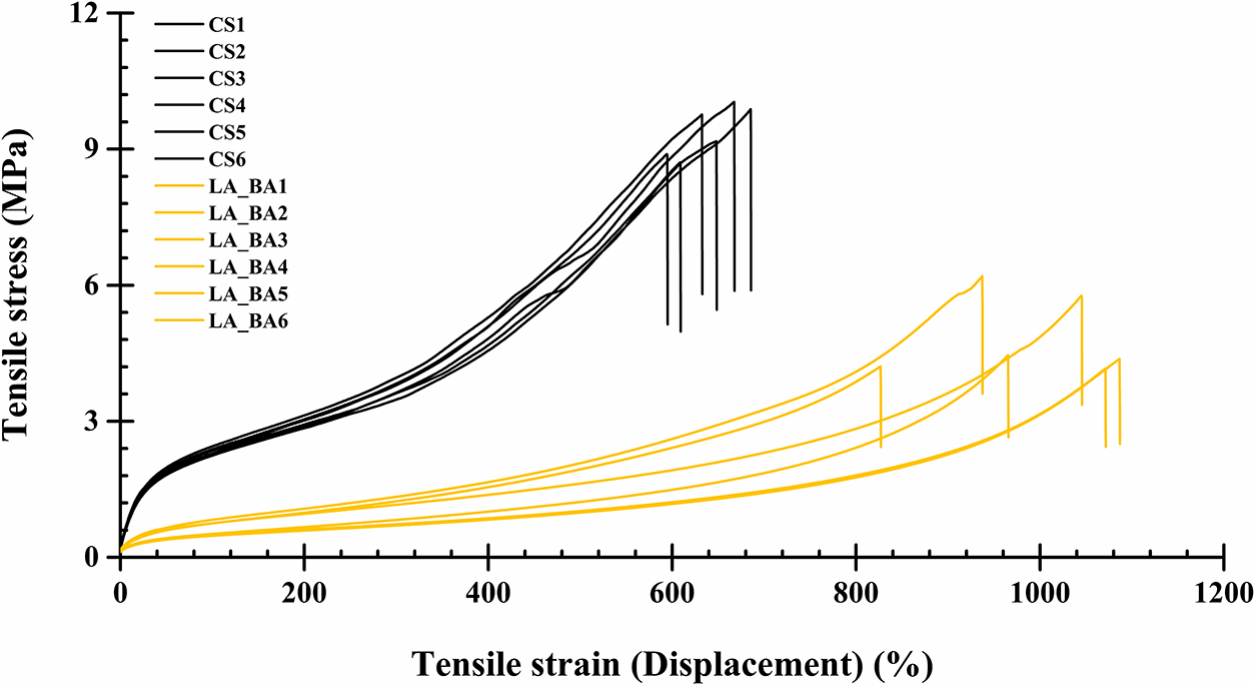
Representative stress–strain curves for the commercial polyurethane wound dressing film standard (CS) and the synthesized LA_BA polyurethane film (LA_BA). (*n* = 6).

**Table 2:** Tensile Properties of Polyurethan Films^a^

sample	thickness (mm)	modulus (tangent 3%) [MPa]	modulus (secant 3%) [MPa]	tensile strength (MPa)	%elongation at break	tensile energy to break (J)
CS	0.063 ± 0.00	7.57 ± 0.32*	14.30 ± 0.56*	9.41 ± 0.56*	640.00 ± 34.61	0.93 ± 0.10*
LA_BA	0.072 ± 0.01	1.84 ± 0.53	6.88 ± 1.04	4.86 ± 0.89	990.00 ± 99.05*	0.67 ± 0.11

a Note: CS is a commercial wound dressing polyurethane film standard. LA_BA is polyurethane film synthesized by using lactic acid as a catalyst in butyl acetate. (*n* = 6, *between groups, *p* < 0.05).

#### 3.3.2 Oxygen Permeability

Oxygen permeability is a key performance metric for wound dressings, as oxygen is critical for various wound healing mechanisms.^56^ The oxygen transmission rate (OTR) of the LA_BA thin film was measured and compared to the commercial standard (CS), as shown in Figure 11. The LA_BA film exhibited a significantly greater OTR than the CS film. This superior gas transport property is a direct consequence of the film’s complex microstructure, as revealed by AFM and DSC. Gas transport in semicrystalline polymers occurs exclusively through the mobile, amorphous phase. The AFM nanomechanical maps (Figure 7) confirmed that the ordered, quasi-crystalline components—the high-modulus Pluronic dendrites (∼150–350 kPa) and the intermediate-modulus PrePU network (∼20–30 kPa)—act as physical barriers.

**Figure 11:**
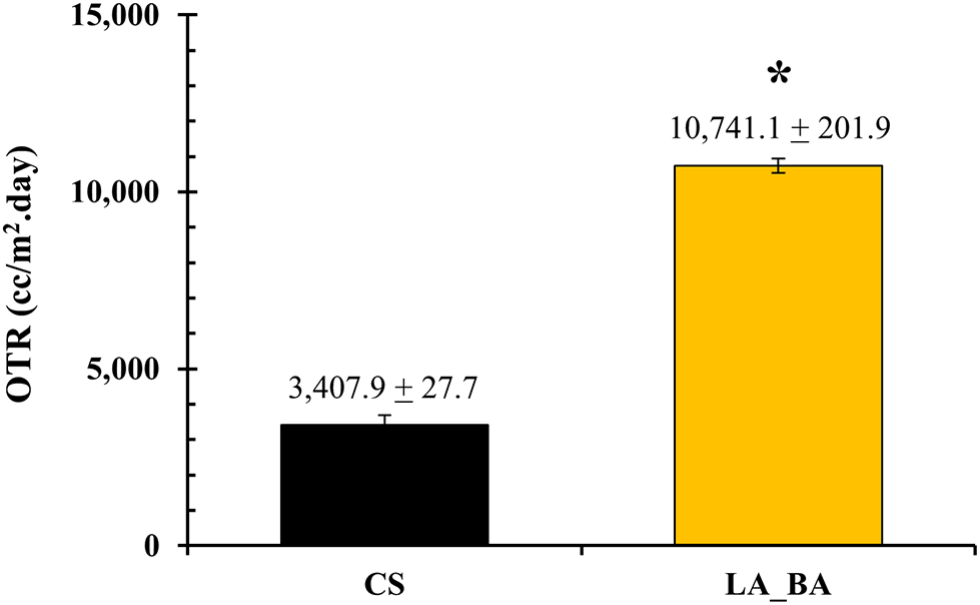
Oxygen transmission rate (OTR) of the synthesized LA_BA film compared to the commercial standard (CS). Data are mean ± SD (*n* = 3). *p < 0.05 (significantly higher than CS).

Crucially, these ordered domains are embedded within a continuous, low-modulus amorphous matrix. This amorphous phase was confirmed by DSC to be highly mobile and plasticized (*T*_g_ = −45.04 °C), providing an extensive and well-interconnected pathway for O_2_ molecules to diffuse. The high OTR is a direct result of this continuous, mobile amorphous phase. This finding indicates that the LA_BA thin film holds promise for application as a wound dressing film.

#### 3.3.3 Swelling Behavior

The swelling behavior of the LA_BA thin film was investigated across varying ethanol concentrations (Figure 12) to assess its stability and solvent interaction. The film’s response was highly dependent on solvent polarity, reaching stable equilibrium swelling ratios that remained constant for the 24 h test period. These equilibrium ratios were approximately 1, 4, and 5 in 0%, 70%, and 100% (v/v) aqueous ethanol, respectively. The minimal swelling in pure water (0% ethanol) confirms the predominantly hydrophobic nature of the LA_BA film. This observation is consistent with the AFM-derived model (Figure 7), which shows a continuous, fibrous network of PrePU hard segments. This hydrophobic network effectively shields the embedded hydrophilic Pluronic F127 domains from the aqueous environment. Conversely, the significantly higher swelling ratios in 70% and 100% ethanol indicate that the less-polar organic solvent can more effectively penetrate and plasticize this hydrophobic PrePU network, leading to greater solvent uptake.

**Figure 12:**
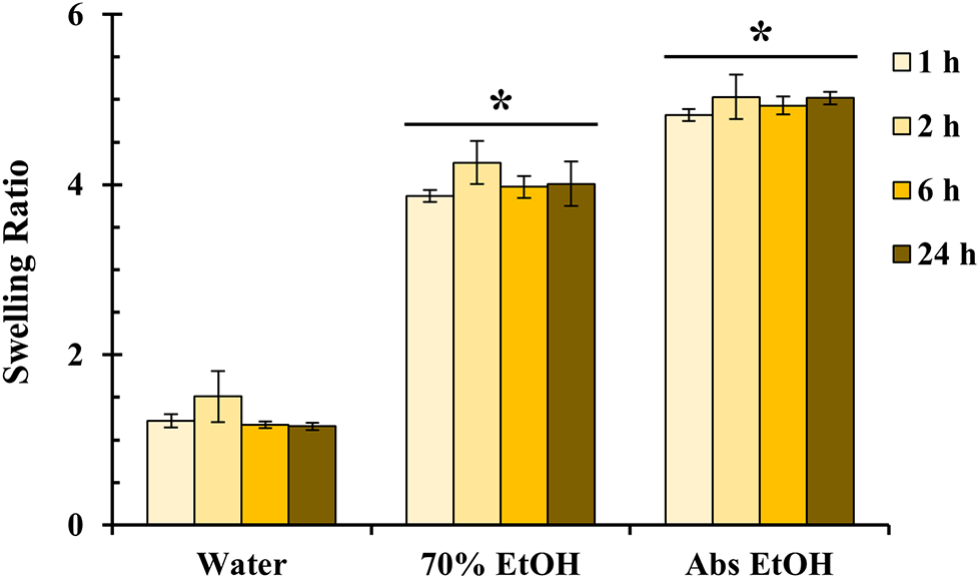
Swelling ratio of the LA_BA film in various aqueous ethanol concentrations (water, 70% EtOH, and abs. EtOH) over 24 h. Data are mean ± SD (*n* = 5). *p < 0.05 (comparison between ethanol concentrations).

### 3.4 Biocompatibility and Safety Profile

After establishing the functional performance of the LA_BA film, its biocompatibility and safety profile were evaluated. These are critical parameters for any material intended for direct skin contact and medical uses. This assessment was performed using established *in vitro* models for cytotoxicity and skin irritation, benchmarking the LA_BA film against the commercial standard (CS) film.

#### 3.4.1 Cytotoxicity

The cytotoxicity of the LA_BA film extracts was assessed in accordance with ISO 10993–5 standard using both quantitative (MTT assay) and qualitative (cell morphology) methods.^29^ For the quantitative assessment, a cell viability below 70% was defined as cytotoxic. A 10% dimethyl sulfoxide (DMSO) solution, which induces cell death, was used as the positive control, while the cell culture media (FS DMEM) served as the negative control.

The results (Figure 13) indicate that the cell viability for all tested PU–PF films (CS and LA_BA) remained well above the 70% noncytotoxic threshold. The qualitative assessment (Table 3) supported this finding, as cells exposed to the film extracts exhibited a normal, spindle-shaped morphology, with no more than 20% of cells showing a round, nonviable shape. These findings confirm that the LA_BA film is noncytotoxic to human skin cells.

**Figure 13:**
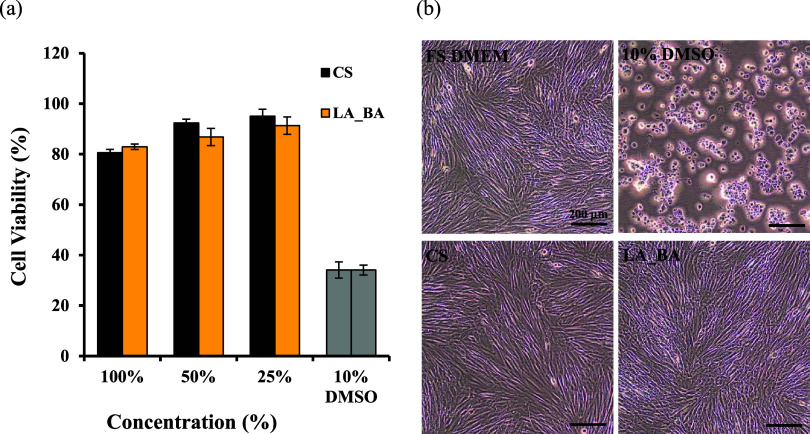
Biocompatibility of synthesized films. (a) Cell viability of human dermal fibroblasts (HDFs) assessed by MTT assay after 24 h incubation with film extracts. Data are mean ± SD (*n* = 5). (b) Representative micrographs of HDF morphology. Viable cells show a spindle shape, while nonviable cells are rounded. Scale bar = 200 μm (10×). Sample codes: FS DMEM (Negative Control), 10% DMSO (Positive Control), CS (Commercial film extract), LA_BA (Film extract).

**Table 3:** Qualitative Morphological Grading of Cytotoxicity of Extracts

grade	reactivity	conditions of all cultures
0	none	Discrete intracytoplasmatic granules, no cell lysis, no reduction of cell growth.
1	slight	Not more than 20% of the cells are round, loosely attached and without intracytoplasmatic granules, or show changes in morphology; occasional lysed cells are present; only slight growth inhibition observable.
2	mild	Not more than 50% of the cells are round, devoid of intra cytoplasmatic granules, no extensive cell lysis; not more than 50% growth inhibition observable.
3	moderate	Not more than 70% if the cell layers contain rounded cells or are lysed; cell layers not completely destroyed, but more than 50% growth inhibition observable.
4	severe	Nearly complete or complete destruction of the cell layers.

#### 3.4.2 Skin Irritation

The skin irritation potential was then assessed using an *in vitro* Reconstructed human Epidermis (RhE) model, following OECD Test Guideline 439.^57^ A classification of nonirritant is assigned when the average tissue viability exceeds 50% (Table 4). As shown in Table 5, the RhE tissue exposed to the LA_BA thin film exhibited an average viability of 100.48%. This result is well above the 50% threshold and is comparable to the negative control, leading to a definitive classification of nonirritant. This finding strongly suggests the LA_BA film is safe for direct application to human skin. While these *in vitro* results are excellent, further safety evaluations would be necessary for its final use as a clinical wound dressing.

**Table 4:** Irritation Test Result Interpretation for Data in Table 5

*in vitro* result	prediction
mean tissue viability >50%	no category (nonirritant)
mean tissue viability ≤ 50%	UN GHS Cat. 1 or 2 (irritant)

**Table 5:** Tissue Viability of RhE after Testing with the Thin Film^a^,^b^

	tissue viability (%)				
sample	tissue 1	tissue 2	tissue 3	mean ± SD	result interpretation
NC	100.04	97.83	102.13	100 ± 2.149	N/A
PC	1.98	2.20	1.76	1.98 ± 0.220	irritant
LA_BA	100.26	104.22	96.95	100.48 ± 3.641	nonirritant

a The results were shown as a mean value from three tissues (*n* = 3).

b NC is DI water. PC is 5% sodium docecyl sulfate. LA_BA is polyurethane film synthesized by using lactic acid as a catalyst in butyl acetate.

## 4 Conclusions

This study investigated the influence of catalyst and solvent selection on the properties of polyurethane-Pluronic F-127 (PU–PF) copolymer films. The LA_BA polymer thin film, synthesized using a low-hazard route with lactic acid (catalyst) and butyl acetate (solvent), was identified as the lead candidate based on its successful polymerization, high thermal stability, and controlled swelling behavior.

A comprehensive, multitechnique characterization (OM, XRD, AFM, Raman, and DSC) elucidated a complex, semicrystalline (29.29%) and microphase-separated structure. Raman spectroscopy provided the chemical “fingerprint” for this morphology, identifying the aromatic PrePU hard segments (1621 cm^–1^) as a distinct phase from the disordered amorphous matrix (1717 cm^–1^). The nanostructure, corroborated by AFM, is composed of two primary ordered components: a fibrous PrePU hard-segment network (modulus ∼20–30 kPa) and embedded, dendritic Pluronic F-127 domains. These quasi-crystalline dendrites exhibited the highest modulus at their branch tips (modulus ∼150–350 kPa). This morphology is strongly supported by the broad, merged XRD peaks, the Raman C–H peak splitting and intensity loss, and the notable absence of a melting endotherm (*T*_m_) in the DSC. Concurrently, DSC analysis confirmed that the remaining amorphous domains are highly phase-mixed, resulting in a single, plasticized phase with a low *T*_g_ of −45.04 °C.

This well-defined, two-phase microstructure dictates the film’s functional properties. The continuous, mobile amorphous phase provides an efficient pathway for gas transport, resulting in an oxygen transmission rate (OTR) that exceeds the commercial standard. The elastomeric network, consistent with the low *T*_g_, provides high flexibility (990% elongation at break).

Furthermore, the LA_BA film was confirmed to be noncytotoxic (ISO 10993–5 standard) and a nonirritant in a 3D reconstructed human epidermis (RhE) model (OECD 439 standard). The combination of its robust mechanical properties, functional permeability, and demonstrated *in vitro* safety indicates the LA_BA film is a viable candidate for advanced cosmeceutical or medical applications, such as transdermal patches or breathable wound dressings.

## Supplementary Material



## Notes

PU–PF films images (Figure S1); GPC chromatograms of PU–PF films and their precursors (Figures S2–S14); X-ray diffraction (XRD) patterns (Figures S15–S29); AFM analyses of nanostructure and nanomechanical properties (Figures S30–S42); Raman spectra (Figure S43); thermal analysis including DSC thermograms and glass transition temperature data (Figure S44); oxygen transmission rate comparison (Figure S45) (PDF)

## †

NSTDA Characterization and Testing Service Center (NCTC), National Science and Technology Development Agency (NSTDA), Khlong Luang, Pathum Thani 12120, Thailand

## ‡

Faculty of Agricultural Product Innovation and Technology, Srinakharinwirot University, Ongkharak, Nakhonnayok 26120, Thailand

## Abbreviations

AA: acetic acid
AFM: atomic force microscope
ANOVA: analysis of variance
BA: butyl acetate
CS: a commercial wound dressing polyurethane film standard
DABCO: 1,4-diazabicyclo [2.2.2] octane
DCM: dichloromethane
DCP: dicumyl peroxide
DI: deionized
DMSO: dimethyl sulfoxide
DSC: differential scanning calorimetry
EA: ethyl acetate
EtOH: ethanol
FS DMEM: fully supplemented Dulbecco’s modified Eagle medium
FTIR: Fourier transform infrared spectrometer
GPC: gel permeation chromatography
HDFs: human dermal fibroblasts
LA: lactic acid
MTT: 3-(4,5-dimethyl-2-thiazolyl)-2,5-diphenyl-2*H*-tetrazolium bromide
*M*_*n*_: number-average molecular weight
*M*_*w*_: weight-average molecular weight
*M*_z_: Z-average molecular weight
OM: optical microscope
OTR: oxygen transmission rate
PDI: polydispersity index
PEO: poly­(ethylene oxide)
PF127: pluronic F-127
PPO: poly­(propylene oxide)
PrePU: poly­(propylene glycol), tolylene 2,4-diisocyanate terminated
PU: polyurethane
PU–PF: polyurethane-Pluronic F-127 copolymer
Raman: Raman microspectroscopy
RhE: reconstructed human epidermis
TBD: 1,5,7-triazabicyclo [4.4.0] dec-5-ene
*T*_c_: crystallization temperature
*T*_DTG_: the temperature corresponds to the maximum of mass losses
*T*_g_: glass transition temperature
TGA: thermogravimetric analysis
THF: tetrahydrofuran
*T*_m_: melting temperature
XRD: X-ray diffraction
