# Supplementary material for: Elastomeric PU-Pluronic F‑127 Thin Films: Catalyst and Solvent Effects on Microstructure, Mechanical Properties, and Biocompatibility
Source: ACS Omega. 2025 Dec 8;10(50):62380–94. doi: 10.1021/acsomega.5c11467 (PMC12750213; doi:10.1021/acsomega.5c11467)
Supplement: Supplementary file 1 [file ao5c11467_si_001.pdf]

Elastomeric PU-Pluronic F-127 Thin Films: Catalyst and Solvent Effects on Microstructure, Mechanical Properties, and Biocompatibility

Oraphan King, Jiraprapa Nirapun[†]*, Alongkot Treetong, Chutikorn Phungbun, Panusorn Hunsub*[‡]*, and Sagaw Prateepchinda**

National Nanotechnology Center (NANOTEC), National Science and Technology Development Agency (NSTDA), Khlong Luang, Pathum Thani, 12120, Thailand

[†] NSTDA Characterization and Testing Service Center (NCTC), National Science and Technology Development Agency (NSTDA), Khlong Luang, Pathum Thani, 12120, Thailand

[‡] Faculty of Agricultural Product Innovation and Technology, Srinakharinwirot University, Ongkharak, Nakhonnayok, 26120, Thailand

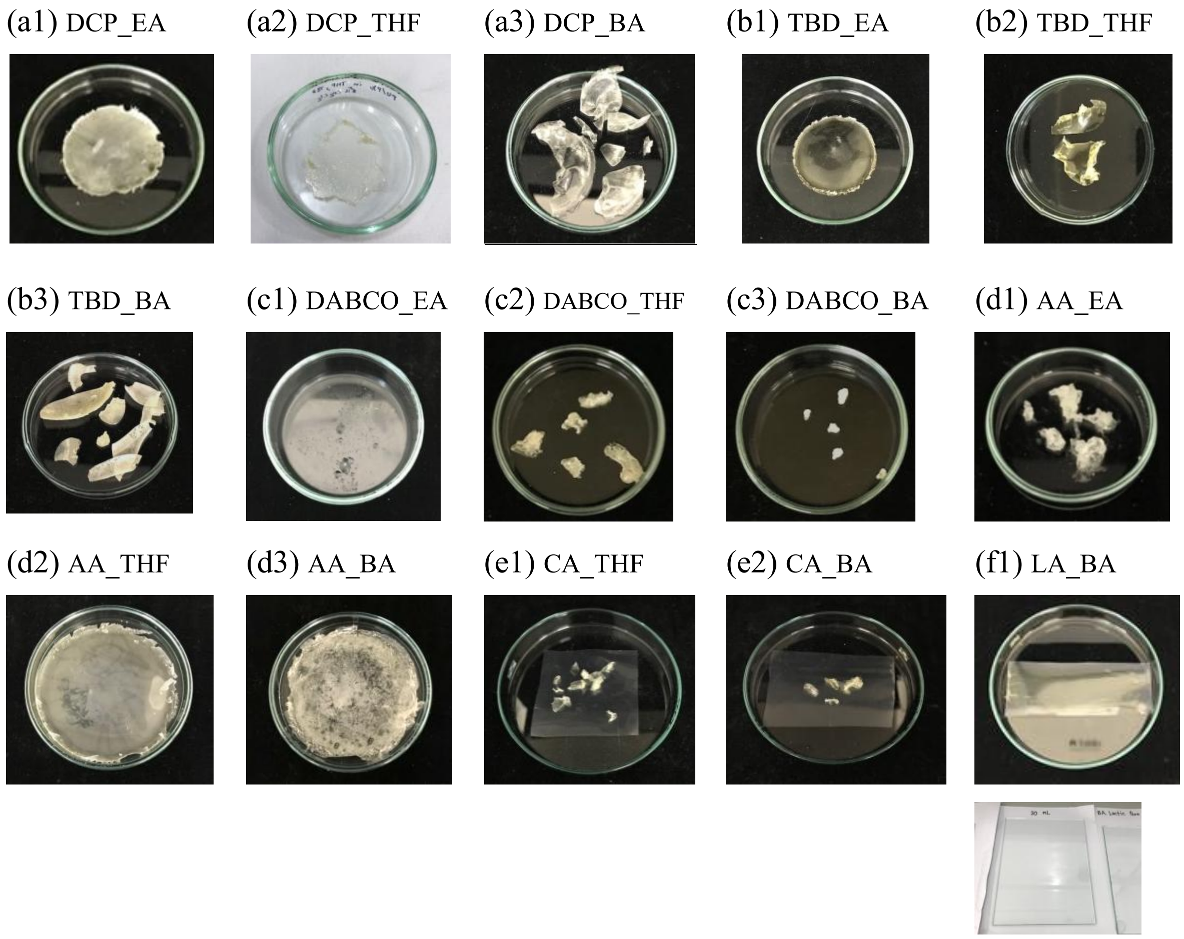

Figure S1. Physical appearance of obtained polyurethane thin films using different crosslinking agants including dicumyl peroxide (DCP), 1,5,7-triazabicyclo [4.4.0] dec-5-ene (TBD), 1,4-diazabicyclo [2.2.2] octane (DABCO), acetic acid (AA), citric acid (CA), and lactic acid (LA) and polar solvent including ethyl acetate (EA), tetrahydrofuran (THF), and butyl acetate (BA).

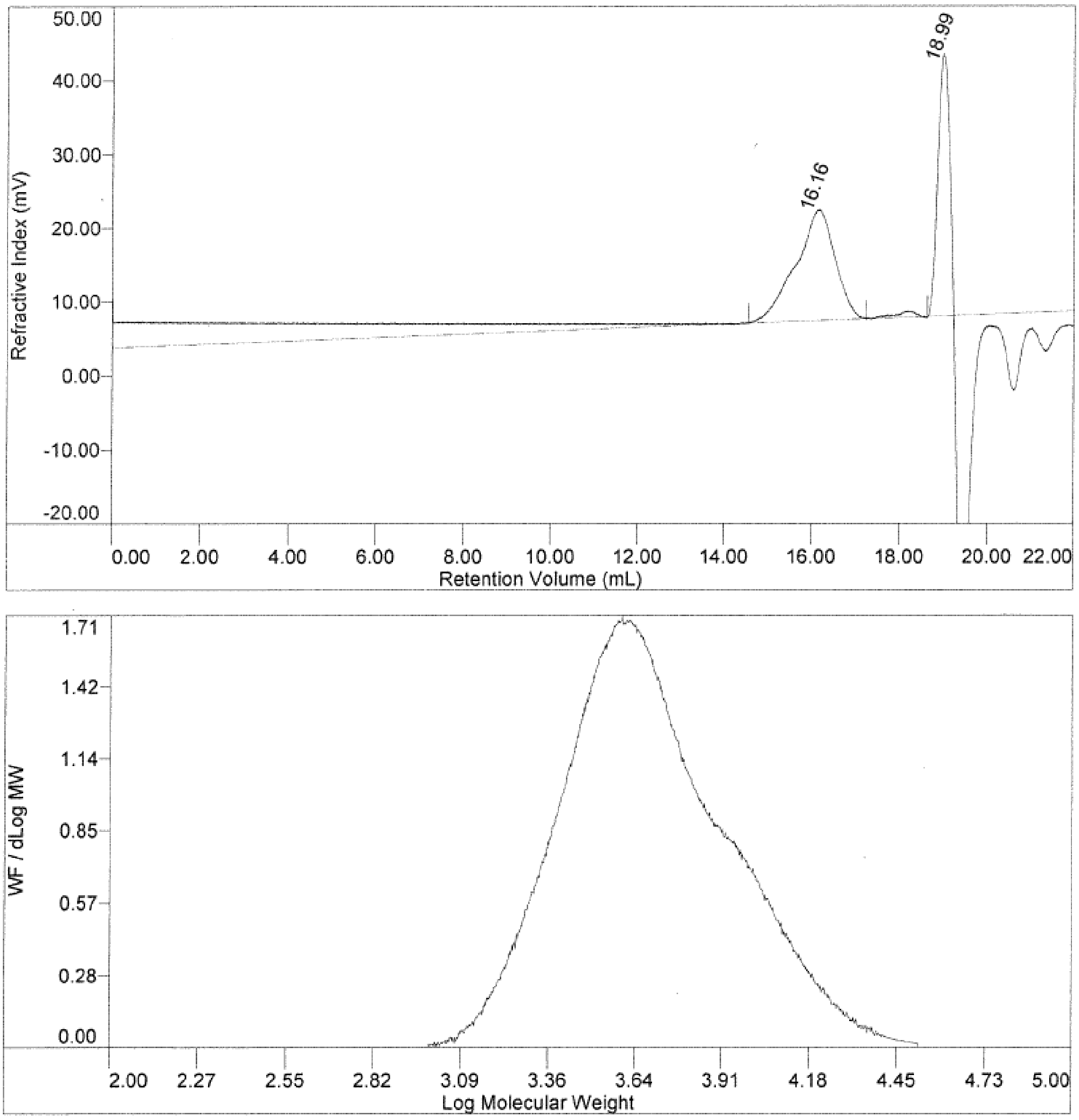

Sample	Mn	Mw	Mz	Mw/Mn
05-07-2018_11;38;00_PU_01.vdt	4,085	5,787	8,556	1.416

Figure S2. GPC chromatograms of PrePU.

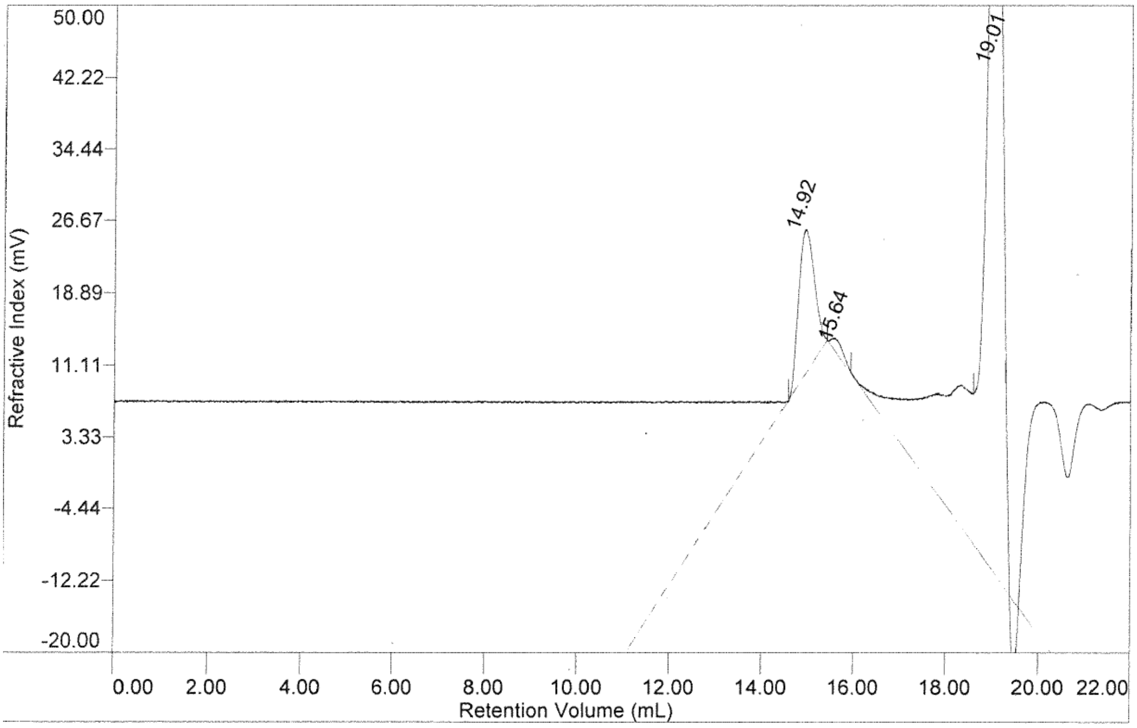

Peak	Mn	Mw	Mz	Mw/Mn
1	19,575	20,364	21,134	1.040
2	7,830	7,999	8,166	1.022

Figure S3. GPC chromatograms of Pluronic F-127.

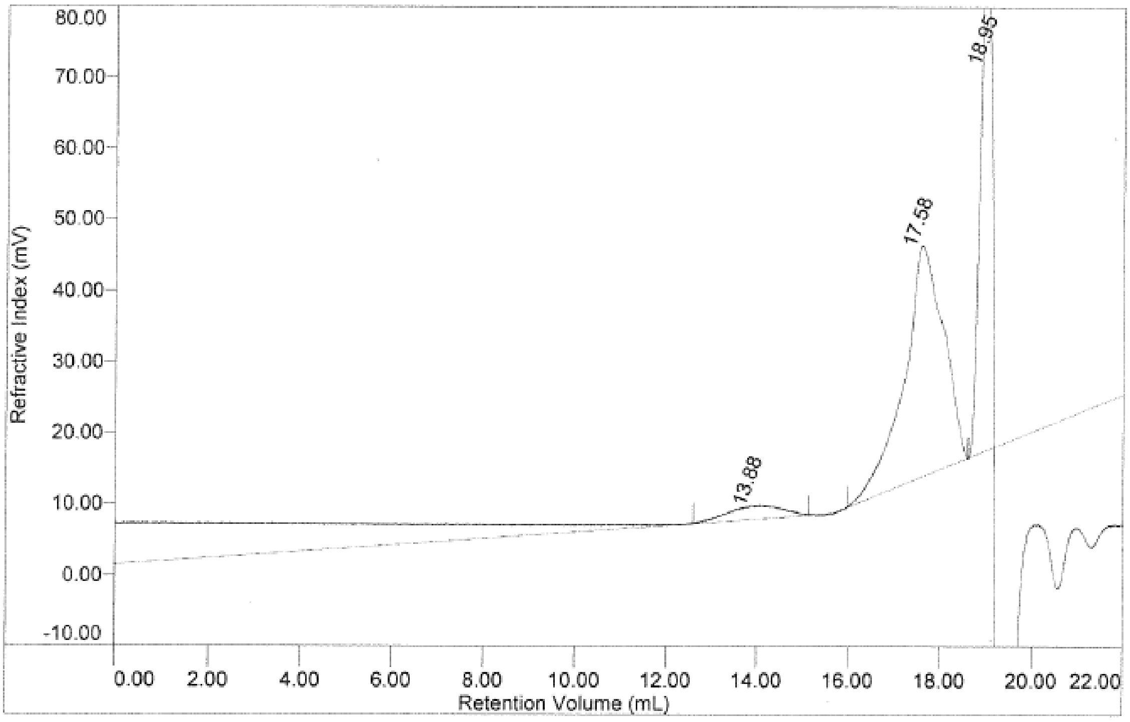

Peak	Mn	Mw	Mz	Mw/Mn
1	59,093	95,195	149,056	1.611
2	500	715	1,122	1.430

Figure S4. GPC chromatograms of the AA_BA copolymer.

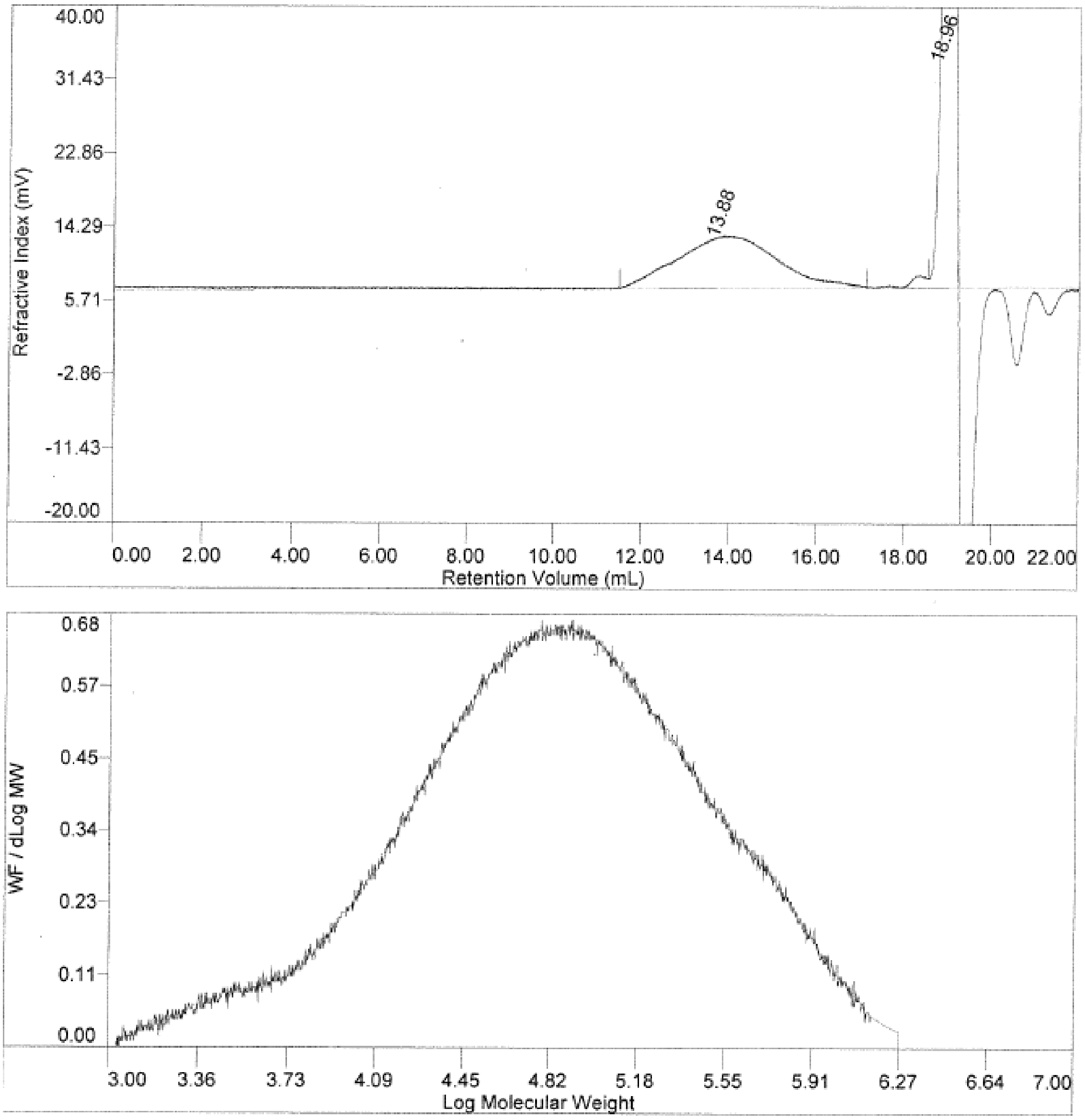

Sample	Mn	Mw	Mz	Mw/Mn
27-09-2018_10;54;11_PU_Plu_EA_acetic_acid_01.vdt	24,710	160,801	506,687	6.507

Figure S5. GPC chromatograms of the AA_EA copolymer.

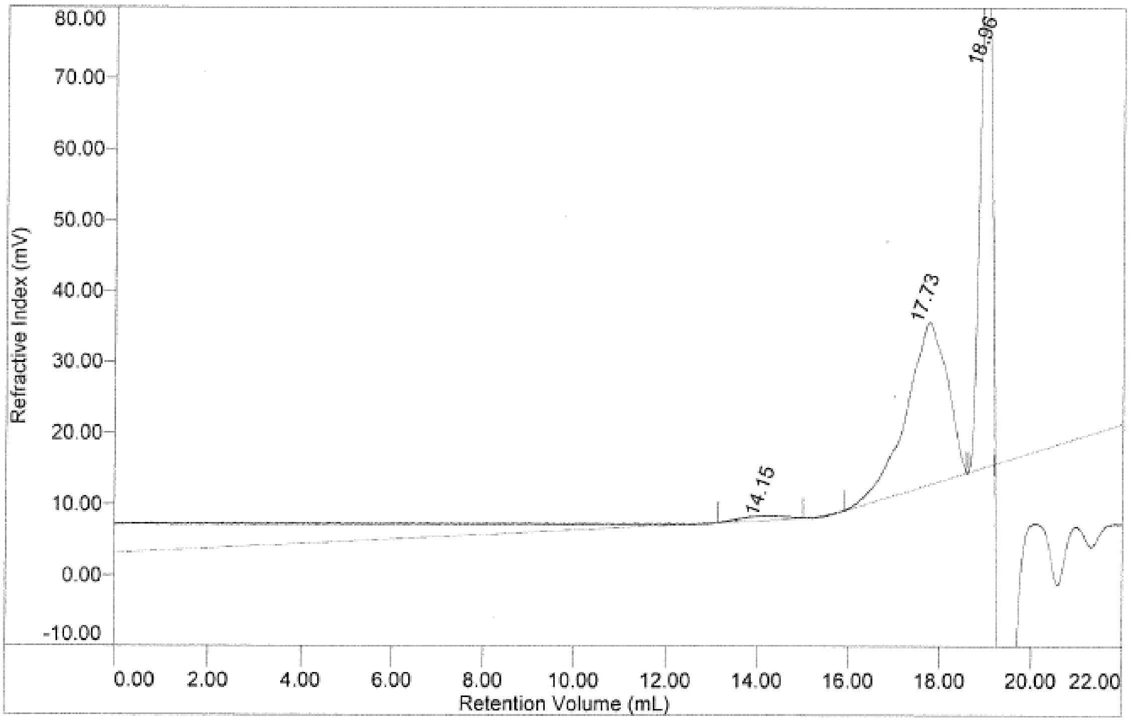

Peak	Mn	Mw	Mz	Mw/Mn
1	52,882	72,313	96,604	1.367
2	483	700	1,149	1.449

Figure S6. GPC chromatograms of the AA_THF copolymer.

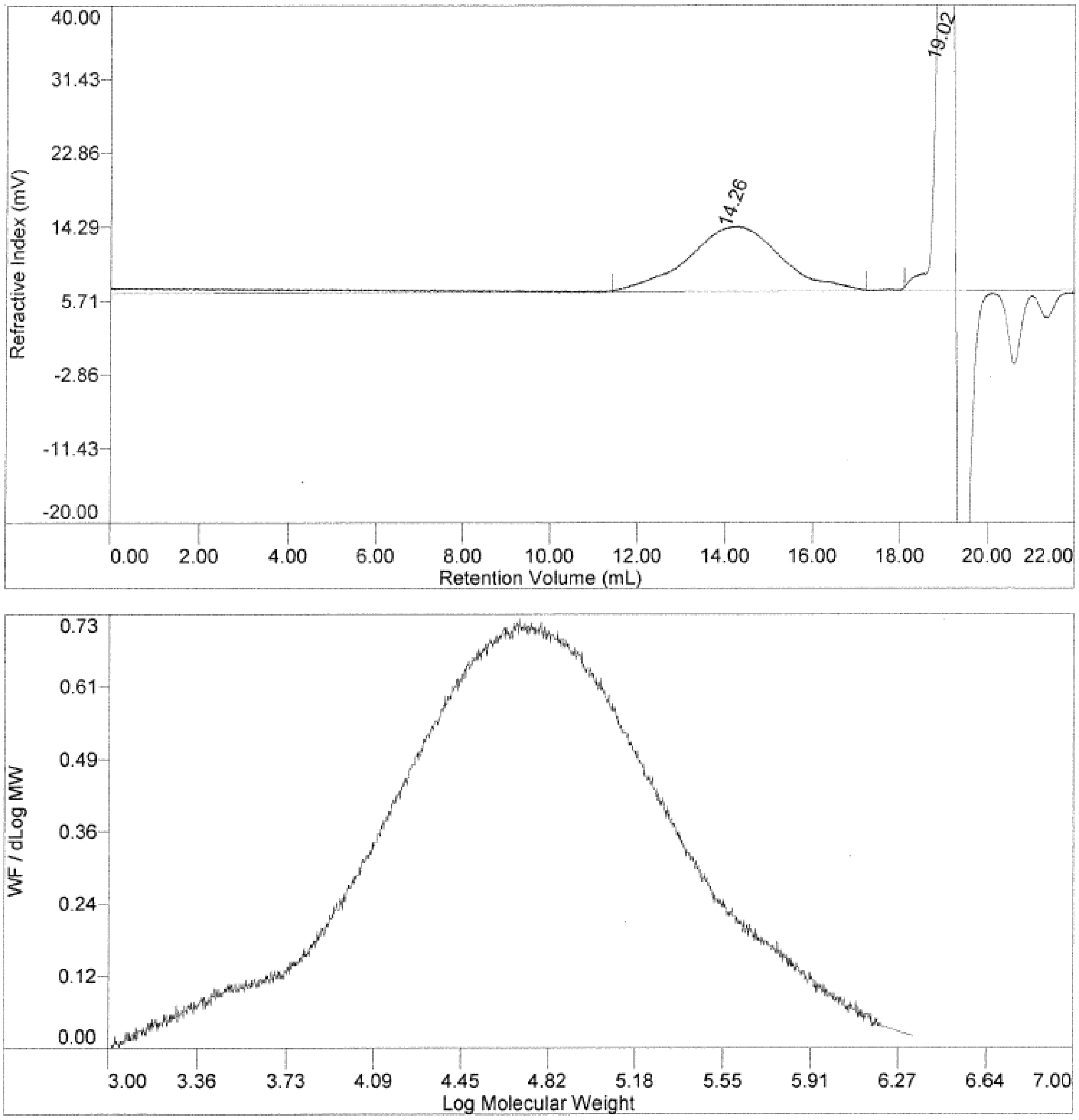

Sample	Mn	Mw	Mz	Mw/Mn
08-10-2018_10;38;46_PU_Plu_BA_D_01.vdt	21,783	131,607	522,000	6.042

Figure S7. GPC chromatograms of the DABCO_BA copolymer.

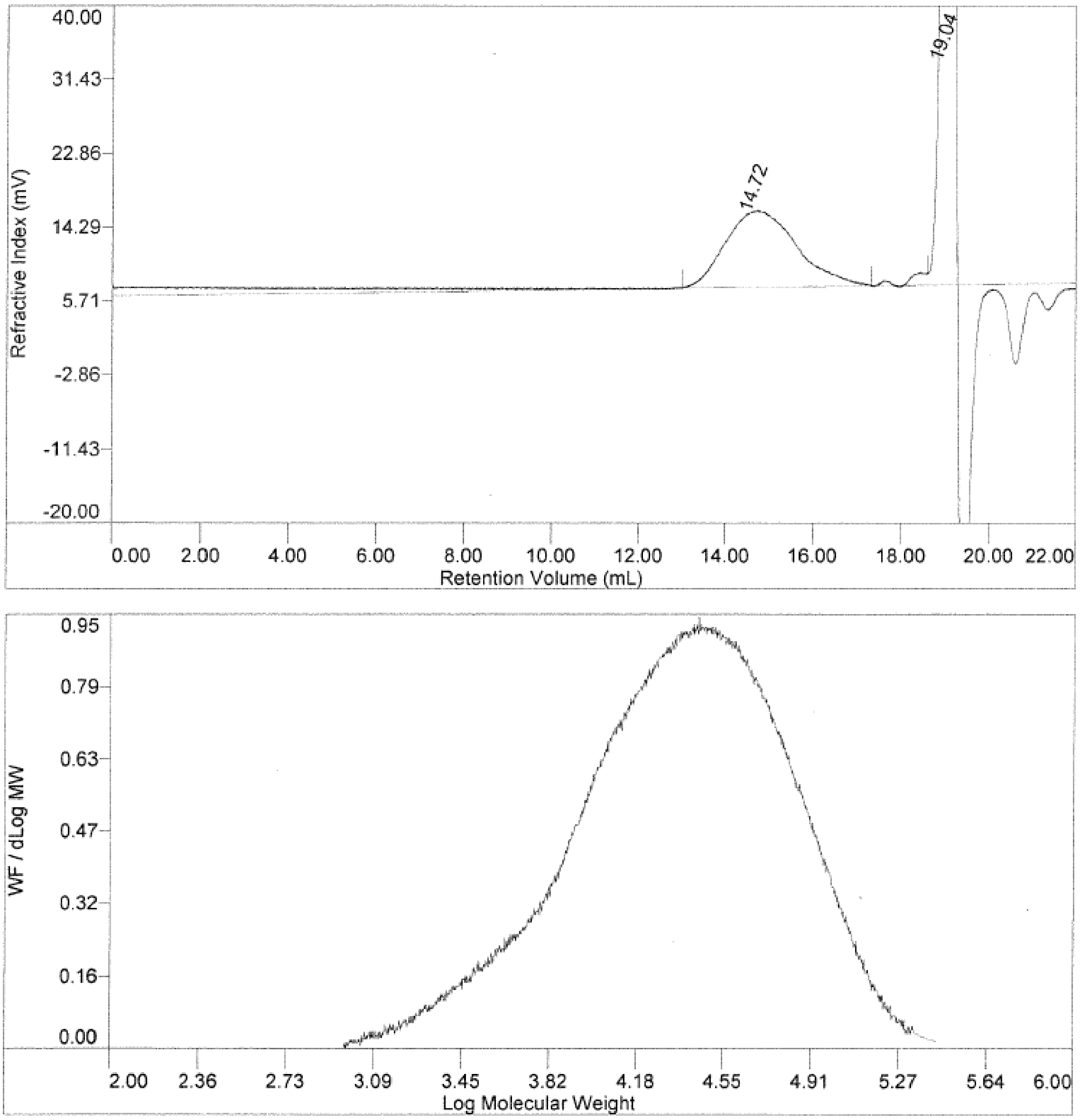

Sample	Mn	Mw	Mz	Mw/Mn
08-10-2018_11;27;52_PU_Plu_EA_D_01.vdt	13,471	35,624	68,440	2.644

Figure S8. GPC chromatograms (RI detector response vs. retention volume) of the DABCO_EA copolymer.

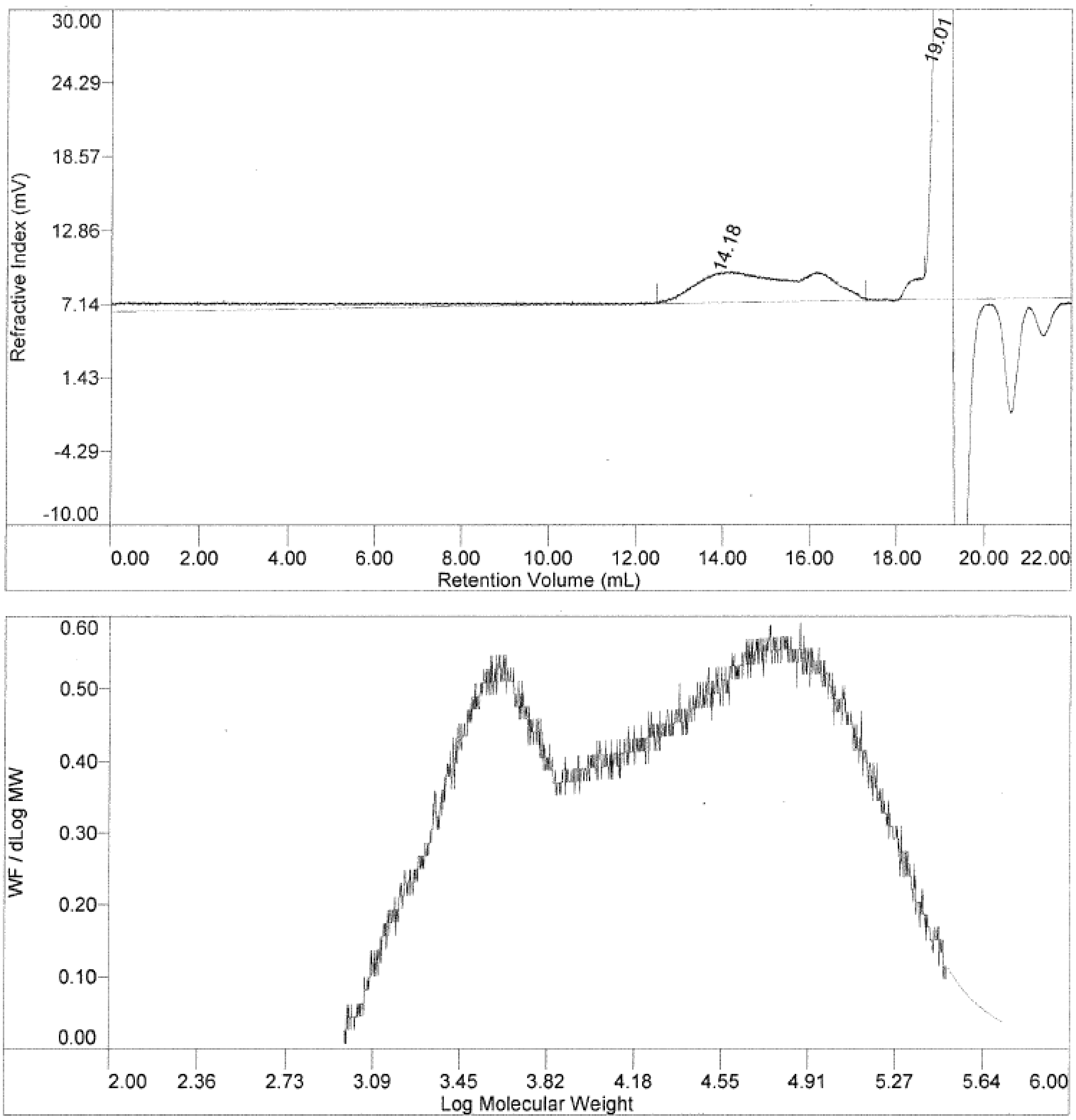

Sample	Mn	Mw	Mz	Mw/Mn
08-10-2018_11;52;24_PU_Plu_THF_D_01.vdt	8,151	53,108	153,420	6.516

Figure S9. GPC chromatograms (RI detector response vs. retention volume) of the DABCO_THF copolymer.

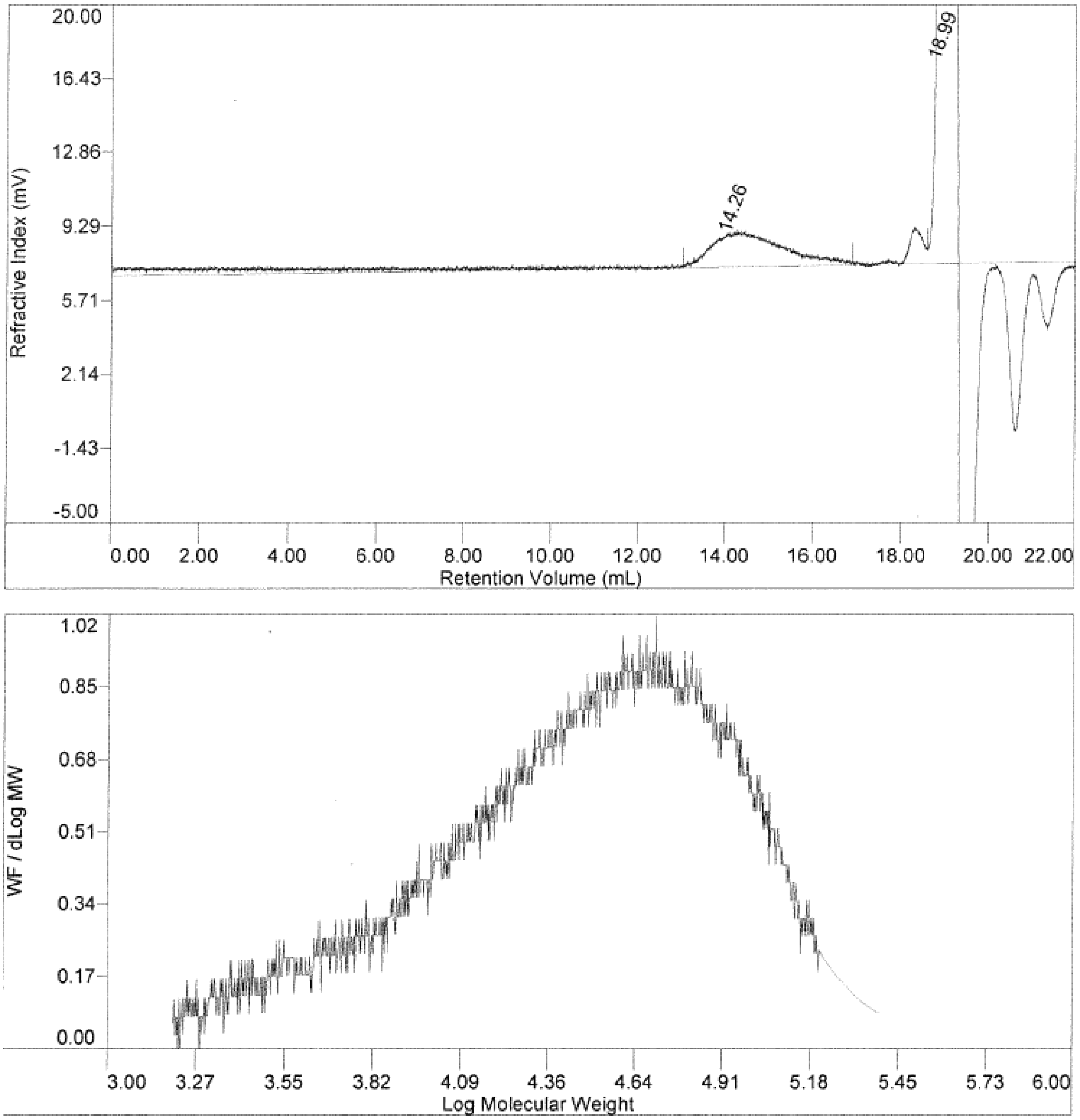

Sample	Mn	Mw	Mz	Mw/Mn
19-10-2018_12;04;14_PU_Plu_THF_DCP_01.vdt	15,657	46,978	86,528	3.000

Figure S10. GPC chromatograms (RI detector response vs. retention volume) of the DCP_THF copolymer.

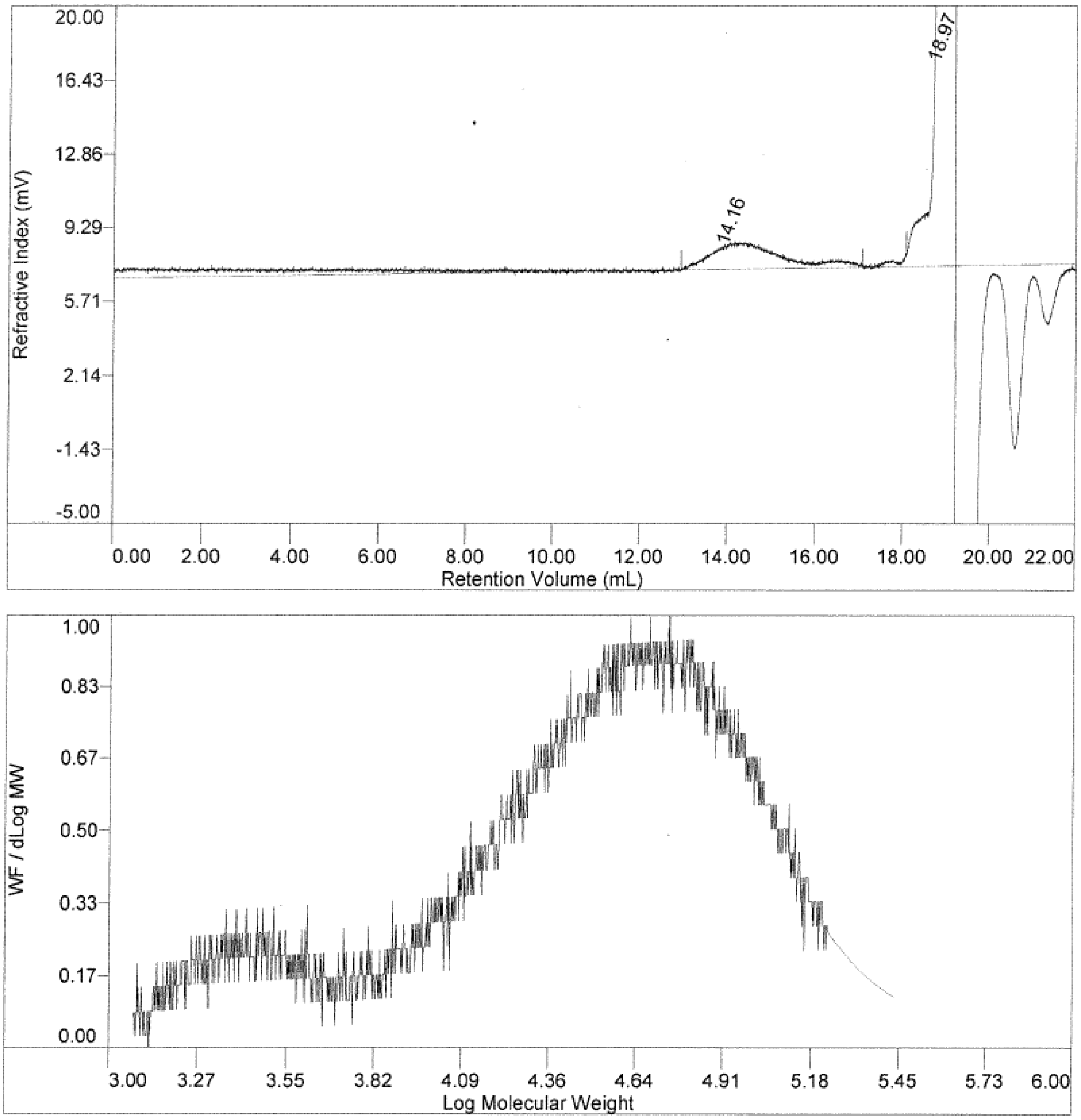

Sample	Mn	Mw	Mz	Mw/Mn
05-07-2018_12;27;06_BA_TBD_01.vdt	12,904	51,945	99,611	4.025

Figure S11. GPC chromatograms (RI detector response vs. retention volume) of the TBD_BA copolymer.

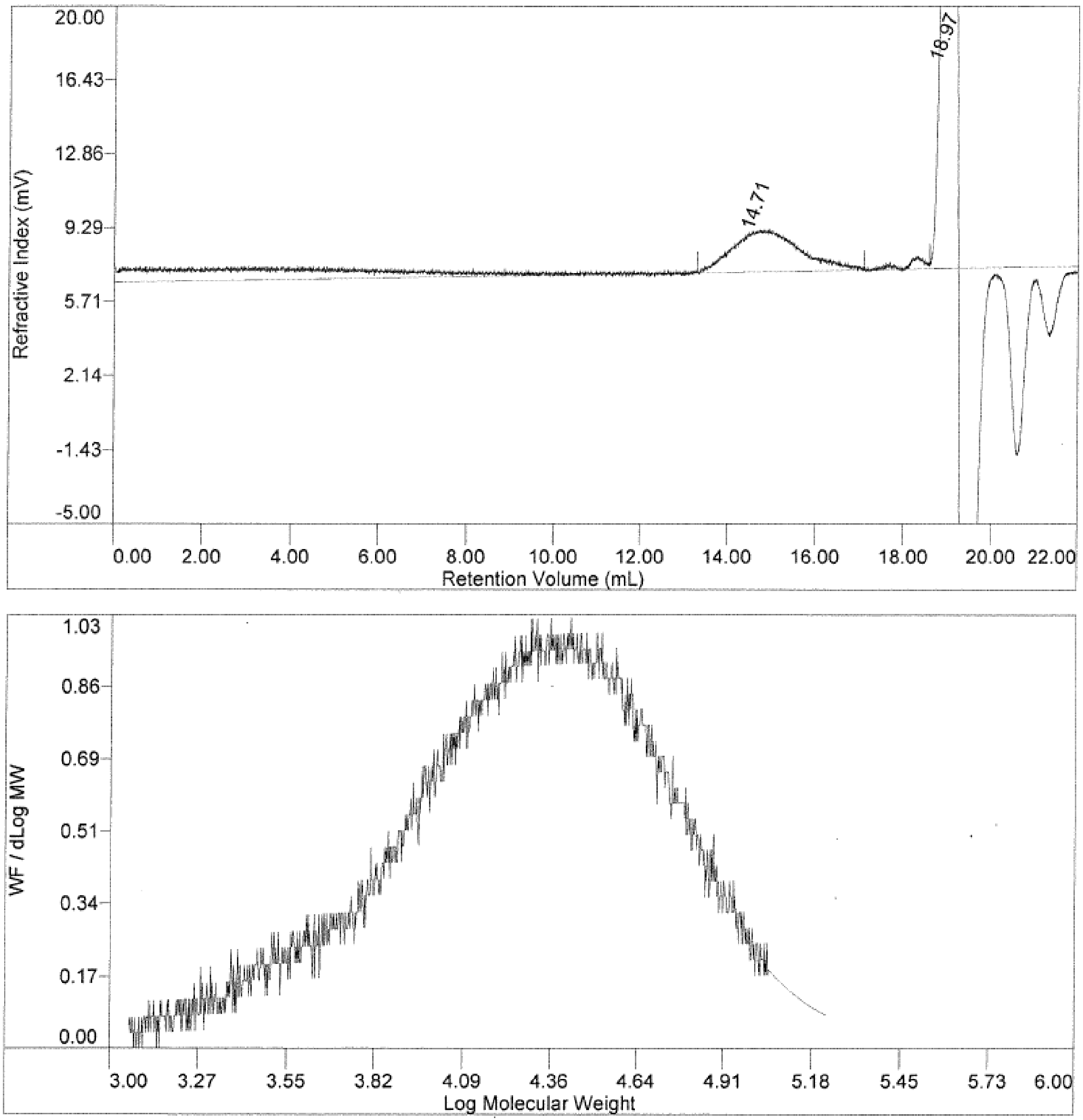

Sample	Mn	Mw	Mz	Mw/Mn
05-07-2018_12;51;38_EA_TBD_01.vdt	11,706	29,748	55,242	2.541

Figure S12. GPC chromatograms (RI detector response vs. retention volume) of the TBD_EA copolymer.

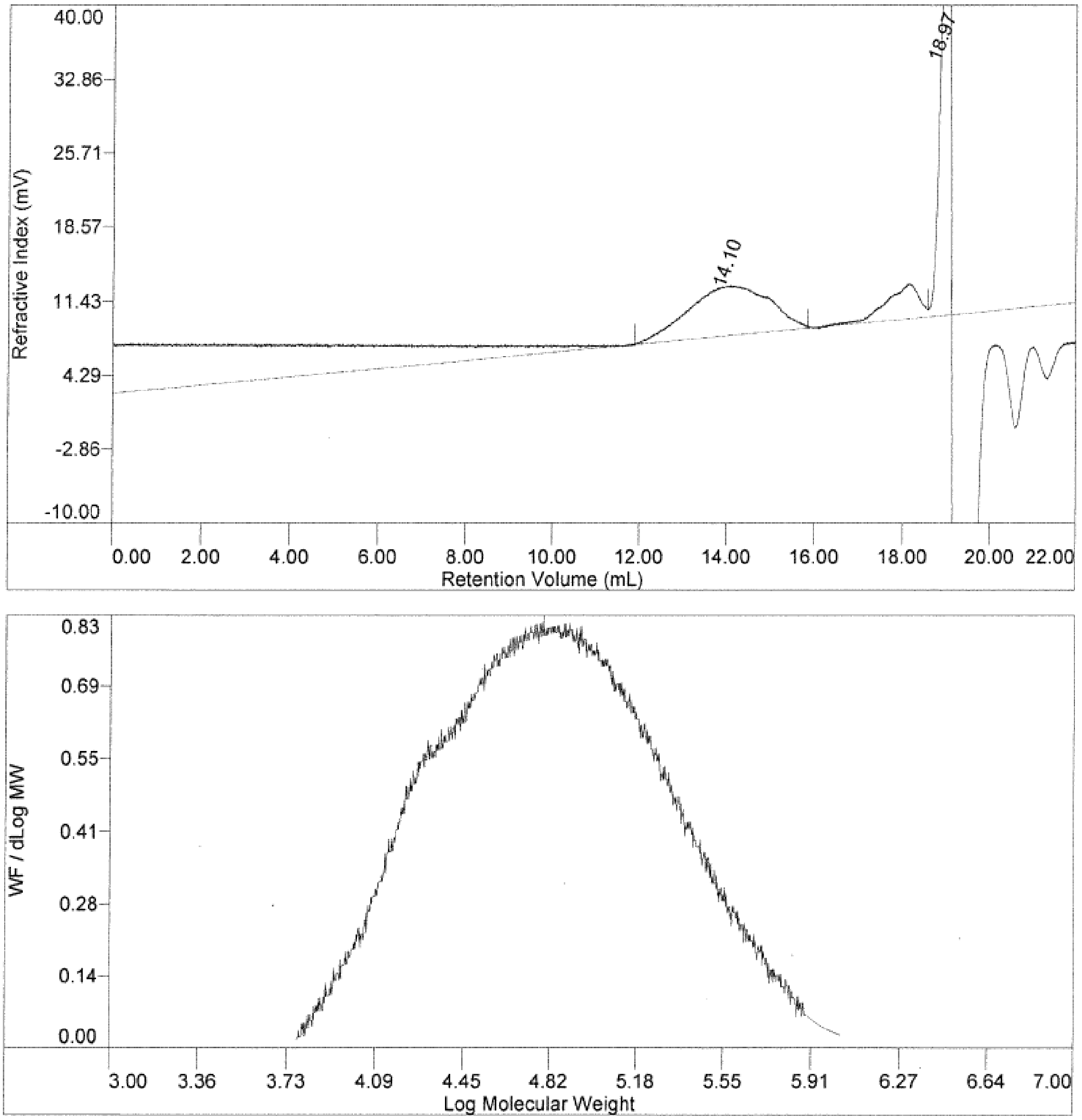

Sample	Mn	Mw	Mz	Mw/Mn
05-07-2018_14;05;16_THF_TBD_01.vdt	41,119	115,449	277,795	2.808

Figure S13. GPC chromatograms (RI detector response vs. retention volume) of the TBD_THF copolymer.

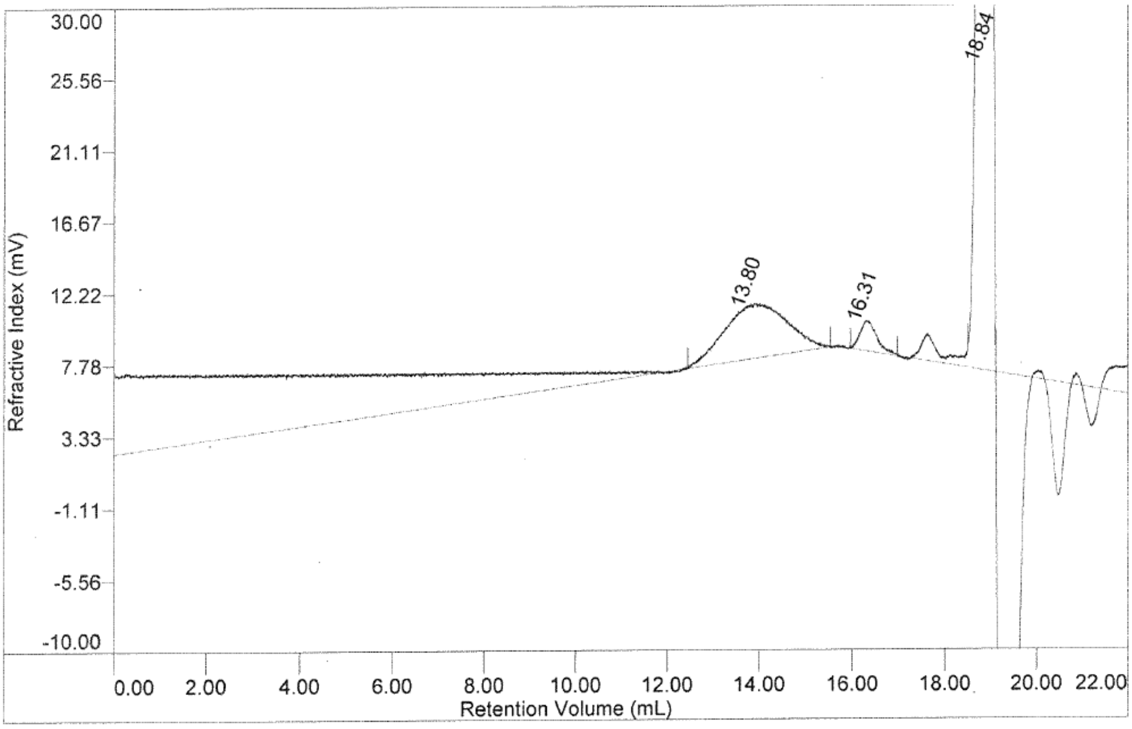

Peak	Mn	Mw	Mz	Mw/Mn
1	48,915	90,781	153,635	1.856
2	2,371	2,526	2,668	1.065

Figure S14. GPC chromatograms (RI detector response vs. retention volume) of the LA_BA copolymer.

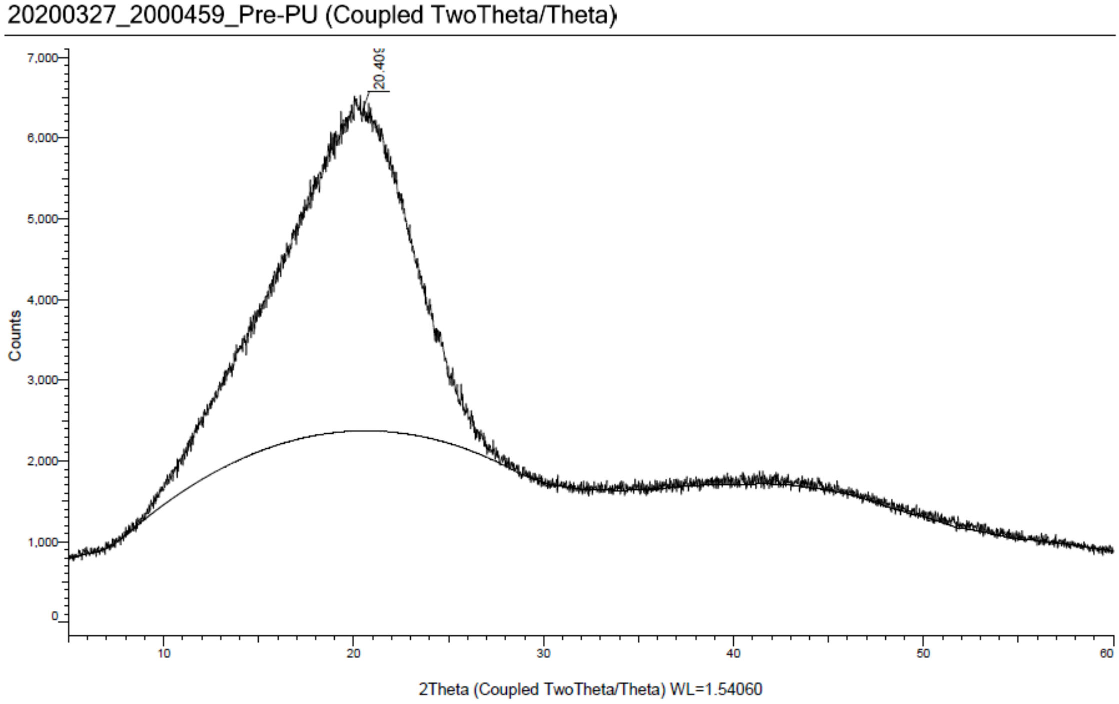

Figure S15. X-ray diffraction (XRD) patterns of PrePU.

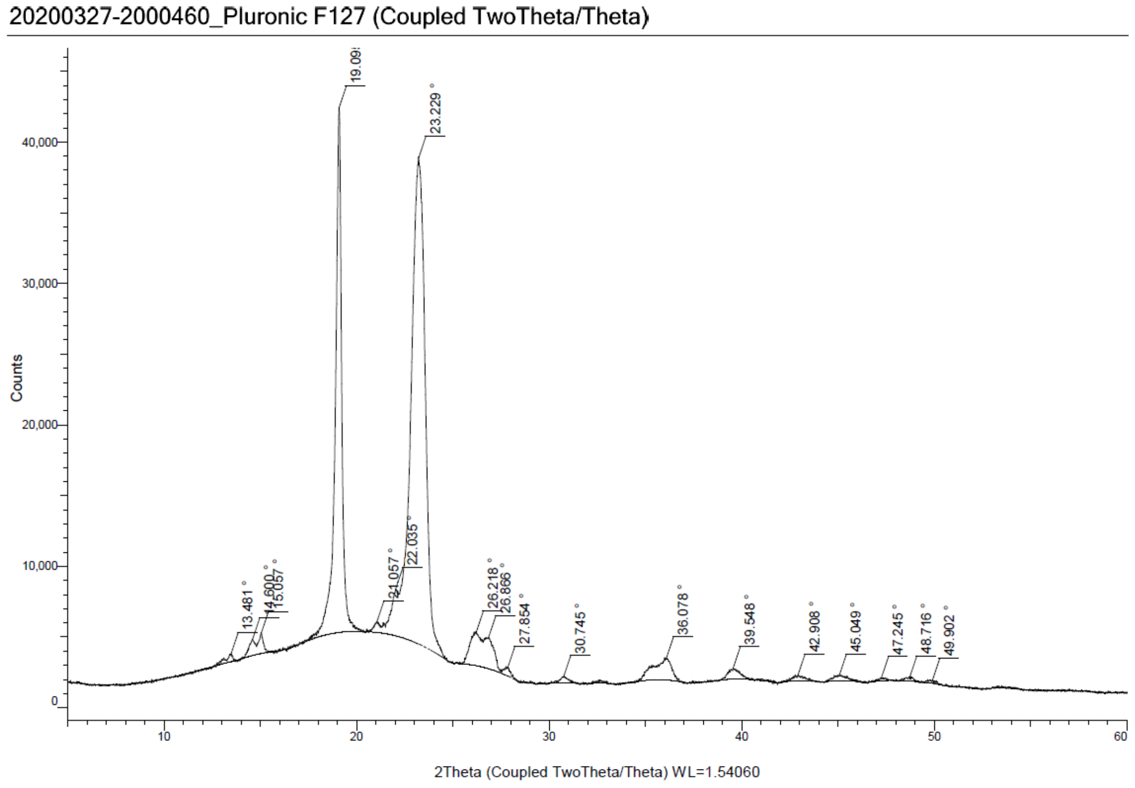

Figure S16. X-ray diffraction (XRD) patterns of Pluronic F-127.

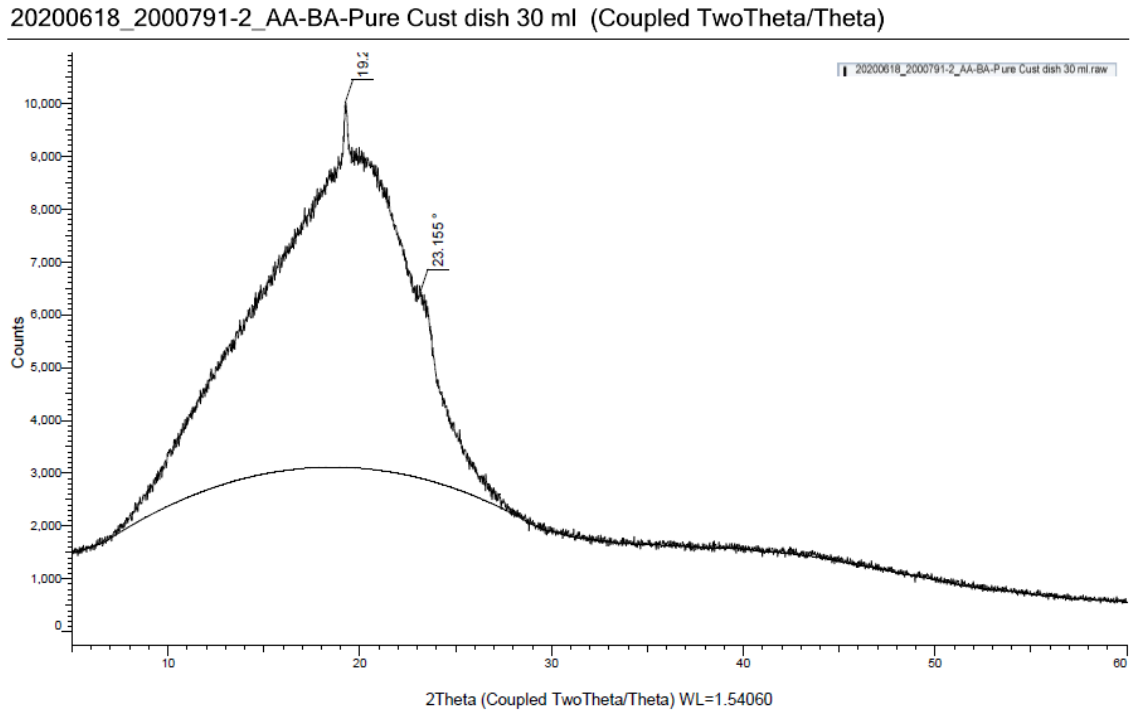

Figure S17. X-ray diffraction (XRD) patterns of the AA_BA copolymer.

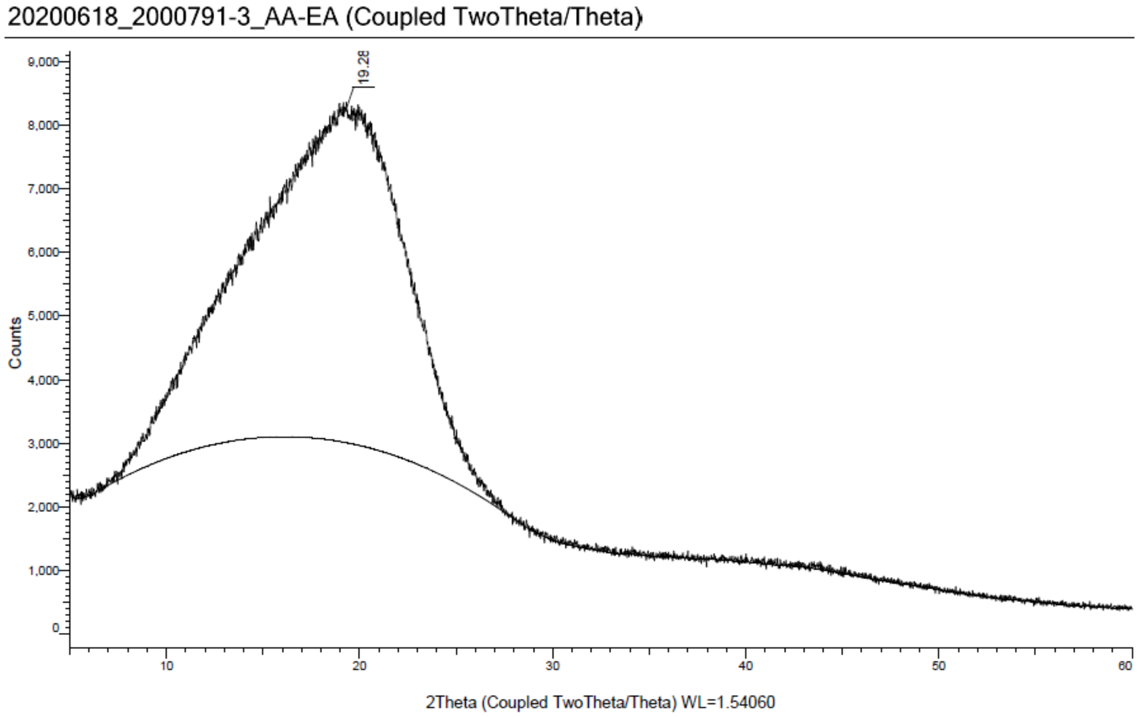

Figure S18. X-ray diffraction (XRD) patterns of the AA_EA copolymer.

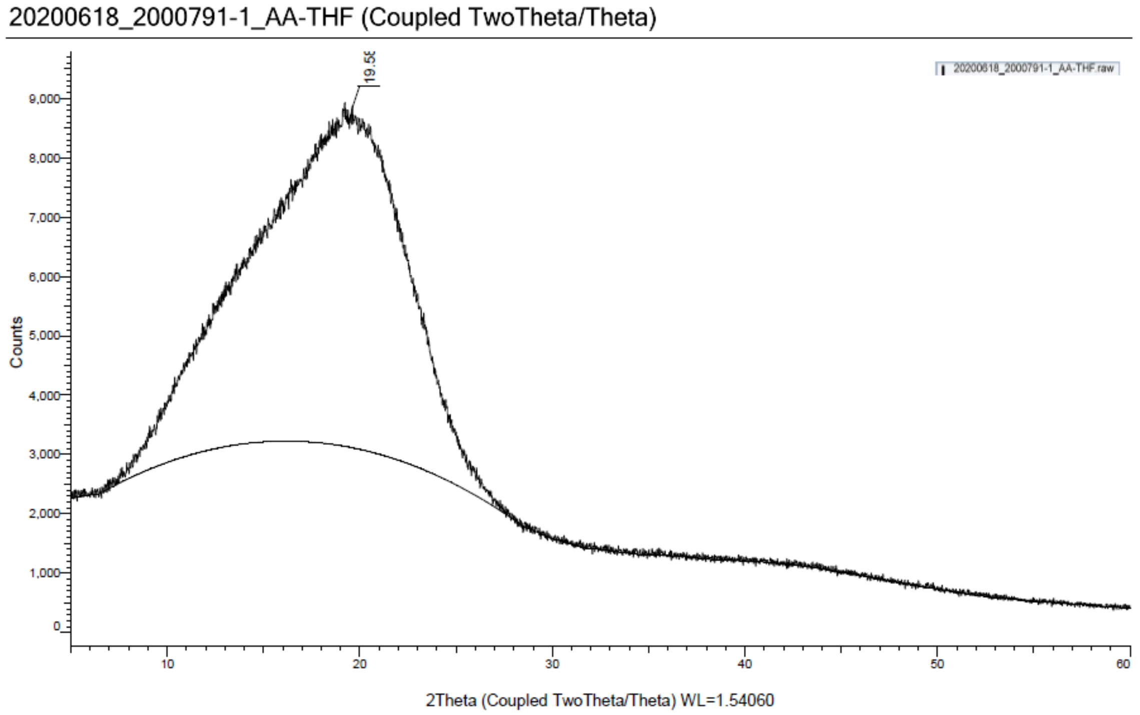

Figure S19. X-ray diffraction (XRD) patterns of the AA_THF copolymer.

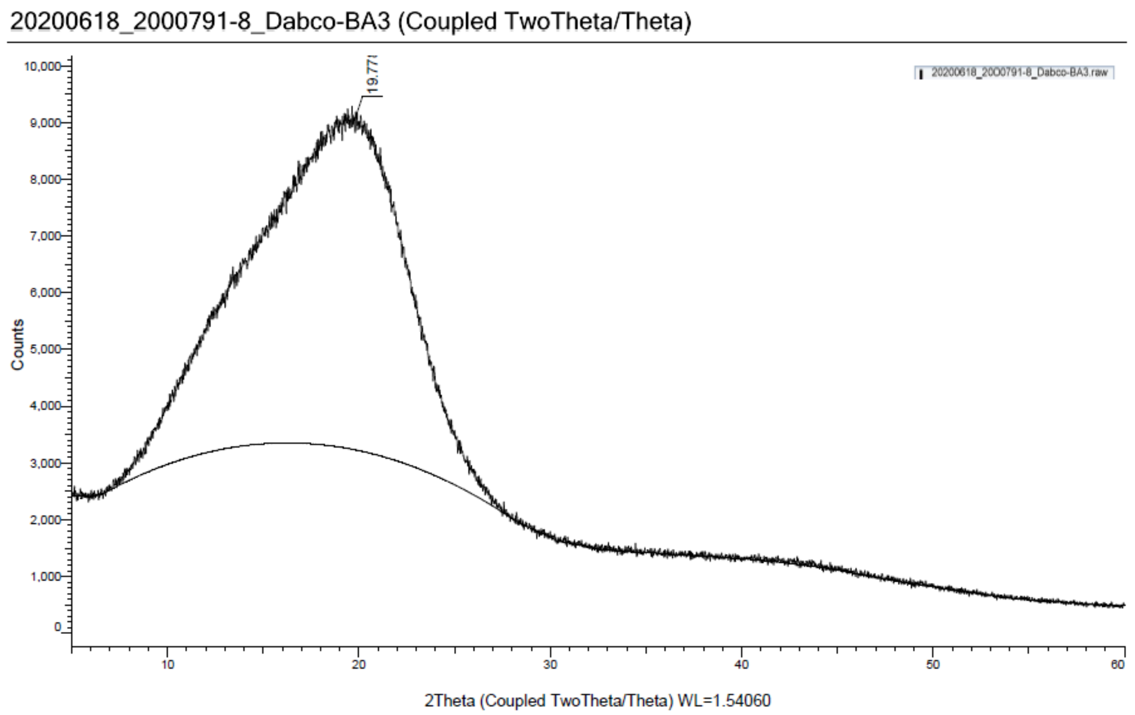

Figure S20. X-ray diffraction (XRD) patterns of the DABCO_BA copolymer.

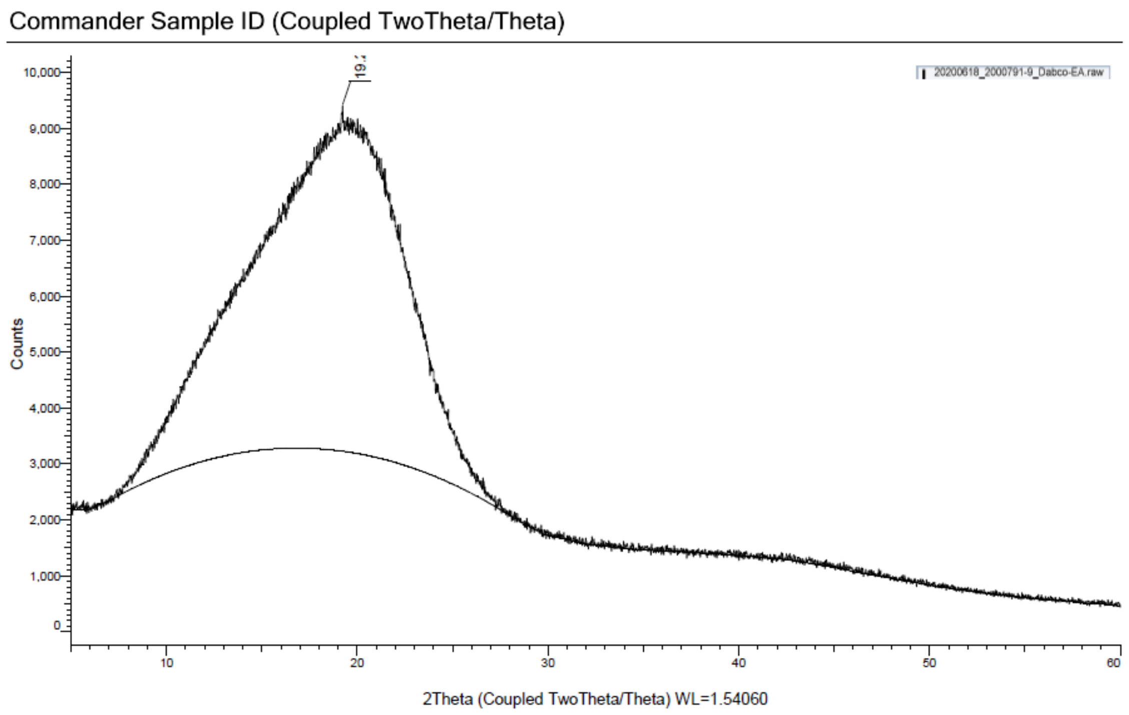

Figure S21. X-ray diffraction (XRD) patterns of the DABCO_EA copolymer.

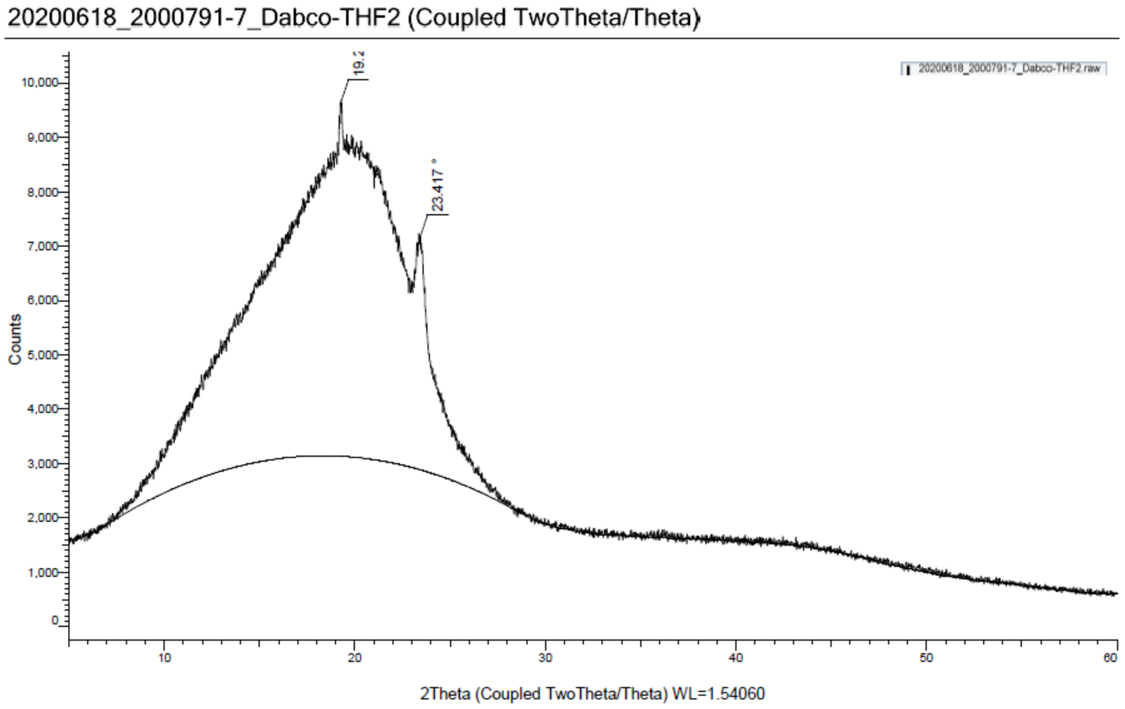

Figure S22. X-ray diffraction (XRD) patterns of the DABCO_THF copolymer.

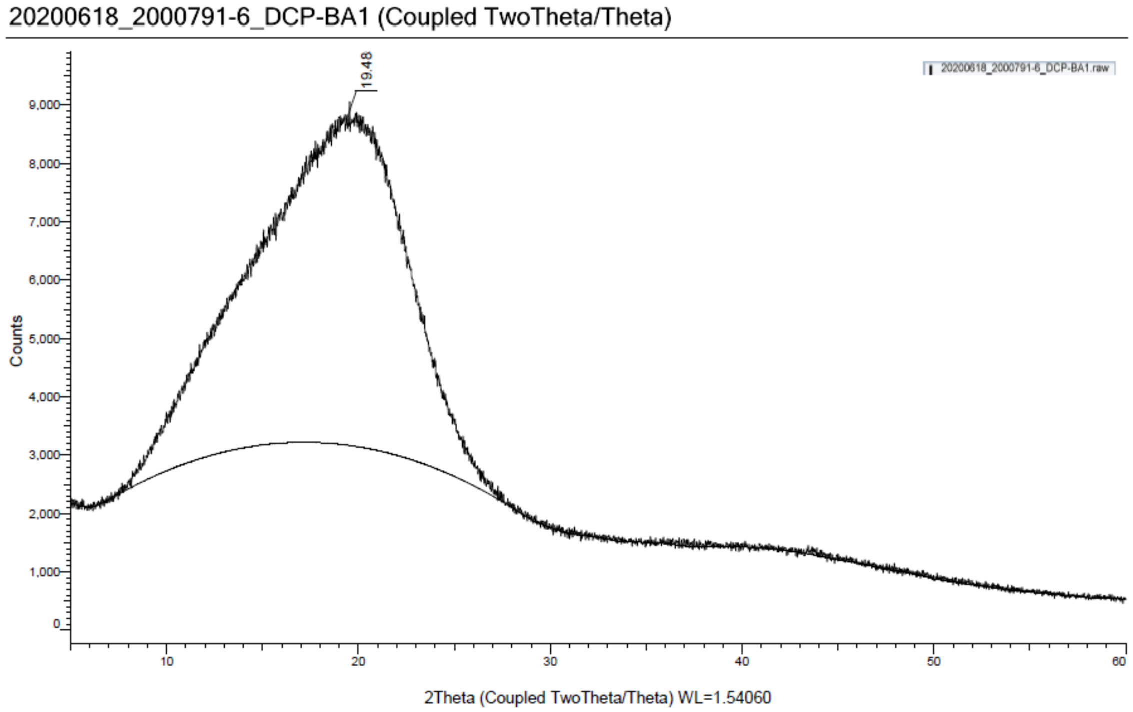

Figure S23. X-ray diffraction (XRD) patterns of the DCP_BA copolymer.

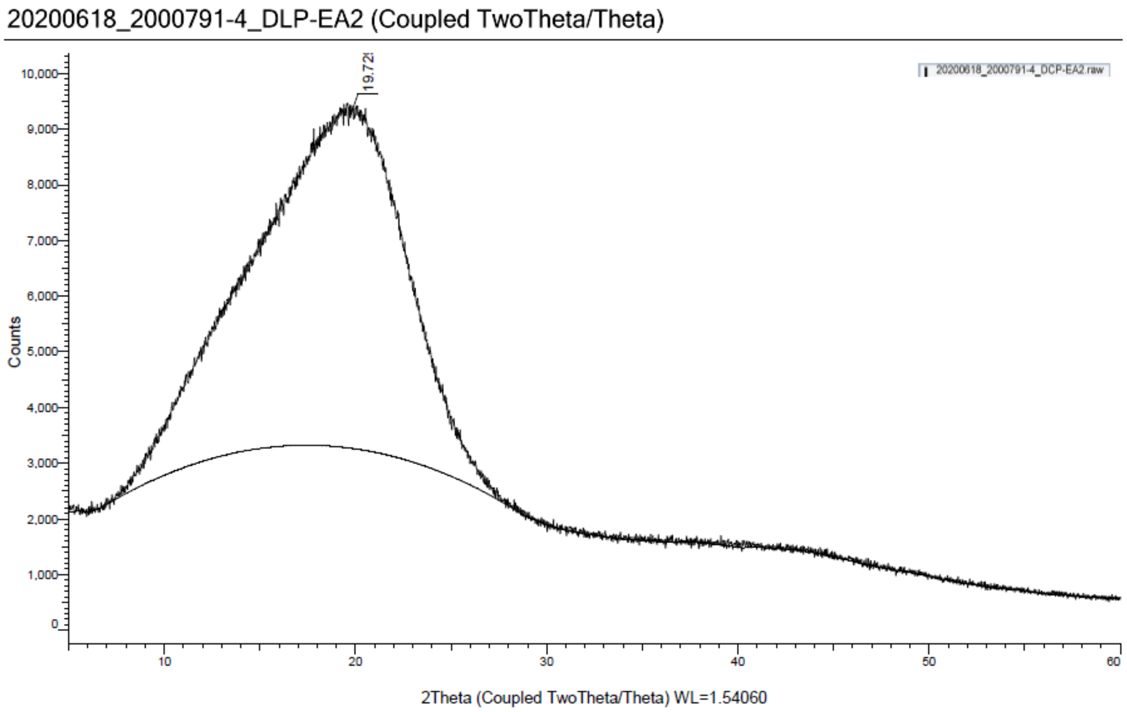

Figure S24. X-ray diffraction (XRD) patterns of the DCP_EA copolymer.

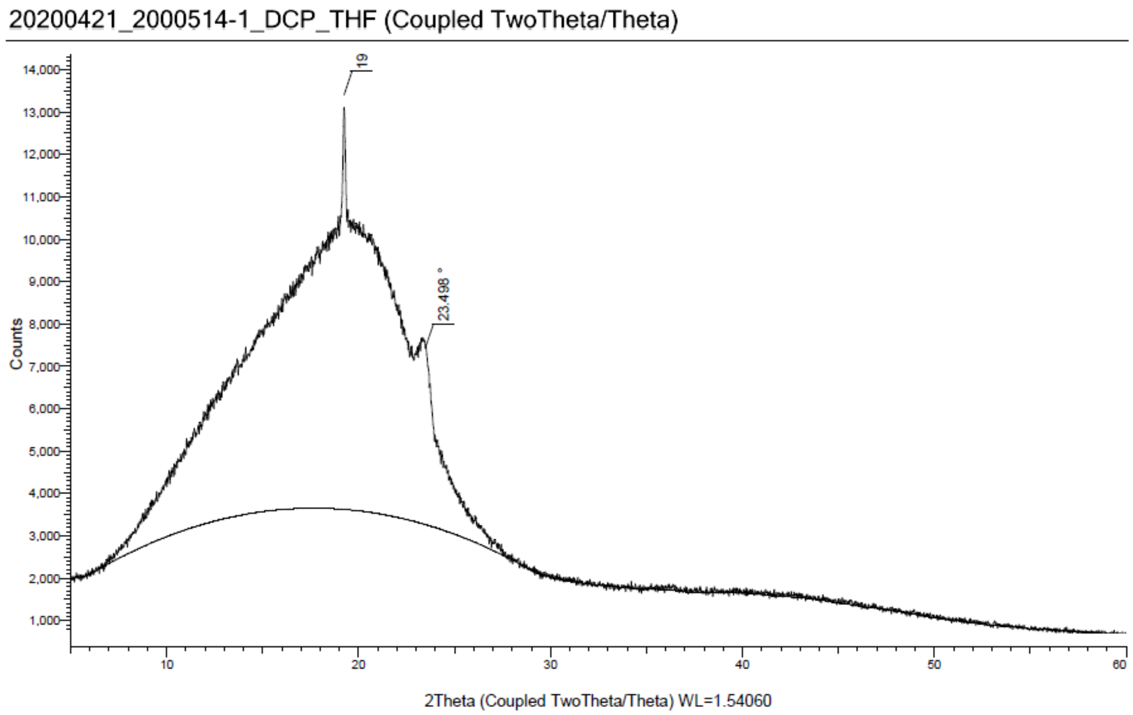

Figure S25. X-ray diffraction (XRD) patterns of the DCP_THF copolymer.

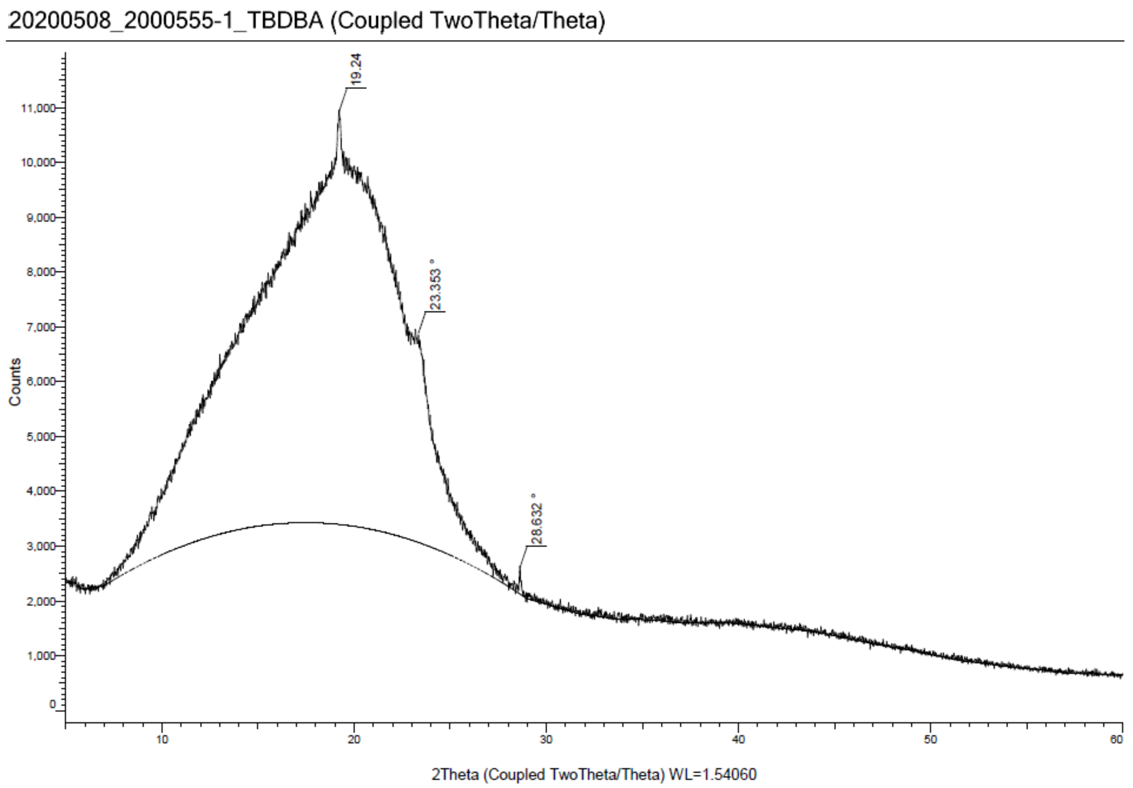

Figure S26. X-ray diffraction (XRD) patterns of the TBD_BA copolymer.

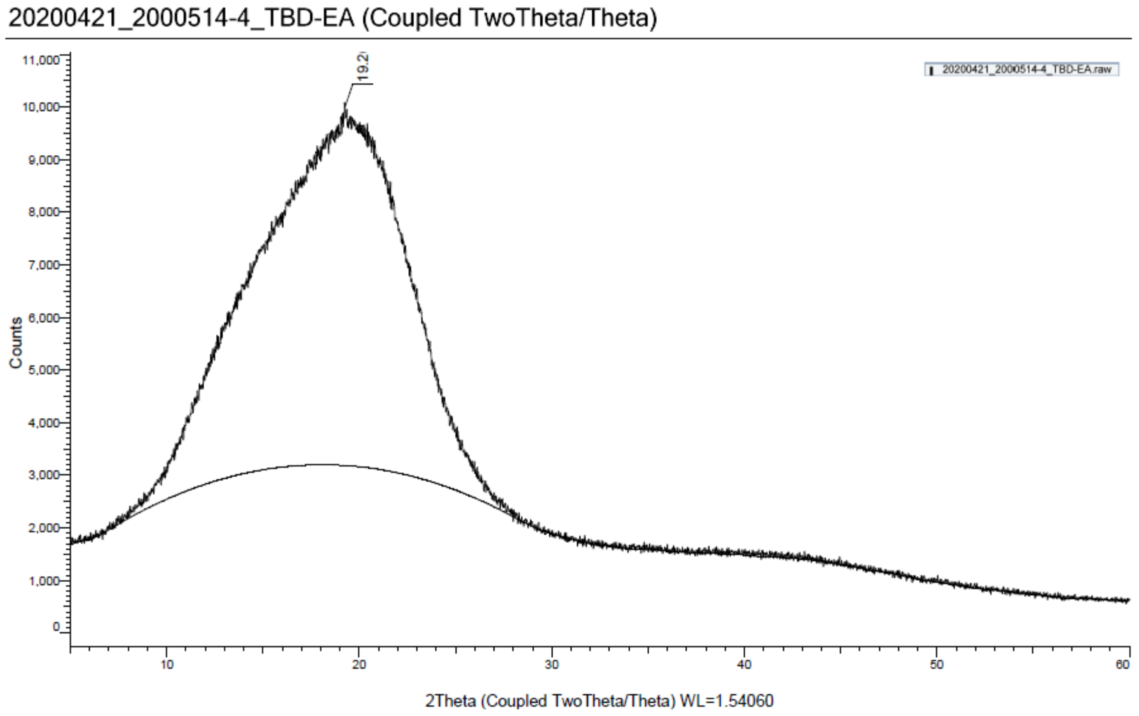

Figure S27. X-ray diffraction (XRD) patterns of the TBD_EA copolymer.

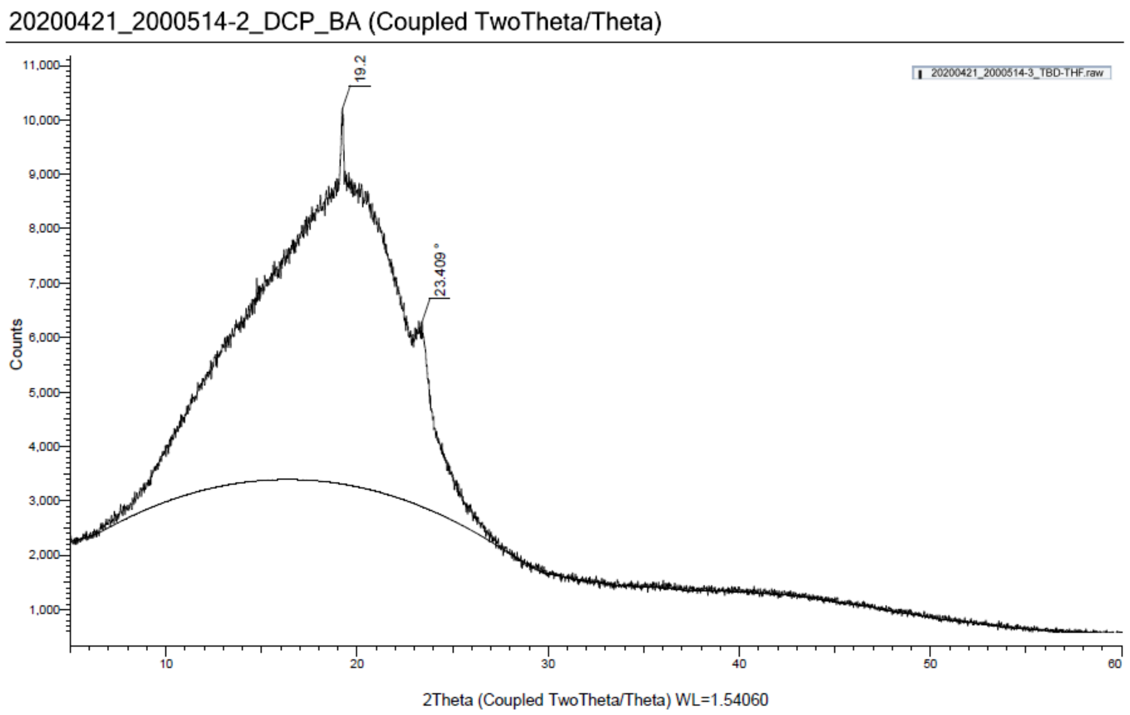

Figure S28. X-ray diffraction (XRD) patterns of the TBD_THF copolymer.

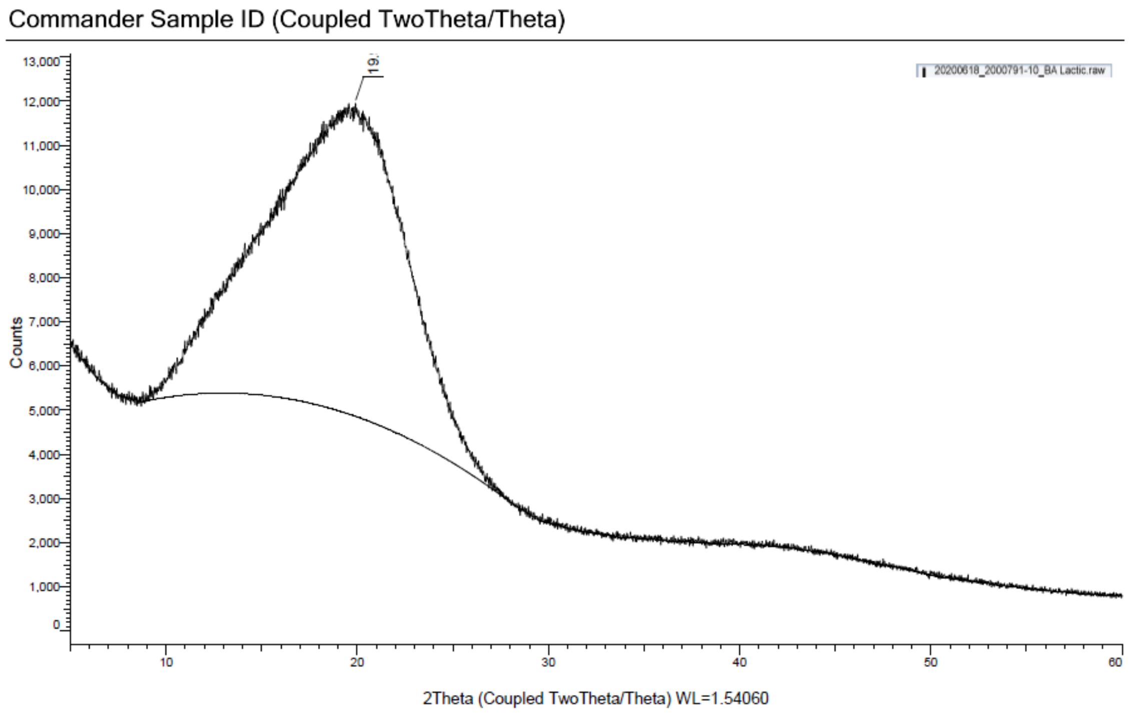

Figure S29. X-ray diffraction (XRD) patterns of the LA_BA copolymer.

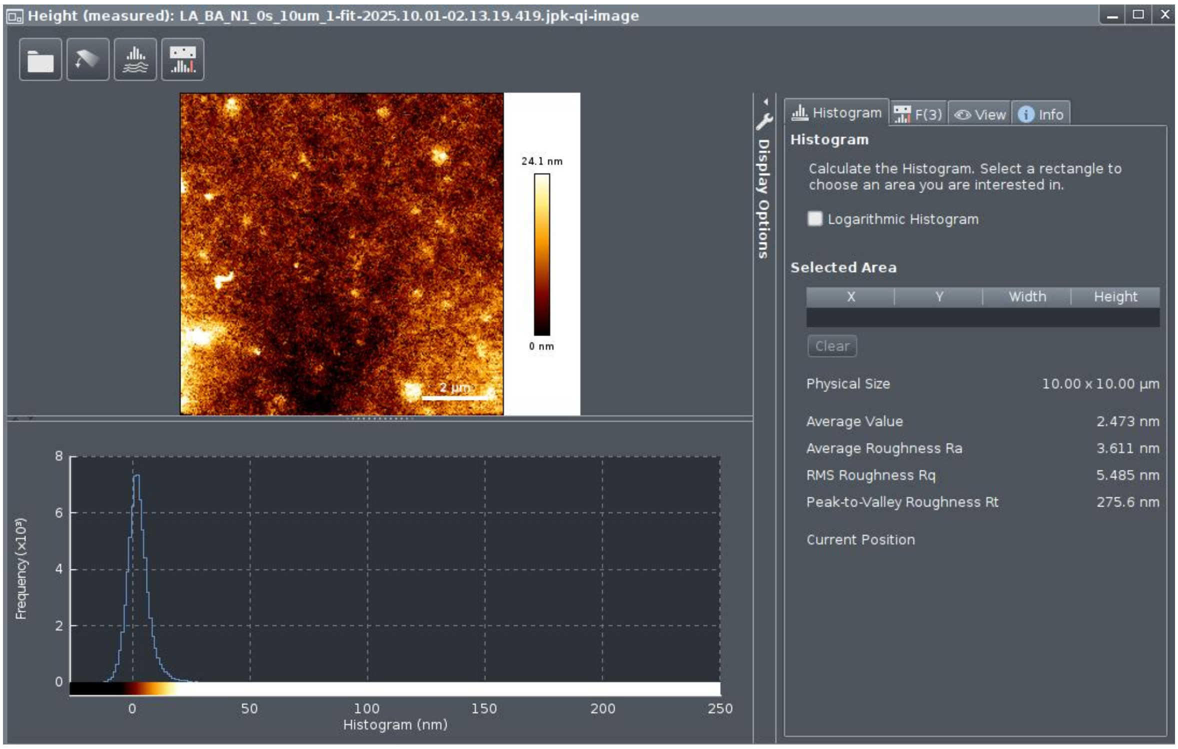

Figure S30. AFM analysis of the un-etched LA_BA film. Large-area (10 x 10 µm) height topography.

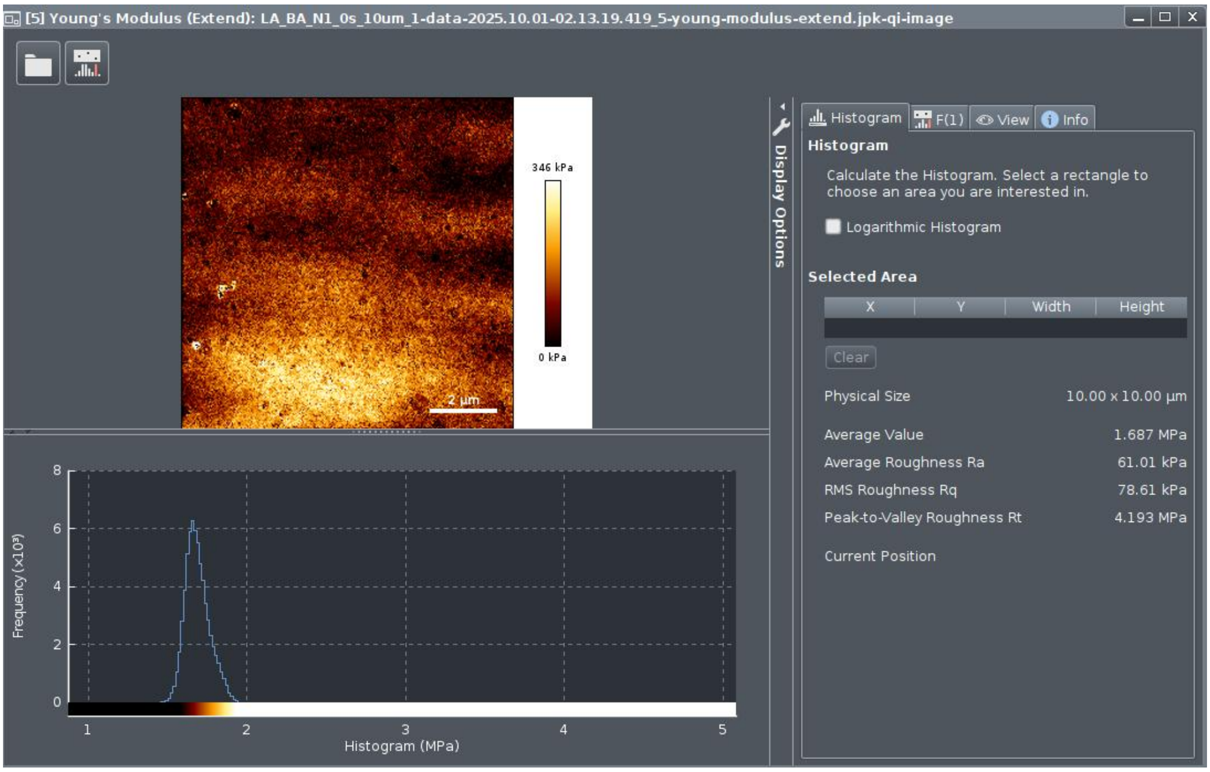

Figure S31. AFM analysis of the un-etched LA_BA film. Large-area (10 x 10 µm) young's modulus map.

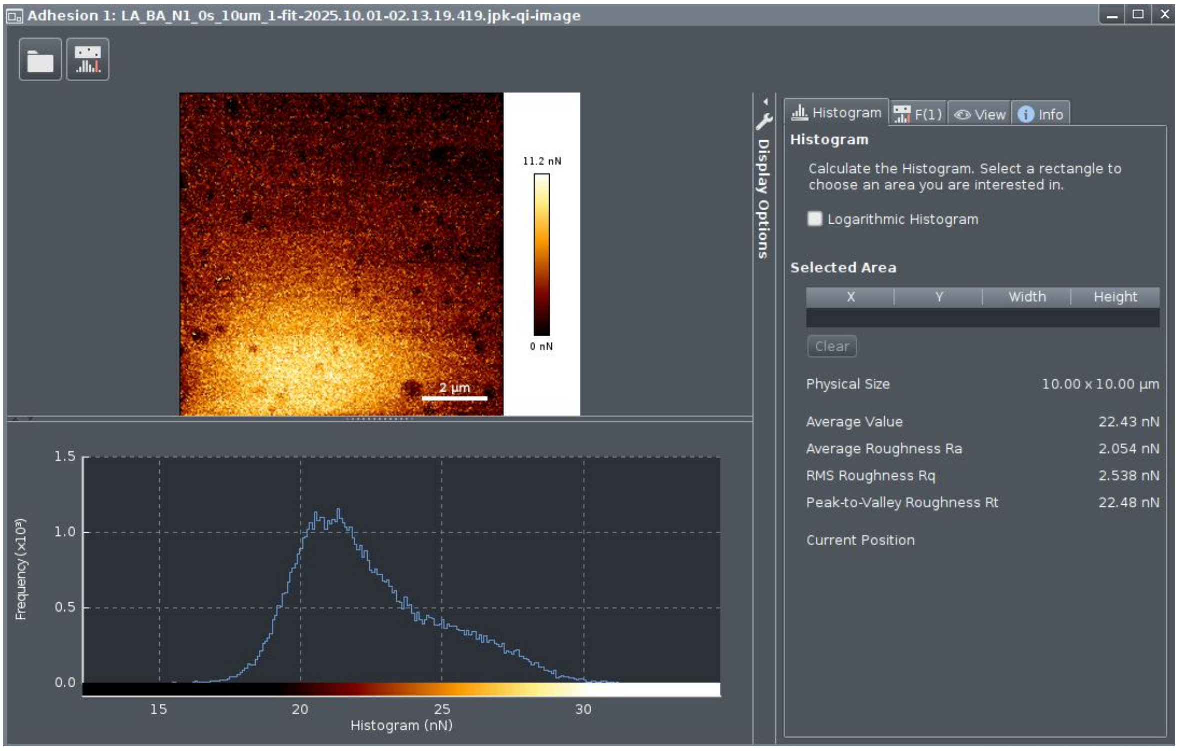

Figure S32. AFM analysis of the un-etched LA_BA film. Large-area (10 x 10 µm) adhesion map.

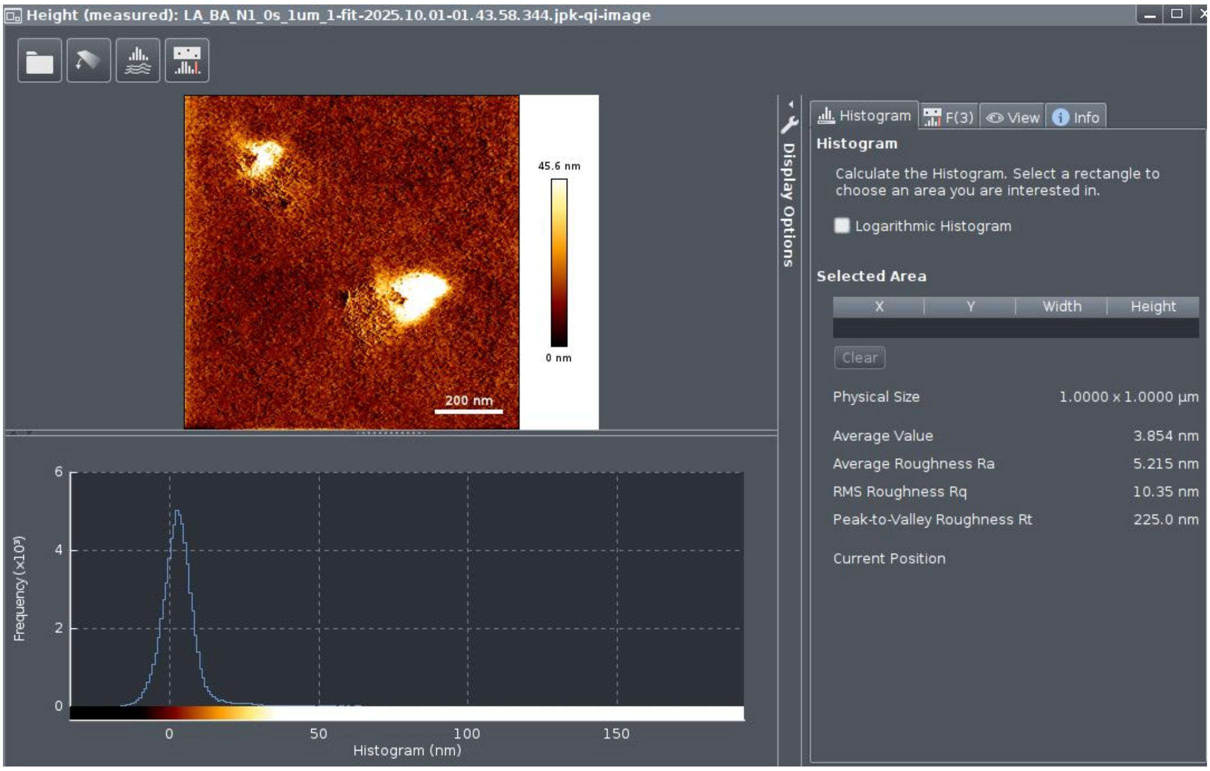

Figure S33. AFM analysis of the un-etched LA_BA film. High-magnification (1 x 1 µm) height topography.

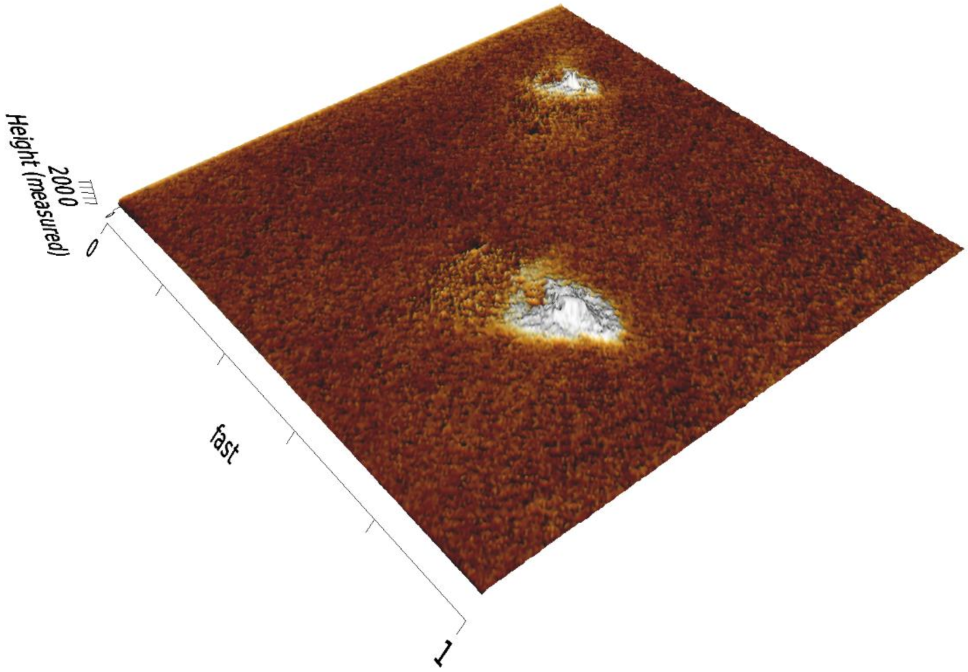

Figure S34. AFM image of the un-etched LA_BA film height topography. High-magnification (1 x 1 µm) height topography.

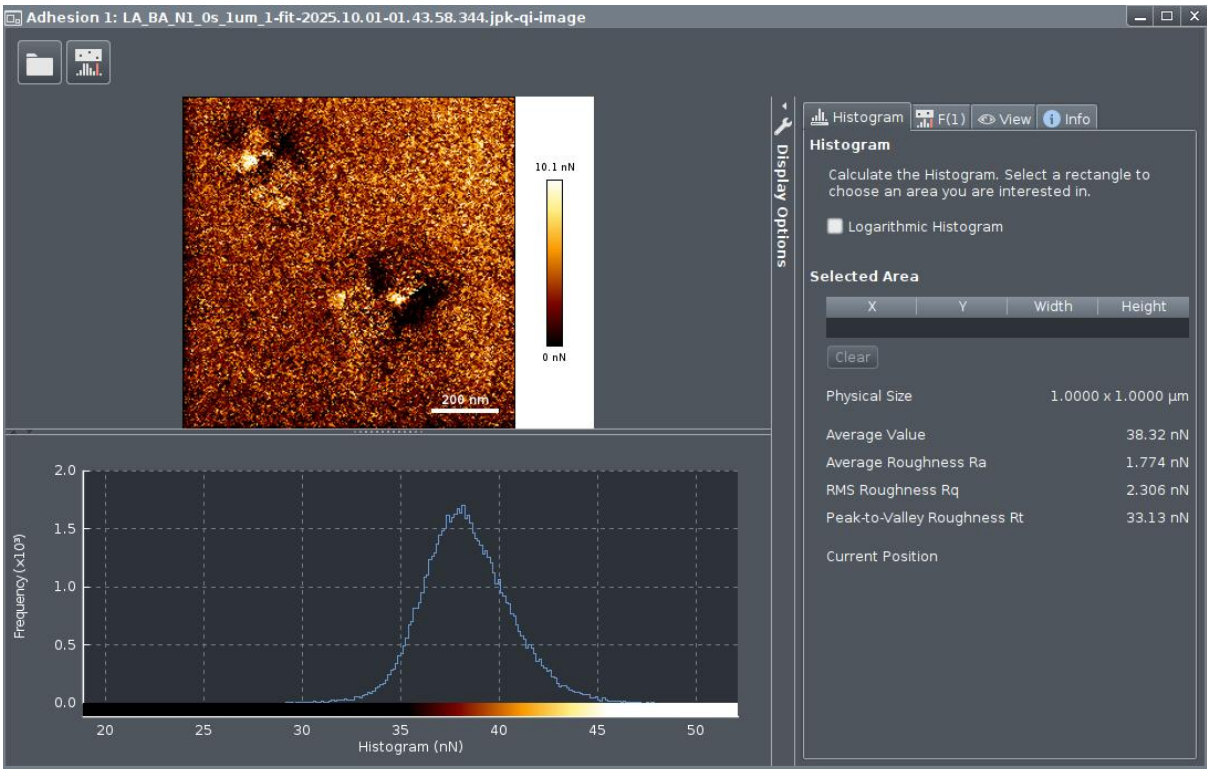

Figure S35. AFM analysis of the un-etched LA_BA film. High-magnification (1 x 1 µm) height topography adhesion map.

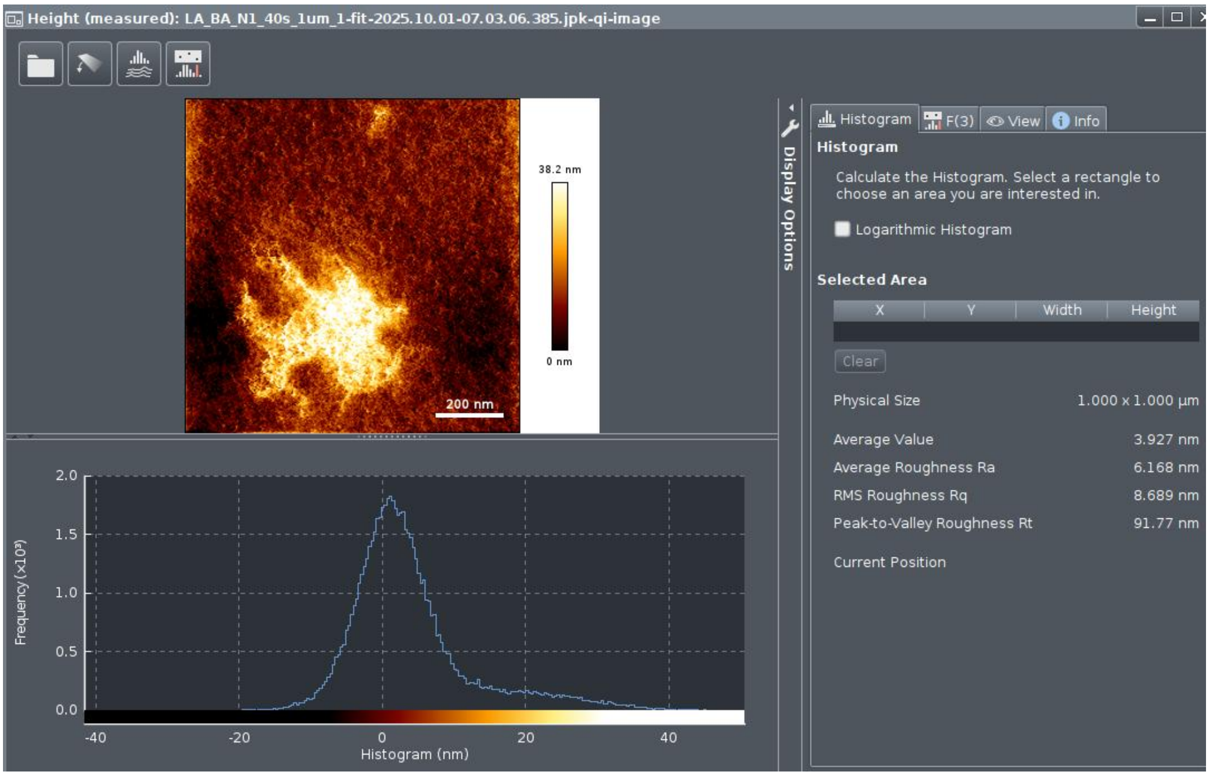

Figure S36. AFM analysis of the LA_BA film with O2 plasma etching for 40 s. High-magnification (1 x 1 µm) height topography.

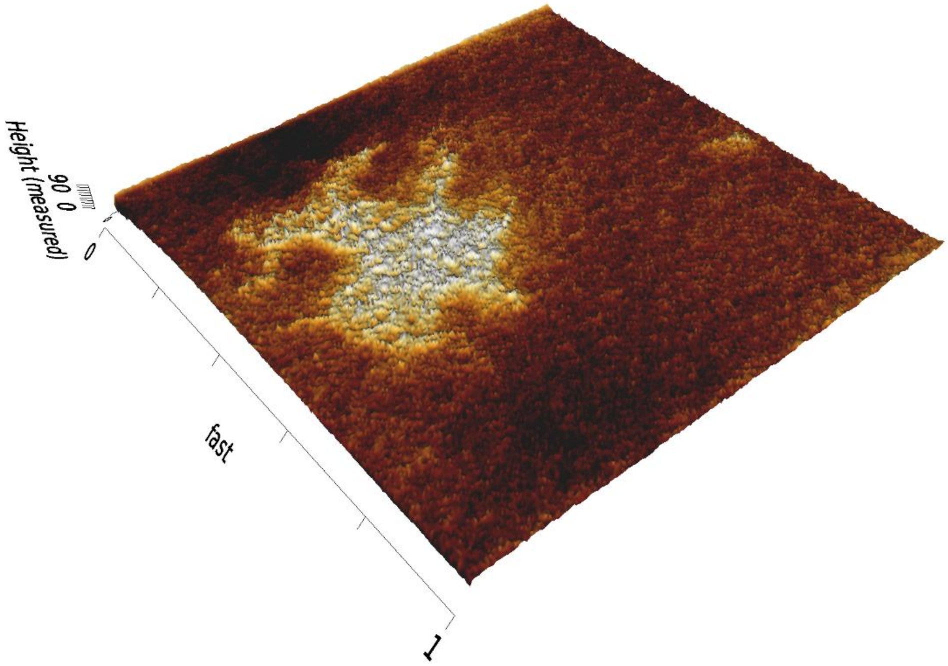

Figure S37. AFM image of the LA_BA film with O_2 plasma etching for 40 s. High-magnification (1 x 1 µm) height topography.

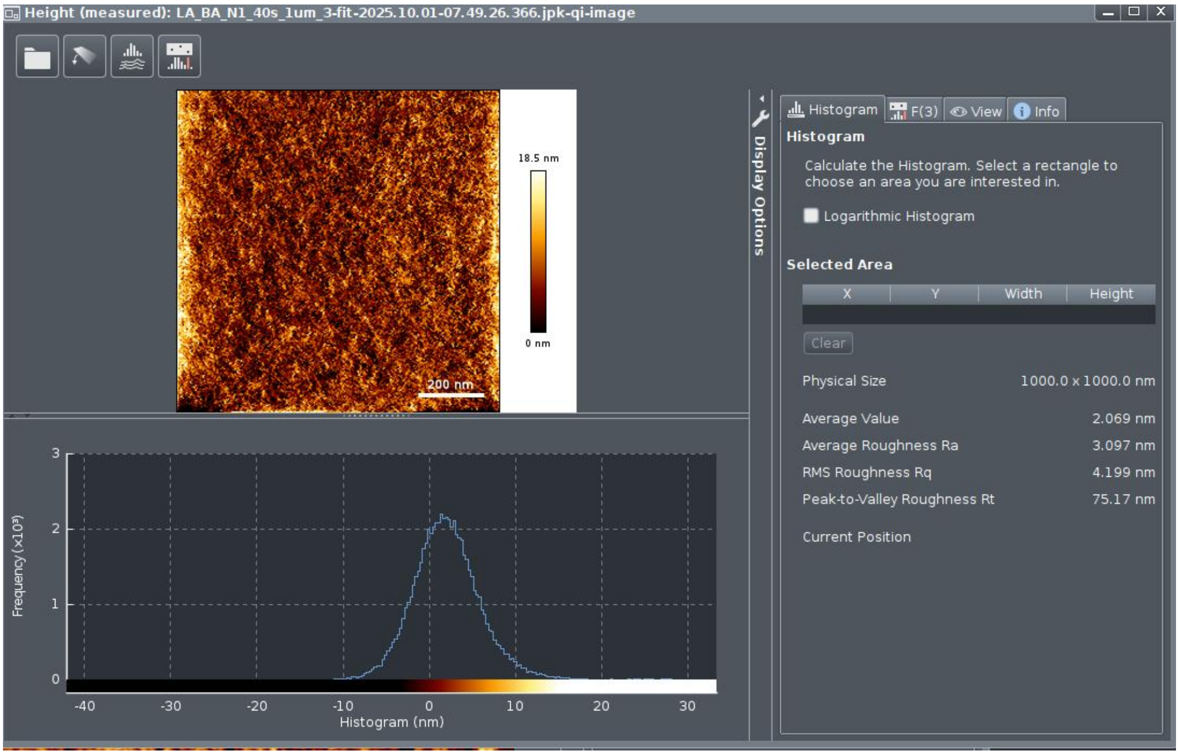

Figure S38. AFM analysis of the LA_BA film with O_2 plasma etching for 40 s. High-magnification (1 x 1 μm) height topography.

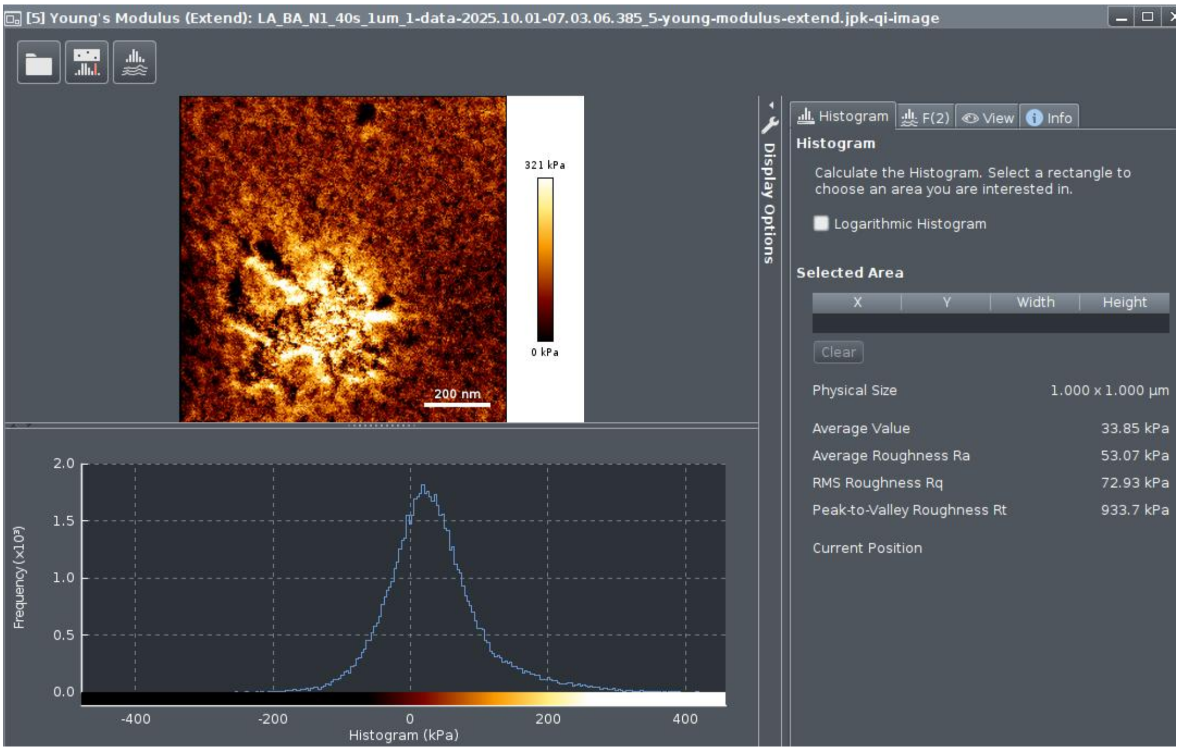

Figure S39. AFM analysis of the LA_BA film with O_2 plasma etching for 40 s. High-magnification (1 x 1 μm) young's modulus map.

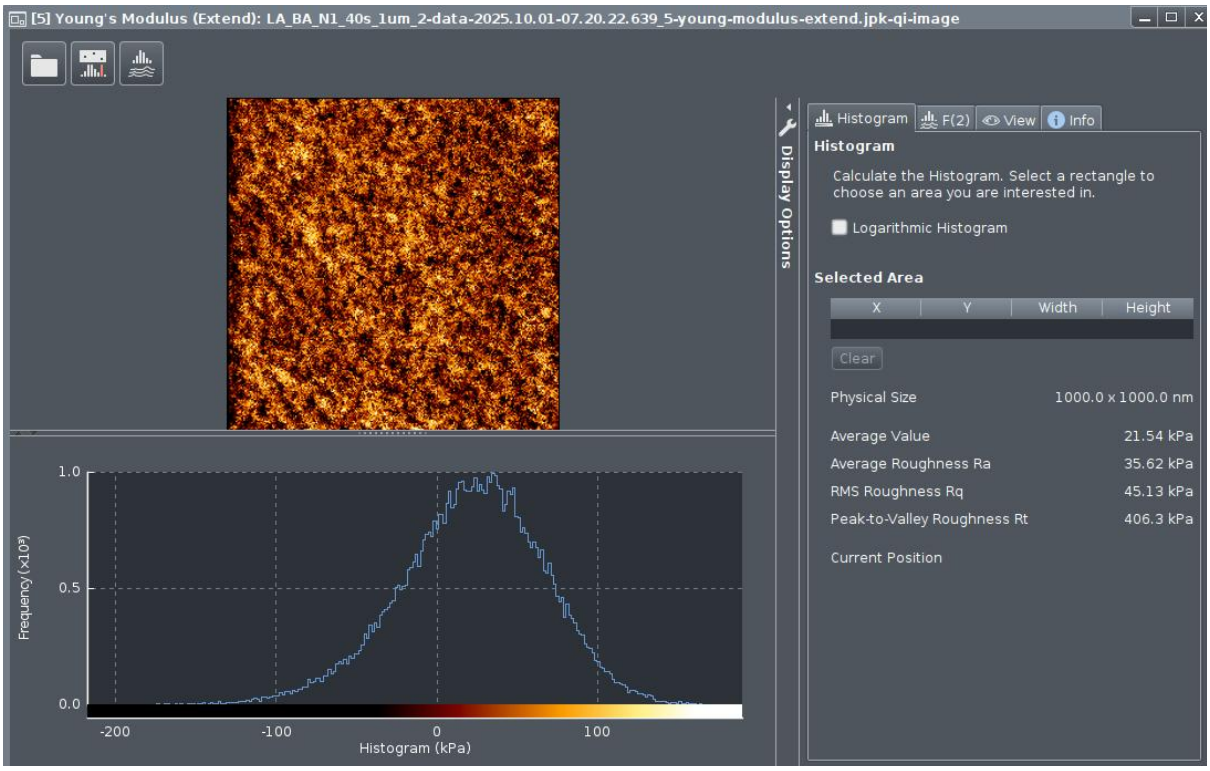

Figure S40. AFM analysis of the LA_BA film with O_2 plasma etching for 40 s. High-magnification (1 x 1 µm) young's modulus map.

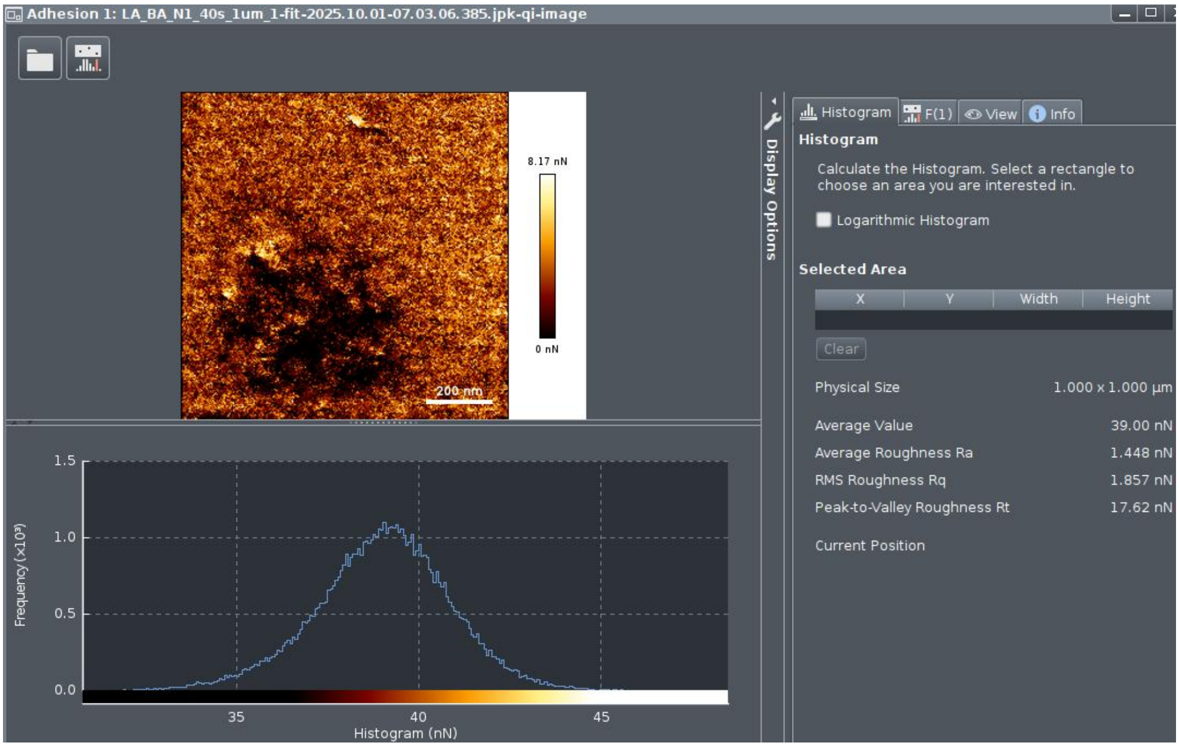

Figure S41. AFM analysis of the LA_BA film with O_2 plasma etching for 40 s. High-magnification (1 x 1 µm) adhesion map.

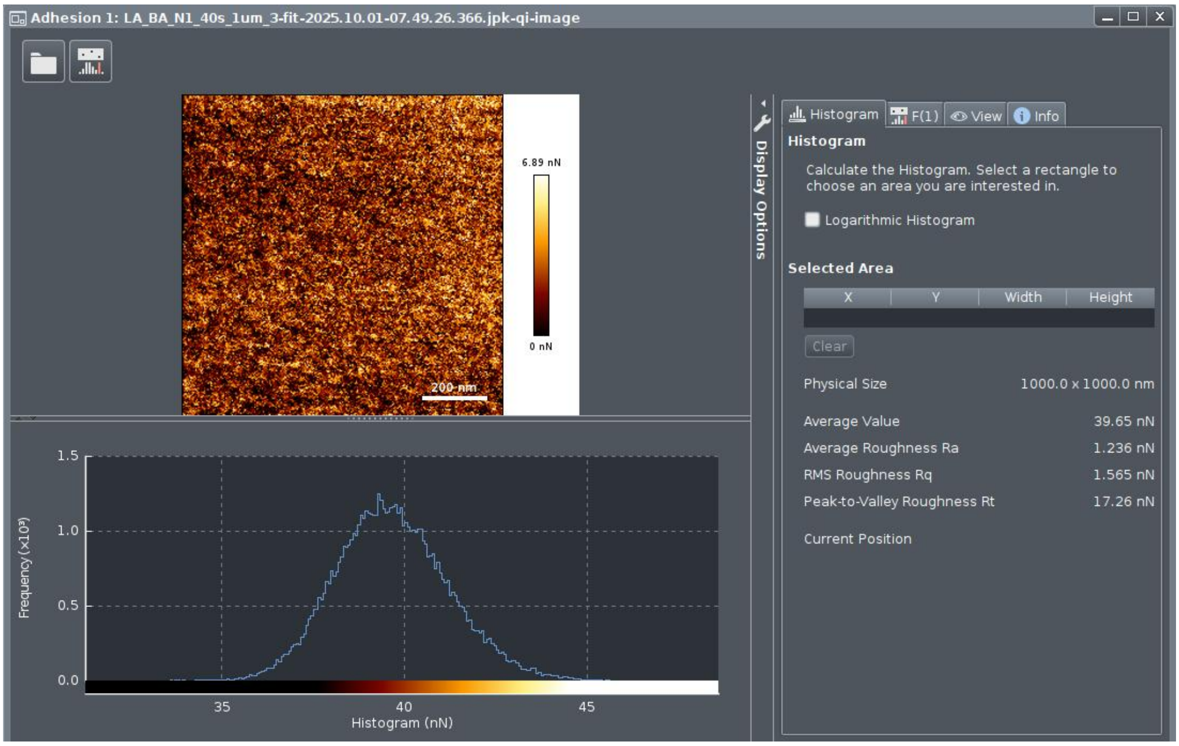

Figure S42. AFM analysis of the LA_BA film with O_2 plasma etching for 40 s. High-magnification (1 x 1 µm) adhesion map.

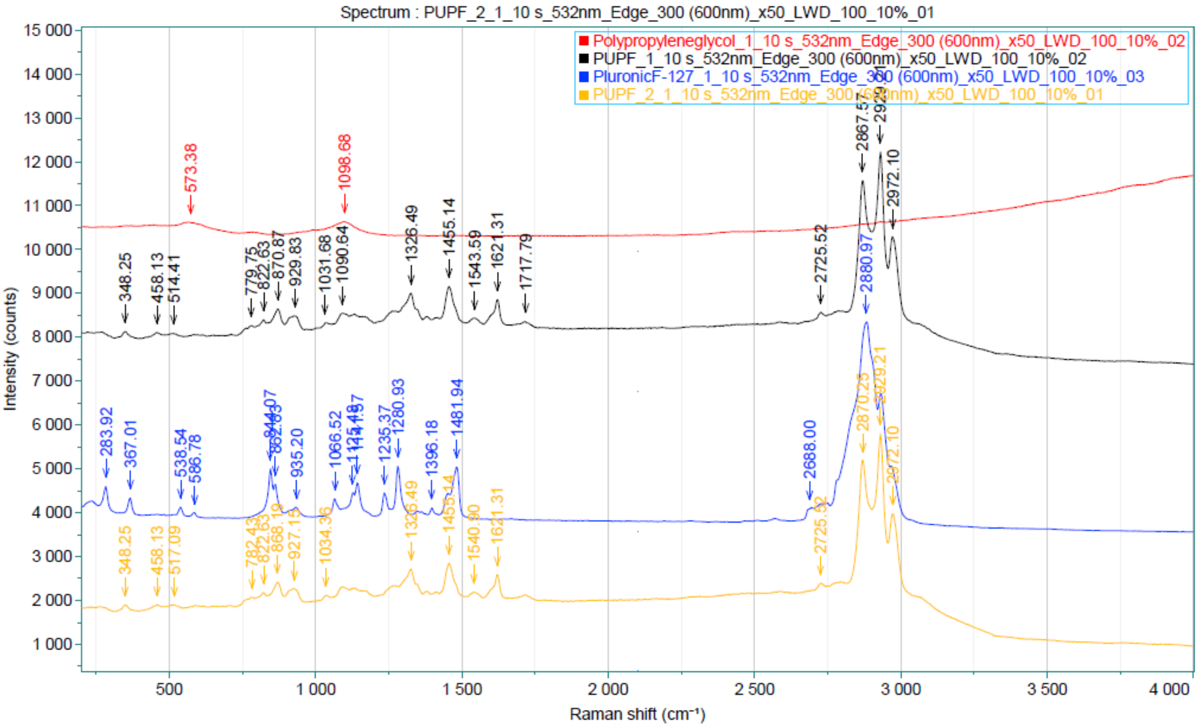

Figure S43. Raman spectra of the LA_BA copolymer (first sample as black line and second sample as yellow line) and its precursors, PrePU (red line) and Pluronic F-127 (blue line).

(a)

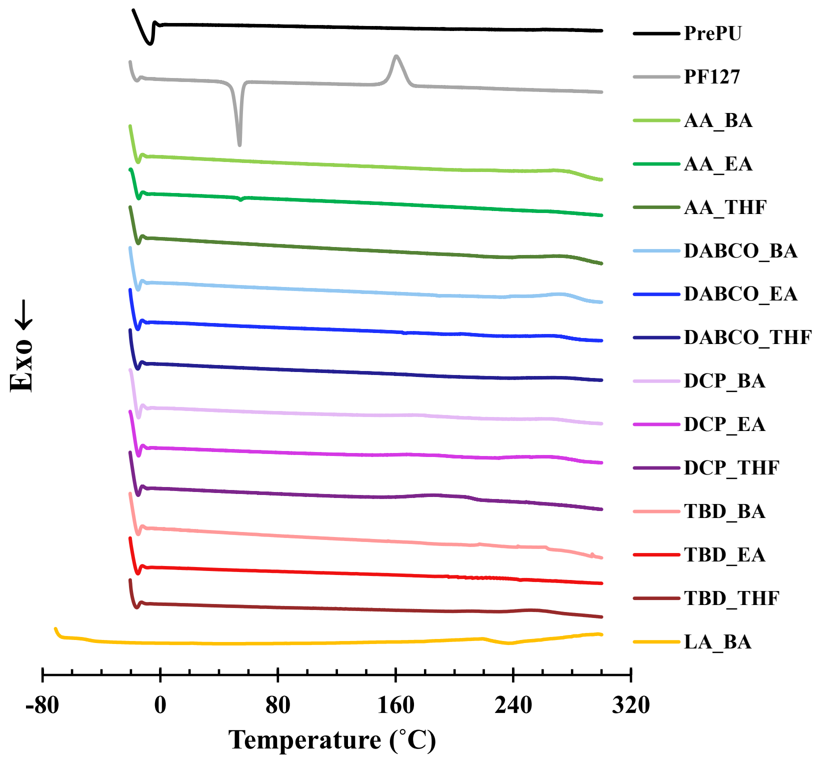

(b)

Sample	T_g, ˚C
PrePU	-0.51
PF127	-16.14
AA_BA	-15.01
AA_EA	-14.66
AA_THF	-14.93
DABCO_BA	-15.03
DABCO_EA	-15.30
DABCO_THF	-15.22
DCP_BA	-14.68
DCP_EA	-14.71
DCP_THF	-15.02
TBD_BA	-14.94
TBD_EA	-15.13
TBD_THF	-15.98
LA_BA	-45.04

Figure S44. Thermal analysis of the synthesized PU-PF films and their precursors (PrePU and Pluronic F-127 (PF127)); (a) DSC thermograms and (b) Comparison of glass transition temperatures (T_g).

(a)

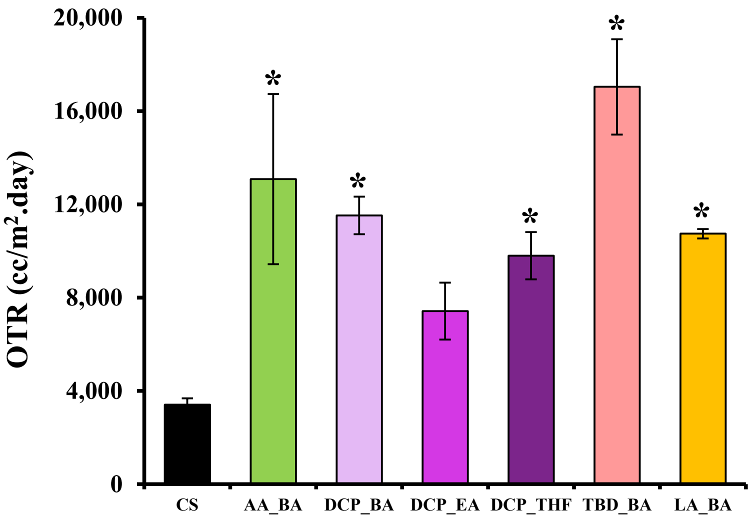

(b)

Sample	OTR (cc/m^2.day)
CS	3,407.9 ± 27.7
AA_BA	13,080.8 ± 3,646.8*
DCP_BA	11,524.7 ± 806.3*
DCP_EA	7,425.0 ± 1,222.5
DCP_THF	9,798.6 ± 1,015.4*
TBD_BA	17,037.6 ± 2,044.5*
LA_BA	10,741.1 ± 201.9*

Figure S45. Oxygen transmission rate (OTR) of the synthesized PU-PF films compared to the commercial PU wound dressing standard (CS); (a) Graphical representation and (b) Corresponding data table. (n=3; * significant higher compared to CS, $p < 0.05$).
